# Genetic containment in vegetatively propagated forest trees: CRISPR disruption of *LEAFY* function in *Eucalyptus* gives sterile indeterminate inflorescences and normal juvenile development

**DOI:** 10.1111/pbi.13588

**Published:** 2021-05-04

**Authors:** Estefania Elorriaga, Amy L. Klocko, Cathleen Ma, Marc du Plessis, Xinmin An, Alexander A. Myburg, Steven H. Strauss

**Affiliations:** ^1^ Department of Forest Ecosystems and Society Oregon State University Corvallis OR USA; ^2^ Department of Biology University of Colorado Colorado Springs Colorado Springs CO USA; ^3^ Department of Zoology and Entomology University of Pretoria Pretoria South Africa; ^4^ Beijing Advanced Innovation Center for Tree Breeding by Molecular Design National Engineering Laboratory for Tree Breeding College of Biological Sciences and Biotechnology Beijing Forestry University Beijing China; ^5^ Department of Biochemistry, Genetics and Microbiology, Forestry and Agricultural Biotechnology Institute (FABI) University of Pretoria Pretoria South Africa; ^6^ Present address: Department of Molecular and Structural Biochemistry North Carolina State University Raleigh NC USA

**Keywords:** *LEAFY*, Eucalyptus, flowering, CRISPR, containment

## Abstract

*Eucalyptus* is among the most widely planted taxa of forest trees worldwide. However, its spread as an exotic or genetically engineered form can create ecological and social problems. To mitigate gene flow via pollen and seeds, we mutated the *Eucalyptus* orthologue of *LEAFY* *(LFY)* by transforming a *Eucalyptus grandis* × *urophylla* wild‐type hybrid and two *Flowering Locus T* *(FT)* overexpressing (and flowering) lines with CRISPR Cas9 targeting its *LFY* orthologue, *ELFY*. We achieved high rates of *elfy* biallelic knockouts, often approaching 100% of transgene insertion events. Frameshift mutations and deletions removing conserved amino acids caused strong floral alterations, including indeterminacy in floral development and an absence of male and female gametes. These mutants were otherwise visibly normal and did not differ statistically from transgenic controls in juvenile vegetative growth rate or leaf morphology in greenhouse trials. Genes upstream or near to *ELFY* in the floral development pathway were overexpressed, whereas floral organ identity genes downstream of *ELFY* were severely depressed. We conclude that disruption of ELFY function appears to be a useful tool for sexual containment, without causing statistically significant or large adverse effects on juvenile vegetative growth or leaf morphology.

## Introduction

Forest plantations cover about 7% of the world’s forests, and one‐quarter of these are comprised of non‐native species and interspecific hybrids (FAO, 2010). These plantings can lead to encroachment and/or genetic admixture into native ecosystems (Donaldson *et al*., 2014; Wilson *et al*., 2009). *Eucalyptus* (family Myrtaceae) is among the most widely planted genera of forest trees, with the largest areas of plantation occurring in Brazil (5.7 million ha), China (4.5 million ha), and India (3.9 million ha) (CIRAD‐FRA *et al*., 2018). Eliminating sexual reproduction from exotic or genetically engineered eucalypts would greatly reduce the potential for spread and invasiveness, while retaining desirable vegetative growth and adaptability traits inherent to the modified genotypes, including their ability to be clonally propagated.

The floral regulatory gene *LEAFY* (*LFY*) encodes both a floral pathway integrator (FPI) and a floral meristem identity (FMI) determinant, and was one of the first floral regulatory genes identified (Coen *et al*., 1990; Weigel *et al*., 1992). It encodes a highly conserved plant‐specific transcription factor found in all land plants, including non‐flowering plants (Moyroud *et al*., 2009; Silva *et al*., 2016), and stretophyte algae (Gao *et al*., 2019). *LFY* is mainly expressed in floral meristematic and primordial organs, yet vegetative expression has also been seen (Ahearn *et al*., 2001; Hofer *et al*., 1997; Molinero‐Rosales *et al*., 1999; Rottmann *et al*., 2000). *ELFY*, the orthologue in *Eucalyptus*, has high expression in the tips of leaf primordia and in flower meristems (Dornelas *et al*., 2004).

The orthologues of *LFY* are present as single‐copy genes in most land plants, except gymnosperms (Moyroud *et al*., 2010; Vázquez‐Lobo *et al*., 2007). Loss‐of‐function mutations lead to sterile and/or late flowering plants in *Arabidopsis* and tomato, and flowerless plants in *Antirrhinum* (Coen *et al*., 1990; Molinero‐Rosales *et al*., 1999; Weigel *et al*., 1992). Because of its high level of conservation and bisexual function, *LFY* is a good target for sexual containment of exotic and weedy species. However, loss‐of‐function mutations in *LFY* have only been characterized in the herbaceous plants *Arabidopsis*, *Antirrhinum,* and tomato, and *LFY* function and expression differ among species. In addition, apart from the partial loss‐of‐function field studies using RNA interference against the *LFY* homologue in poplar (Klocko *et al*., 2016a), we are aware of no in‐depth studies of vegetative development, nor randomized experiments, to estimate impacts on biomass growth rate and vegetative morphology. Thus, it remains unclear whether *LFY* indeed has significant vegetative functions in the species where it shows vegetative expression to the extent that it would compromise its effectiveness as a tool for genetic containment.

The multiple‐year delay of flowering in trees presents a great logistical challenge to genetic studies of floral development. Fortunately, this can be overcome by precocious floral induction using chemical or genetic treatments, including overexpression of *FLOWERING LOCUS T* *(FT)*. Constitutive or inducible overexpression of *FT* elicits early flowering in many herbaceous and woody species, including *Eucalyptus* (Böhlenius *et al*., 2006; Endo *et al*., 2005; Hsu *et al*., 2011; Klocko *et al*., 2016b; Lee *et al*., 2013; Lifschitz and Eshed, 2006; Yamagishi *et al*., 2011). In this study, to understand the effects of CRISPR‐induced mutation of *LFY* on floral structure and function, we retransformed two previously characterized early‐flowering *Eucalyptus* lines that were shown to produce viable pollen and germinable seeds (Klocko *et al*., 2016b). We generated three CRISPR Cas nuclease constructs to induce loss‐of‐function mutations in *ELFY*. Because overexpression of *FT* also adversely affects tree form, we conducted a second greenhouse study in a CRISPR‐mutated wild‐type (non‐FT) background to determine if mutation of *LFY* would affect juvenile vegetative traits and/or growth. We report that sterile, floral‐like indeterminate organs, or an absence of flowers, were produced in all the transgenic events with frameshifts and mutations that removed conserved amino acids, and that there were no statistically significant or large effects of *LFY* disruption on juvenile vegetative growth rate or leaf morphology.

## Results

### *Mutation* *and knockout* *(KO) rates among transgenic events were high*

We generated nine and 59 transgenic events after transforming three CRISPR Cas9 constructs in the WT and the two early‐flowering (i.e., FT‐4 and FT‐30) backgrounds, respectively. For the WT trial, we were interested in determining if knocking out *ELFY* would alter growth or vegetative morphology. For the FT (i.e., early flowering) trial, our intent was to determine if *ELFY* would be an effective target for containment based on its function in relation to flowering in eucalypts. In the WT trial, all nine transgenic events had mutations in both *ELFY* alleles (100% biallelic mutation rate, Table 1). The two Cas9‐control events (i.e., empty vector transgenic controls) did not have mutations on either allele of *ELFY*. In the FT trial, 58 out of 59 FT transgenic events had mutations in both *ELFY* alleles (98.3% biallelic mutation rate, Table 1); a single transgenic event had a mutation only in the *E. urophylla* allele. The nine FT‐Cas9‐control events did not have mutations in either *ELFY* allele. The mean mutation rate per allele among all confirmed transgenic events was 98.5% (Table 1).

**Table 1:** CRISPR mutation rates on a per‐event and per‐allele basis

Population	Total events (alleles)	Alleles modified	No events (%)
WT LFY‐CRISPR	9 (18)	Both alleles	9 (100)
		One allele	0 (0)
		None	0 (0)
FT LFY‐CRISPR	59 (118)	Both alleles	58 (98)
		One allele	1 (2)
		None	0 (0)
**All eucalypt**	**68 (136)**	**Both alleles**	**67 (99)**
		**One allele**	**1 (1)**
		**None**	**0 (0)**

The total values and average rates for all the plants in the study is shown at the bottom in bold.

Based on their translated peptide sequence, 9 of 9 (100%, Table S5, Figure S4) and 53 of 59 (90%, Table S5, Figure S4) events in the WT trial and the FT trial, respectively, had knockout mutations in both alleles. In the FT trial, we expected the remaining six of the 59 (10%, Table S5) events, including the monoallelic mutant, to have normal flowers. Five of these six events had in‐frame mutations in one or both *ELFY* alleles and none of the amino acids removed were highly conserved (events 4‐1, 4‐7, 4‐72, and 30‐41 in Table S7; event 4‐3 was not in the greenhouse study; it had a 6bp deletion in the *grandis* allele and a 3bp deletion in the *urophylla* allele). These five events are referred to as in‐frame‐mutants (FT‐IFM) hereafter.

### *Most* *trees flowered in the FT greenhouse trial*

We selected 42 FT‐CRISPR‐Cas9 (i.e., biallelic KOs and non‐KO biallelic mutants) transgenic events, six FT‐Cas9‐control transgenic events, one FT‐escape‐control transgenic event, and the two original FT‐only transgenic events (i.e., FT‐4 and FT‐30) to study the effect of *ELFY* mutations on floral morphology and reproductive viability. Each of the events had between one and seven ramets (Table S7). The ramets of six of the 42 selected FT‐KO transgenic events (14.3%) did not flower at all. Most ramets of the remaining events (i.e., 32 FT‐KO transgenic events, six FT‐Cas9‐control events, one FT‐escape‐control event, and two FT‐only‐control events) produced reproductive structures (Table S7).

### *FT‐KO* *mutations were stable*

We monitored flowering in the FT ramets for approximately 18 months. We re‐sequenced the *ELFY* alleles of ten FT‐KO transgenic events to test whether the mutations seen early in development had changed because of the overexpression of Cas9 (more than three years elapsed since the first DNA extraction from tissue culture plants to resampling in the greenhouse). For this analysis, we sampled leaves from four different axillary stems. No changes in DNA sequence at the target sites were observed. Also, a greenhouse trial in University of Pretoria in South Africa with several of our FT‐only‐control, FT‐Cas9‐control, and FT‐KO transgenic events showed floral phenotypes that were consistent with those seen in Oregon (Figures S1 and S6), providing further evidence that the mutations and phenotypic effects were stable.

### *FT‐KO* *mutants had either underdeveloped or absent floral organs*

**Figure 1:**
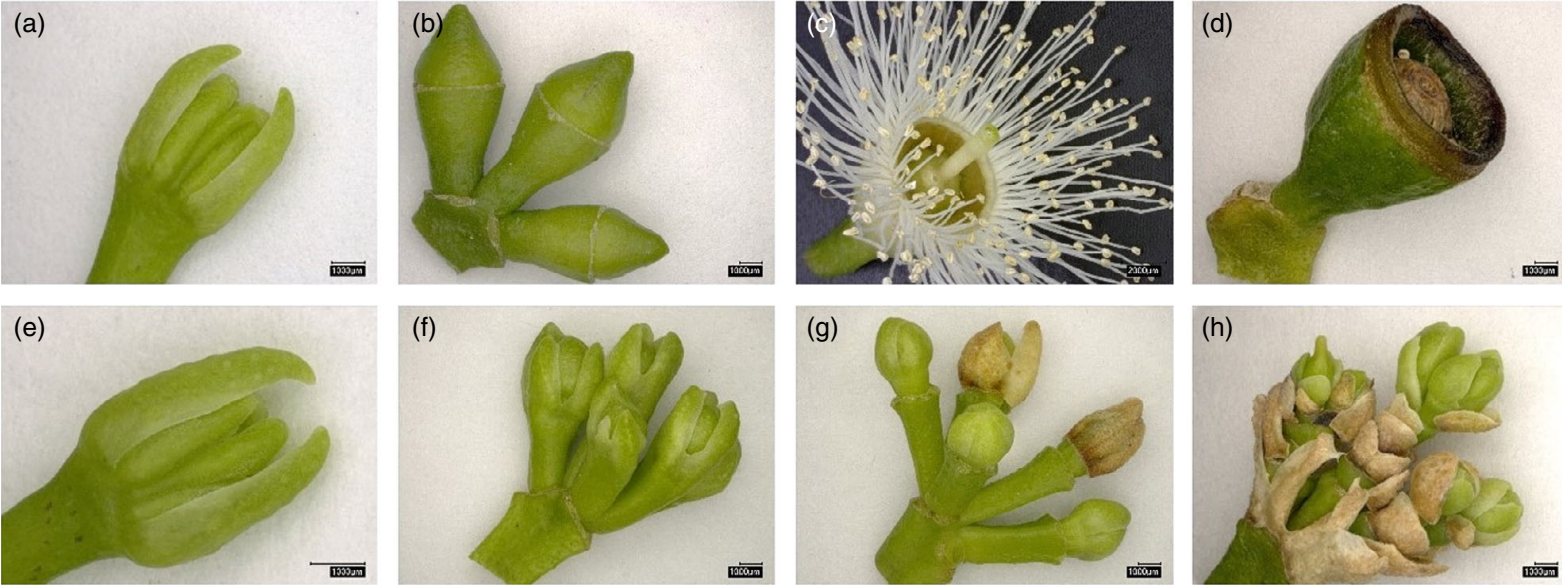
Flower development stages in FT‐controls and FT‐KOs. (a–d) Correspond to flowering tissues from FT‐control events. (a) Umbel with three flowering buds at the earliest recognizable stage. The buds have both calycine and coralline opercula. (b) Umbel with three flowering buds with bracts and calycine opercula shed. (c) Opened flower at anthesis. (d) One capsule harvested about four months after anthesis. (e–h) Correspond to flowering tissues from FT‐KO events. (e) Umbel with three flowering buds at the earliest recognizable stage. At this stage, the flower buds from FT‐KO events are indistinguishable from the flowering buds of FT‐controls. (f) Umbel with four flowering buds with bracts shed. At this stage, flowering buds from FT‐KO events are recognizably different from those of FT‐controls. (g) Umbel with five mature buds generating and shedding layers of pedicels and bracts. (h) Umbel with three stacked floral‐like organs showing indeterminacy and retention of senescent bracts eight to ten months after buds were discernible as in (f). All scale bars measure 1000μm.

All 42 FT‐CRISPR‐Cas9 transgenic events had biallelic mutations (Table S7). However, four events (the FT‐IFM events: 4‐1, 4‐7, 4‐72 and 30‐41) were predicted to have normal flowers based on their peptide modifications (either in‐frame deletions of non‐conserved amino acid or in‐frame insertions of an amino acid; Figure S12). The ramets from the four FT‐IFM events produced flowers identical to those found in the six FT‐Cas9‐control events (i.e., Cas9‐4‐6, Cas9‐4‐8, Cas9‐4‐16, Cas9‐4‐20, Cas9‐30‐5 and Cas9‐30‐14), the one FT‐escape‐control event (FT‐4‐escape), and the two FT‐only‐control events (FT‐4 and FT‐30) (Figure 1c, Figures S1, Figure S4a, Video S1). These flowers had a central pistil and a staminal ring at the base of the hypanthium (Figure 1c, Figure S1a). They also appeared to be capable of secreting nectar at the base of the hypanthium wall as observed in wild‐type eucalypts (e.g. Figure S1b–d). Out of the remaining 38 FT‐CRISPR‐Cas9 transgenic events, none of the ramets of four events with frameshift mutations and two events with N‐terminal deletions flowered at all (FT‐KO events 4‐17,4‐18, 4‐24, 4‐41, 4‐88, and 30‐19; Table S7).

The remaining 32 FT‐CRISPR‐Cas9 transgenic events were all confirmed knockout events (i.e., the ELFY transcription factor was not functional). The flowering ramets of these 32 FT‐KO transgenic events had bud‐like structures with repeated bract‐like and pedicel‐like organs (Figure 1g, h, Figures S4b, S5, S6d, S9b, and Video S2), with a range in phenotypes that went from underdeveloped bisexual floral‐like structures with two to three repeated layers of bract‐like and pedicel‐like organs with underdeveloped (i.e., sterile) stamens and underdeveloped (i.e., sterile) gynoecia (Figure S5a–i) to bud‐like structures with many repeated layers of bracts and pedicels with no reproductive organs at all (Figure 1h, Figure S5j–r).

Among these 32 FT‐KO transgenic events, there were two main types of predicted peptide modifications: in‐frame N‐terminal deletions and frameshifts (Table S7, column Peptide Modification). In one or both alleles of five FT‐KO transgenic events (events 4‐46, 4‐55, 30‐30, 30‐33, and 30‐42), almost the entire N‐terminal was removed with a large deletion of 225, 228, 261, or 264 bp (see space in between black arrows in Figure S11). The floral‐like (or bud‐like) structures in these events had two‐ or three‐layered bracts and pedicels followed by underdeveloped (i.e., sterile) stamens and underdeveloped (i.e., sterile) gynoecia (Figure S5i).

The remaining 27 FT‐KO transgenic events had frameshifts resulting from small indel mutations in one or both target sites of each allele (Table S7). Nine of the 27 events presented only underdeveloped (i.e., sterile) gynoecia after three‐ to five‐layered bracts and pedicels (Figure S5a‐h). And the remaining 18 events had mostly no signs of reproductive organs in their multiple layered floral‐like structures of repeated bracts and pedicels (Figures S4b, S5j‐l, S6d, Video S2). These floral‐like structures would accumulate anywhere between seven and eleven layered bracts and pedicels (Figures S4b, S5j‐l, S6d, Video S2) before becoming woody and falling off. On occasion, some of these long‐lived structures (>5 months alive on stem) would eventually produce underdeveloped (i.e., sterile) gynoecia after 8 or more layers (Figure S10c,d). However, most of the long‐lived flowers never produced any reproductive organs (Figure S10a,b). By contrast, wild‐type flowers usually developed over three to four months, with the seed capsules requiring an additional four to five months to mature and dehisce (Hodgson, 1976a, 1976b).

### *Changes* *in expression of flowering genes due to KO*

**Figure 2:**
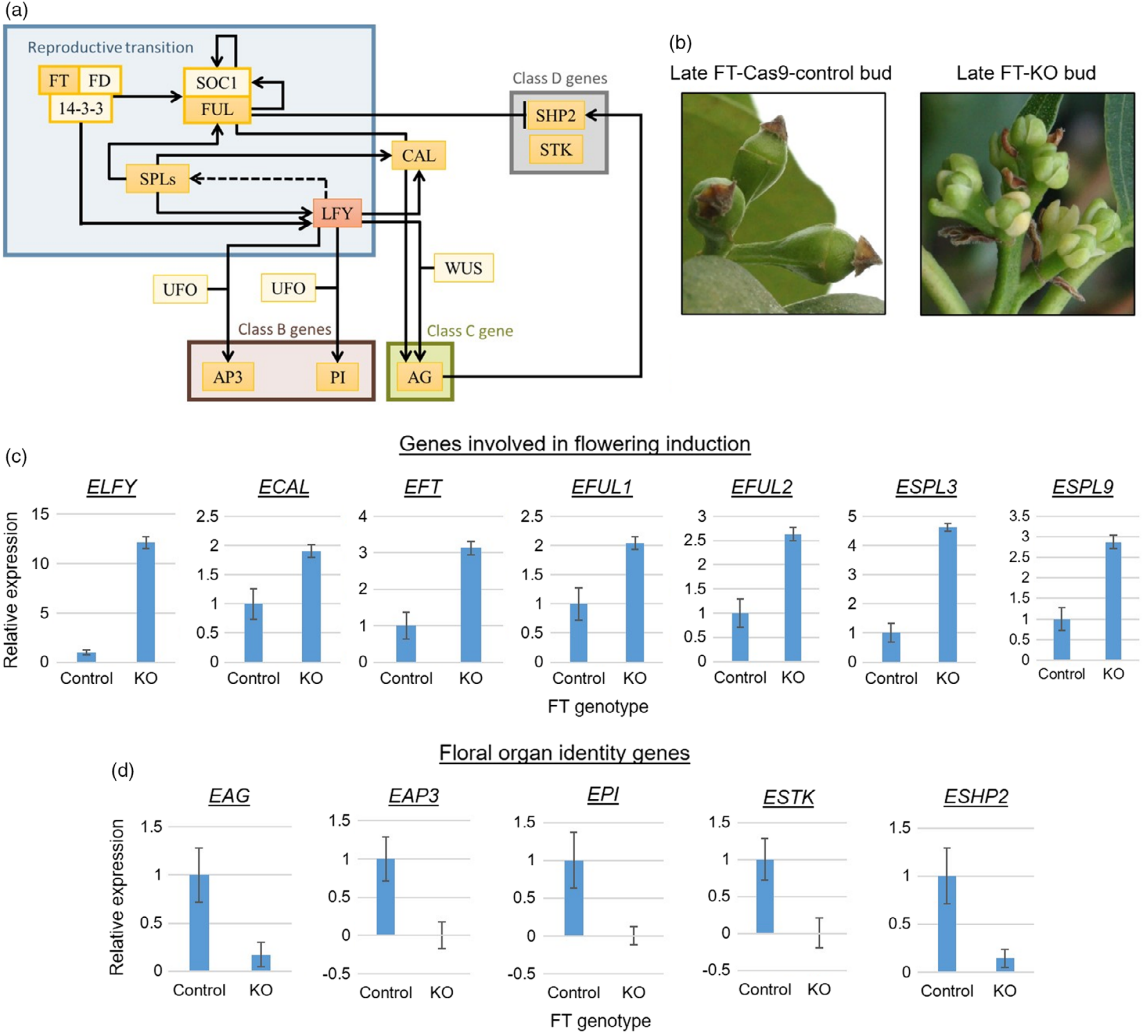
Transcriptional network related to *ELFY*, and its expression from qPCR in late FT‐control flower buds or late FT‐KO flower‐like buds. (a) Simplified genetic pathway from *Arabidopsis* (described in the introduction). We performed gene expression analysis on genes with an orange (*LFY*) or yellow fill. (b) Photos of the late flower or floral‐like buds. (c) Late bud gene expression of genes involved in flowering induction seen in FT‐control and FT‐KO transgenic plants. (d) Late bud gene expression seen in FT‐control and FT‐KO transgenic plants of organ identity genes downstream of *ELFY*. The average fold‐change in expression for (c) and (d) was calculated as a ratio to the expression of the controls, which was set to 1. There were six biological replicates for the FT‐KO transgenic events and three for the FT‐controls. All reactions had three technical replicates. Error bars represent ± SE of means. Gene expression was significantly different in all genes when comparing mean expression for the FT‐control events to the FT‐KO events (*P* < 0.05, two‐tailed Student’s *t*‐test).

We wanted to see if gene expression would help interpret the modifications seen in the reproductive organs of our mutants. Differences in gene expression of twelve flowering genes including *ELFY* were analysed for six FT‐KO events (events 30‐2, 30‐10, 30‐11, 30‐31, 30‐40 and 30‐45) and three FT‐control events (FT‐only‐control event 30‐3 and FT‐Cas9‐control events Cas9‐30‐5 and Cas9‐30‐14). We selected buds from the FT‐control events that had just shed or were shedding their calycine operculum and were about a month away from anthesis (Figure 2b). We selected buds from the FT‐KO events that were shedding or had just shed their first layer of bract‐like organs (Figure 2b). We attempted to select reproductive tissues that were at the same age, but it is important to understand the difficulty of this task given the substantial differences in morphology between the FT‐control events and the FT‐KO events.

*ELFY* expression was significantly higher in the FT‐KO events than in the FT‐control events (mean of 636% higher expression than controls, *P* = 0.02; Figure 2c). Expression of six genes (i.e., *ECAL*, *EFT*, *EFUL1*, *EFUL2*, *ESPL3*, and *ESPL9*) that act upstream or at a similar stage of development to *ELFY* was higher in the FT‐KO transgenic events than in the FT‐control events (Figure 2c). When comparing the expression between the FT‐control events and the FT‐KO events, the FT‐KO transgenic events had a mean fold‐change in gene expression of 3.0 for *EFT* (*P* = 0.003), 4.4 for *ESPL3* (*P* = 1.0E‐4), 2.9 for *ESPL9* (*P* = 0), 1.9 for *ECAL* (*P* = 0.004), 2.1 for *EFUL1* (*P* = 0.002), *and* 2.6 for *EFUL2* (*P* = 0).

Meanwhile, expression of five FOI genes that are induced by *ELFY*, directly or indirectly, (i.e., *EAP3*, *EPI*, *EAG*, *ESHP2* and *ESTK*) was significantly lower in the FT‐KO transgenic events than in the FT‐control events (Figure 2d). When comparing the expression levels in the FT‐control events to the FT‐KO transgenic events, the FT‐control events had a mean fold difference in gene expression of 2,500 for *EAP3* (*P* = 0.006), 2.8 for *EPI* (*P* = 3.0E‐4), 5.6 for *EAG* (*P* = 0.009), 6.6 for *ESHP2* (*P* = 0.01), and 178.6 for *ESTK* (*P* = 0.01).

To analyse how *ELFY* expression in FT‐control and FT‐KO transgenic plants changed during floral development, we also compared the expression of *ELFY* in early‐ and mid‐bud development from FT‐Cas9‐control event Cas9‐30‐14 and FT‐KO events 30‐10 and 30‐11. Buds were sampled as soon as they were recognizable as flowering buds and about a month later when the bracts were beginning to dehisce (Figure S7c). The early‐ and mid‐buds looked about the same in FT‐control events and FT‐KO events (Figure S7c). Although the absolute *ELFY* expression levels varied widely among all events, the overall pattern in the FT‐controls was different from that in the FT‐KOs (Figure S7a,b). The FT‐controls showed a monotonic decline over developmental time while the two FT‐KOs did not show substantially reduced *EFLY* expression over time.

### *KOs* *did not differ in vegetative traits*

**Figure 3:**
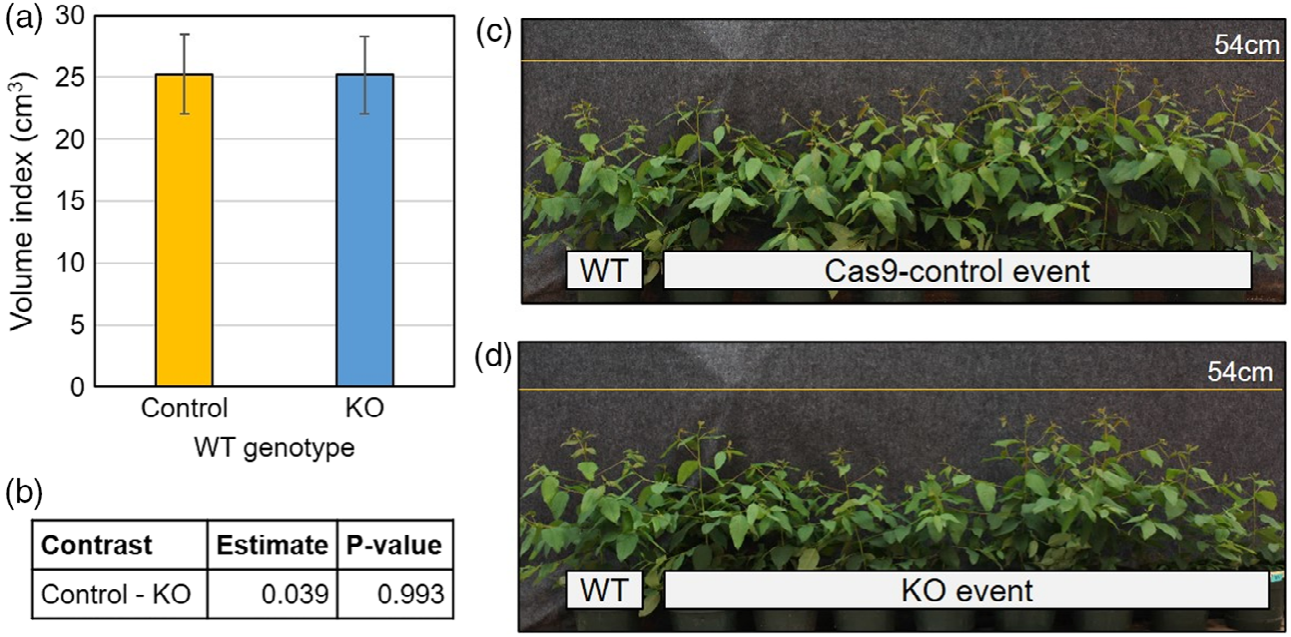
Stem volume growth and plant form appear to be unaffected by knockout of *ELFY* in the WT greenhouse trial. (a) Mean stem volume index (height × diameter^2^) for the knockouts (KOs) and the controls. Error bars represent ± SE of means. (b) Table of estimated mean differences and p‐values corresponding to a Student’s *t*‐test on the means for each contrast. (c) Image of potted reference WT ramet and the six ramets corresponding to Cas9‐control event 42. (d) Image of potted reference WT ramet and eight ramets of KO event 167. The yellow lines in both photographs are at 54 cm height.

The purpose of the greenhouse WT trial was to determine if *ELFY* had any vegetative function that would affect vegetative growth or morphology. For this trial, we had nine KO events and six control events made up of two escape‐control events, three Cas9‐control events, and WT. The nine KO events had a total of 41 ramets. The controls had 37 ramets: 12 ramets corresponding to the escape‐control events, 18 ramets corresponding to the three Cas9‐control events, and seven WT ramets (Table S6). In total, we monitored 78 ramets, and each transgenic event had between three and six ramets (Table S6). When analysing the different traits measured, we found no significant differences in any comparisons between KO events and control events in volume index, leaf area, perimeter, leaf dry weight, and specific leaf weight (*P* > 0.05; Figure 3, Figure S13). However, unlike the results for volume index where *P*‐values were above 0.9, leaf area and the related traits of leaf perimeter and dry weight had *P*‐values of 0.125 or 0.237, thus very weakly supporting a possible reduction in leaf size in KO vs. control plants.

**Figure 4:**
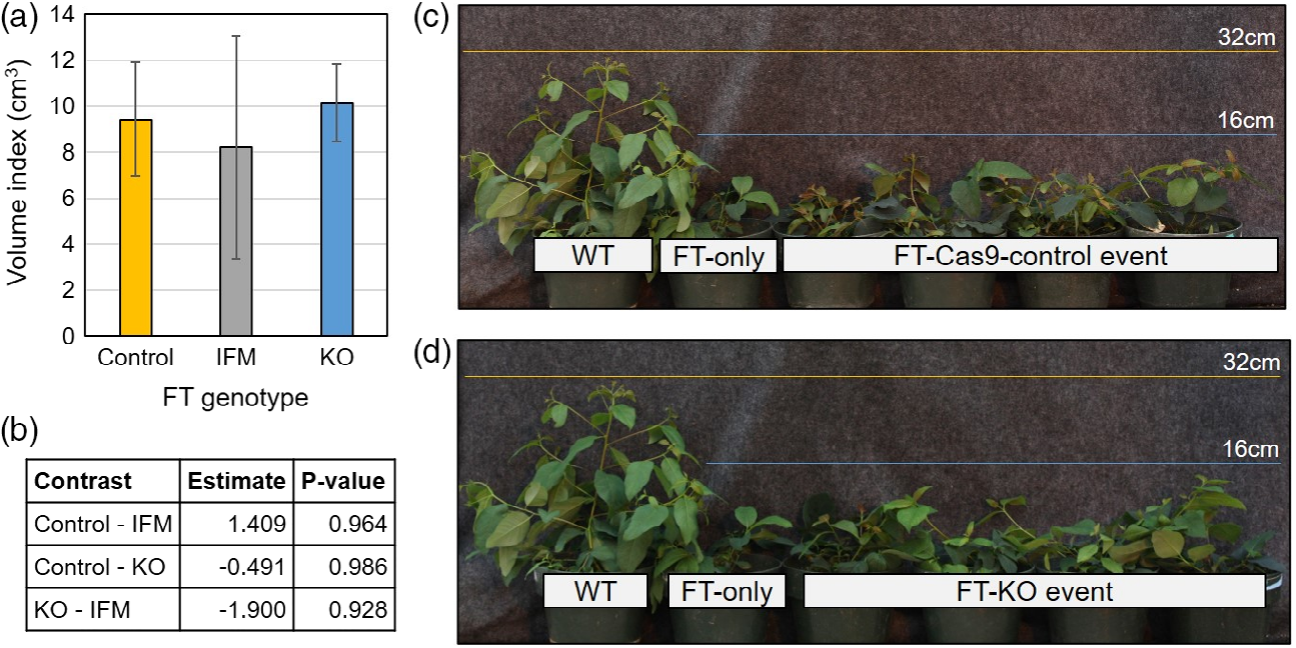
Stem growth was reduced in plants that flowered precociously due to *FT* overexpression but did not differ due to *ELFY* mutagenesis. (a) Mean stem volume index (height × diameter^2^) and its standard error for the FT‐KO events, the FT‐IFM events, and the FT‐control events. The control events included the FT‐only‐control events, the FT‐Cas9‐control events and the FT‐escape‐control events. Error bars represent ± SE of means. (b) Table of estimated mean differences and *P*‐values corresponding to a Student *t*‐test on the means for each contrast. (c) Image of potted reference WT ramet, FT‐only‐control flowering reference and the four ramets corresponding to FT‐Cas9‐control event 4‐16. (d) Image of potted reference WT ramet, FT‐only‐control flowering reference and the four ramets of FT‐KO event 30‐40. The yellow and blue lines in both photographs are at 32 and 16 cm height, respectively. IFM, in‐frame mutant; KO, knockout; WT, wild type.

We also compared vegetative traits among the early‐flowering trees (i.e., the FT trial). The overexpression of *FT* eliminated the apical dominance in all these trees and as a result they had a bush‐like form. After analysing volume index, SPAD values, and the four leaf traits (i.e., leaf area, leaf perimeter, leaf dry weight, and specific leaf weight), we found no significant differences between the FT‐KO transgenic events and the FT‐control events (Figure 4, Figure S14). We also found no differences in mean between the FT‐IFM (i.e., in‐frame‐mutant) events and the FT‐control events in volume index, SPAD, leaf perimeter, leaf dry weight, and specific leaf weight (Figure 4, Figure S14). However, when contrasting the mean leaf area of the FT‐IFM events to the FT‐control events, they were found to be significantly different (P = 0.03; Figure S14).

## Discussion

All three vectors were nearly 100% effective at inducing mutations at endogenous target sites. Our average predicted knockout rate based on the peptide modifications was 91% (Table S5). We also saw high mutation and predicted knockout rates in hybrid poplar when targeting the gene orthologous to *LFY* and the two genes orthologous to *AG* (Elorriaga *et al*., 2018). Mutation efficiencies of endogenous genes in plants vary between 0.1% and 100% (Bewg *et al*., 2018; Dai *et al*., 2020; Ghogare *et al*., 2019; Xu *et al*., 2019). Nonetheless, similar to our results, editing rates of 100% were seen in stably transformed cassava (Odipio *et al*., 2017), grapevine (Ren *et al*., 2016), maize (Lee *et al*., 2019), poplar (Zhou *et al*., 2015), rice (Xie *et al*., 2015), and tomato (Zhang *et al*., 2020).

There were no statistically significant or strong differences in any of the vegetative traits that suggested that the KO of *ELFY* function had vegetative consequences. We know of no other randomized studies of either knockouts or knockdowns of a *LFY* orthologue with respect to its vegetative performance in the field or greenhouse, with the exception of a small field study from our own laboratory on hybrid poplar (Klocko *et al*., 2016a). There, no statistically significant differences were detected between strong RNAi suppression events and non‐suppressed transgenic trees in height, diameter at breast height, or volume index (Klocko *et al*., 2016a). We were particularly concerned about the effects on vegetative performance in *LFY* knockouts because of the reports of significant *LFY* expression in vegetative meristems in *Eucalyptus* *(*Dornelas *et al*., 2004 *);* however, vegetative expression has also been reported in *Arabidopsis*, *Impatiens*, pea, petunia, tobacco, and tomato (Blazquez *et al*., 1997; Bradley *et al*., 1997; Hempel *et al*., 1997; Hofer *et al*., 1997; Kelly *et al*., 1995; Molinero‐Rosales *et al*., 1999; Pouteau *et al*., 1997; Souer *et al*., 1998; Weigel *et al*., 1992). Despite expression in vegetative meristems, effects on leaf morphology have only been reported in *unifoliata* mutants (pea) (Blazquez *et al*., 1997; Bradley *et al*., 1997; Dornelas *et al*., 2004; Hempel *et al*., 1997; Hofer *et al*., 1997; Kelly *et al*., 1995; Molinero‐Rosales *et al*., 1999; Pouteau *et al*., 1997; Souer *et al*., 1998; Weigel *et al*., 1992).

Leaf area was the only trait that showed a statistically significant difference among groups when comparing the mean of the FT in‐frame‐mutation (FT‐IFM) events to the mean of the FT‐control events. The significance of this difference is unclear, especially as the FT‐IFM events had the smallest number of events and replicates, and the FT‐KO transgenic events that were of primary interest to this study did not differ from the FT‐control group. The FT‐IFM group only had three events in the greenhouse and four events in the entire study, and each of the three events in the greenhouse had a unique type of mutation and associated amino acid modification. In this study, in‐frame mutations were not as common as frameshifts or large deletions, similar to what we reported in poplar (Elorriaga *et al*., 2018). When comparing vegetative performance of the three individual FT‐IFM events to each control event (i.e., two FT‐only‐control events, six FT‐Cas9‐control events and one FT‐escape‐control event), we found that FT‐IFM event 4‐1 and FT‐Cas9‐control event Cas9‐30‐5 had significantly smaller leaves (area and perimeter) than the other events (Figure S15). Also, the FT‐only‐control event FT‐30 appeared to have higher chlorophyll density than all other events (Figure S15). The reasons for these results are unclear; they may be due to the amino change in the FT‐IFM event, an insertion or somaclonal event in either of the events, or due to chance alone given the large number of event comparisons examined. We have seen a significant number (ca. 2–5% of events) of leaf shape and size modifications in other greenhouse studies of CRISPR‐modified *Eucalyptus* (S.H. Strauss and B. Zahl, unpublished data).

After a year and a half in the greenhouse, all the ramets of six predicted FT‐KO events did not flower at all. From visual inspection, all these ramets had normal tree form (i.e., the same form as the trees in the WT trial), whereas *FT‐*expressing plants had a distinctive dwarf and highly branched form (e.g. Figure 4c,d). We suspect that the *FT* transgene was silenced or attenuated in signal sometime after transformation with the CRISPR Cas9 transgene given previous similar experiences with *FT* overexpressing poplar lines ceasing to flower and resuming normal form in our laboratory (and not restarting flowering or modified form even after repeated propagation and completion of a dormancy cycle).

The remaining 32 FT‐KO transgenic events produced sterile ‘flowers’. In general, we found that the events would produce underdeveloped female ‘flowers’ (‘UFF’ under floral phenotype in Table S7; Figure S5a‐h) or organless ‘flowers’ (‘ORGANLESS’ in Table S7; Figure S5j‐r) when both alleles were mutated with frameshift mutations (‘FS’ under peptide modification in Table S7), there were deletions of conserved amino acids (‘CAAD’ under peptide modification in Table S7, Figure S11), or a combination of these.

What we are calling underdeveloped female ‘flowers’ (i.e., later‐arising ‘flowers’ made up of repeated bracts and underdeveloped gynoecia) have been documented in knockouts and knockdowns of *LFY* homologues in several other plant species including *Arabidopsis*, California poppy, *Lotus japonicus*, and pea (Dong *et al*., 2005; Hofer *et al*., 1997; Weigel *et al*., 1992; Wreath *et al*., 2013). These later‐arising ‘flowers’ generally consist of sepal and carpel‐like organs and lack petals and stamens. This is likely because LFY must be bound to UNUSUAL FLORAL ORGANS (UFO) to up‐regulate expression of *AP3* and *PI*, the B‐class genes needed for petal and stamen formation (Chae *et al*., 2008; Lee *et al*., 1997; Levin and Meyerowitz, 1995; Moyroud *et al*., 2009). Strong knockouts with frameshift mutations may have modified the LFY peptide sequence so much so that the interaction with UFO was no longer possible.

In this ‘UFF’ category, there were two FT‐KO events, 30‐6 and 4‐8, that were missing between one and three highly conserved amino acids. In 30‐6, the *E. grandis* allele had a 6 bp deletion that removed a glutamic acid (E22 in *Eucalyptus* and E54 in alignment; Figure S11) and an alanine (A23 in *Eucalyptus* and A55 in alignment; Figure S11). In the same event, the *E. urophylla* allele had a 3 bp deletion that caused the same alanine (A23 in *Eucalyptus* and A55 in alignment; Figure S11) to be removed and the adjacent glutamic acid to be replaced by an aspartic acid (E22D in *Eucalyptus* and E54D in alignment; Figure S11). In event 4‐8, the *E. grandis* allele had a 9 bp deletion that removed a phenylalanine, a glutamic acid, and an alanine (F21, E22, A23 in *Eucalyptus* and F53, E54, and A55 in alignment; Figure S11). In the same event, the *E. urophylla* allele had a 1 bp insertion that induced a frameshift and introduced a stop codon at the 91st AA position. The phenylalanine and alanine sites are highly conserved among most plant species and eudicots, respectively; thus, they are likely essential to the interaction in *ELFY* dimers (Figure S11). These two events had a flowering phenotype similar to many events with frameshifts in both alleles that completely disturbed the peptide sequence. In this limited study, without replication of specific amino acid deletions, it is not possible to clearly identify the types of mutations that caused some events to have UFF versus organless ‘flowers.’

If both alleles were mutated and at least one of the alleles had an in‐frame large deletion that removed most of the N‐terminal, the event would produce underdeveloped bisexual flowers (UBF in Table S7; Figure S5i). These events had their C‐terminals intact with the consequence that the plants mostly had flowers with sterile reproductive organs that appeared early on in development (i.e., early organs in Table S7). We believe that part of the differences in floral phenotypes among our FT‐KO transgenic events was due to partial *ELFY* function in the events with intact C‐terminals compared to FT‐KO transgenic events with completely disturbed *ELFY* alleles. All LFY transcription factors in the plant kingdom have two conserved domains; an N‐terminal dimerization domain (Sayou *et al*., 2016; Siriwardana and Lamb, 2012) and a C‐terminal DNA‐binding domain (Hamès *et al*., 2008). And recently, the second exon (located in between the first exon that codes the N‐terminal domain and the third exon that codes for the C‐terminal domain) was found to be important in the induction of floral fate in *Arabidopsis* (Zhu *et al*., 2020).

Siriwardana and Lamb (2012) found that removing or modifying certain amino acids in the N‐terminal domain eliminated LFY function *in planta* completely. These modified alleles could not complement *lfy‐6*, and the plants produced sterile flowers with sepal‐like and ovule‐like organs after bolting. In similar experiments, Sayou *et al*., (2016) found that monomers of the *Ginkgo biloba LFY* homologue that had their entire N‐terminal removed had significantly less DNA‐binding ability across the genome (in particular in sites of low‐binding affinity) when compared to the WT monomers. Thus, we hypothesize that removing highly conserved amino acids in the N‐terminal or removing the N‐terminal domain completely eliminated *ELFY’s* oligomerizing ability, thus rendering the flowers sterile. However, the remaining C‐terminal protein may had been able to weakly bind some of *ELFY’s* DNA targets inducing the creation of some underdeveloped reproductive‐like organs.

We examined gene expression upstream, near, and downstream of *ELFY* in the flowering pathway to help understand the floral developmental stage of the FT‐KO transgenic plants. Two patterns were seen in the gene expression analysis of floral genes. For *ELFY* and six genes upstream or at the same developmental stage as *ELFY* (i.e., *ECAL*, *EFT*, *EFUL1*, *EFUL2*, *ESPL3* and *ESPL9*; Figure 2c), expression was significantly higher in the FT‐KO transgenic events than in the FT‐control events (FT‐only‐control and FT‐Cas9‐control events). *EFT* is a floral pathway integrator (FPI) gene that induces the switch from vegetative to reproductive phase by binding to *ELFY* (Zhu *et al*., 2020). *ECAL*, *EFUL1,* and *EFUL2* are floral meristem identity (FMI) genes just as *ELFY*. We selected *EFUL1* and *EFUL2* because there is no archetypical *APETALA1* (*AP1*) homologue in *Eucalyptus* (Vining *et al*., 2015). *AP1* and *FUL* are homologous genes created from a gene duplication predating the diversification of the eudicots. *CAL* is also a homologue of *AP1* and *FUL* that is believed to have arisen from *AP1* during a more recent duplication. *AP1* and *FUL* are not functionally equivalent. They can only partially rescue each other in *Arabidopsis* (McCarthy *et al*., 2015). It is possible that one of the genes that has been identified as a *FUL* homologue (i.e., *EFUL1* or *EFUL2*) actually functions as an *AP1* homologue in *Eucalyptus*. However, we do not hypothesize which gene it could be because their expression is similar and because they both have the FUL‐like C‐terminal motif (i.e., LPAWML), which is missing in all the AP1 homologues (McCarthy *et al*., 2015).

The *SQUAMOSA PROMOTER BINDING PROTEIN‐LIKE* *(SPL)* genes are essential for induction of flowering. *SPL3/4/5* are only essential to the transition to flowering when they assist the FT‐FD complex in the activation of the FMI genes, *LFY*, *AP1,* and *FUL*, by directly binding to their promoter regions (Jung *et al*., 2016; Yamaguchi *et al*., 2009). Yamaguchi *et al*., (2014) hypothesized that *SPL9* recruits DELLA proteins to directly induce expression of *AP1* during transition from inflorescence meristem to flower meristem. During flowering, *LFY* activates many FOI genes including *APETALA1* (*AP1*), which then itself induces more *LFY* expression, generating a feed‐forward loop for controlling flowering (Gramzow and Theissen, 2010; Liu and Mara, 2010). With a non‐functioning ELFY, the feed‐forward loop cannot keep on cycling and increasing expression, causing flowering to be arrested in the inflorescence specification stage. Nonetheless, our FT‐KOs have high *FT* transgene expression, presumably by constitutively inducing high expression of the faulty *ELFY*.

Additionally, for five genes directly or indirectly regulated by *ELFY* (i.e., *EAP3*, *EPI*, *EAG*, *ESHP2* and *ESTK*; Figure 2d), expression was significantly lower in the FT‐KO transgenic events than in the controls. *EAP3*, *EPI*, *EAG*, *ESHP2,* and *ESTK* regulate expression of genes that make floral organs (reviewed in Pajoro *et al*., 2014). *EAP3* and *EPI* are B‐class genes, *EAG* is the C‐class gene, and *ESHP2* and *ESTK* are D‐class genes of the ABCDE model of flower development. This model has been thoroughly studied in *Arabidopsis*, petunia, snapdragon, and tomato (reviewed in Causier *et al*., 2010; Immink *et al*., 2010; Ó’Maoiléidigh *et al*., 2014; Pajoro *et al*., 2014; Rijpkema *et al*., 2010). *ELFY* directly regulates expression of *EAP3*, *EPI,* and *EAG*, and indirectly of *ESHP2* and *ESTK*.

In *Arabidopsis*, *AG* regulates the formation of stamens (with the B‐class genes, *AP3* and *PI*) and carpels, and its expression is essential for floral determinacy (Bowman *et al*., 1989; Mizukami *et al*., 1996; Yanofsky *et al*., 1990). Flowers become determinate when AG indirectly represses the stem cell maintenance gene *WUSCHEL* (*WUS*) (Liu *et al*., 2011; Sun *et al*., 2009). Our FT‐KO transgenic events had significantly lower expression of *EAG*, which may have been the reason for the repeated pedicel‐like and bract‐like structures, and thus the reduction in floral determinacy.

We briefly examined *ELFY’s* expression during early floral development. As expected from *Arabidopsis*, the controls started with relatively high transcript levels when the floral buds were just visually distinguishable (‘early bud’; Figure S7). The expression level then went down significantly with development; by the time the buds were about a month away from anthesis (‘late bud’), there was almost no detectable expression (Figure S7). In contrast, the expression of the two FT‐KO transgenic events did not show a clear downward trend, and never decreased to the very low level of ‘late buds’ that was seen in the FT‐controls. These results echo the high *ELFY* expression in the FT‐KOs reported in our gene profiling studies discussed above (Figure 2c). For reasons that are unclear, expression appears to have been reduced at the mid‐stage in FT‐KO transgenic event 30‐11, but not in FT‐KO transgenic 30‐10, though our limited sample precludes a firm conclusion about whether this difference is real or what its cause might be.

The *ELFY* expression of FT‐KO transgenic event 30‐11 was always significantly higher than the control event Cas9‐30‐14 or than FT‐KO transgenic event 30‐10. The FT‐KO transgenic event 30‐11 had the same mutation, a 4bp deletion, in both alleles. The peptide sequence for both alleles in this event is predicted to have four premature termination codons (PTCs) in 55 triplets, the first one showing up after 24 amino acids. Regardless of the presence of these PTCs, these transcripts do not appear to be targeted by nonsense‐mediated decay (NMD). The PTCs occur early in the amino acid sequence, and some studies suggest that PTCs too early or too late in the sequence are missed by NMD (Hori and Watanabe, 2007; Wu *et al*., 2007). The role of NMD has been studied in depth with respect to plant pathogen protection and plant immunity, but its full behaviour in plants remains incompletely understood (Jung *et al*., 2020).

It is possible that the expression of FT‐KO transgenic event 30‐11 was high both because NMD was not targeting it and because of the feedback between EFT‐EFD, ELFY, and EAP1. The inflorescences in *Eucalyptus* are determinate, unlike in *Arabidopsis;* thus, expression of *TFL1* is expected to disappear after vegetative meristems transition to inflorescence meristems. Our FT‐KO transgenic events may have been ‘stuck’ as inflorescences, thus they retained their high *EFT* and *ELFY* expression.

Because of the two general classes of gene expression we observed—where genes expressed upstream or at the same physiological level as *ELFY* had higher expression in FT‐KO transgenic events than in FT‐control events, and genes expressed downstream or regulated directly by *ELFY* had lower expression in FT‐KO transgenic events than in FT‐control events—it appears that the FT‐KO transgenic events were developmentally ‘trapped’ in inflorescence development (prior to floral organ development). The defective ELFY protein was stalling the process of flower development causing the constantly expressing FT‐KO transgenic events to not develop fertile flowers. Unfortunately, the high constitutive *FT* expression in our early‐flowering backgrounds complicates interpretation; and obtaining phenotypic data for KO *ELFY* mutant flowers in a WT background under natural conditions—which would require a number of years in the field in a subtropical environment—was beyond the scope of our study.

Interestingly, the expression level of *ELFY* at the late bud stage seems to correlate with the expression of *AtFT* and *EFT* at this same stage (Figure S8). It appears that overexpression of *AtFT* was inducing strong expression of the endogenous *FT*, *EFT*, and it was the sum of the transgene and endogene that best correlated with the strength of *ELFY* expression. It is not surprising that sum of the expression of the transgene and the endogene was well‐correlated with the expression of *ELFY* given that both FT and EFT were likely binding to the *ELFY* locus and inducing its expression, as seen recently in *Arabidopsis* (Zhu *et al*., 2020). The phenomenon where a transgene increases expression of the endogenous gene has not been widely reported, but is known from studies in both *Arabidopsis* and rice (Plett *et al*., 2010).

CRISPR Cas9 nucleases appear to provide an efficient method for elimination of *ELFY* function, and thus a means for preventing both male and female sexual reproduction without adverse vegetative impacts, when such containment is ecologically and economically prudent, socially acceptable, or mandated by law. This containment method is also expected to be highly stable over the long lifespans of trees in the field, especially when compared to previous methods for sterility induction such as the use of cytotoxins or gene suppression, whose efficacy can vary with environmental and developmental perturbations (Brunner *et al*., 2007; Vining *et al*., 2012). However, given the complex physiology and maturation processes of forest trees under field conditions, this conclusion is necessarily tentative. If sterility persists in the field under natural flowering, it may enable greater acceptance and faster regulatory approval of exotic or genetically engineered tree varieties, and thus speed the delivery of improved traits such as pest and disease resistance, modified wood properties, and biomass productivity that transgenic and gene‐edited trees appear to be capable of delivering (Chang *et al*., 2018).

## Materials and methods

### *Plant* *materials and CRISPR Cas9 construct production*

**Figure 5:**
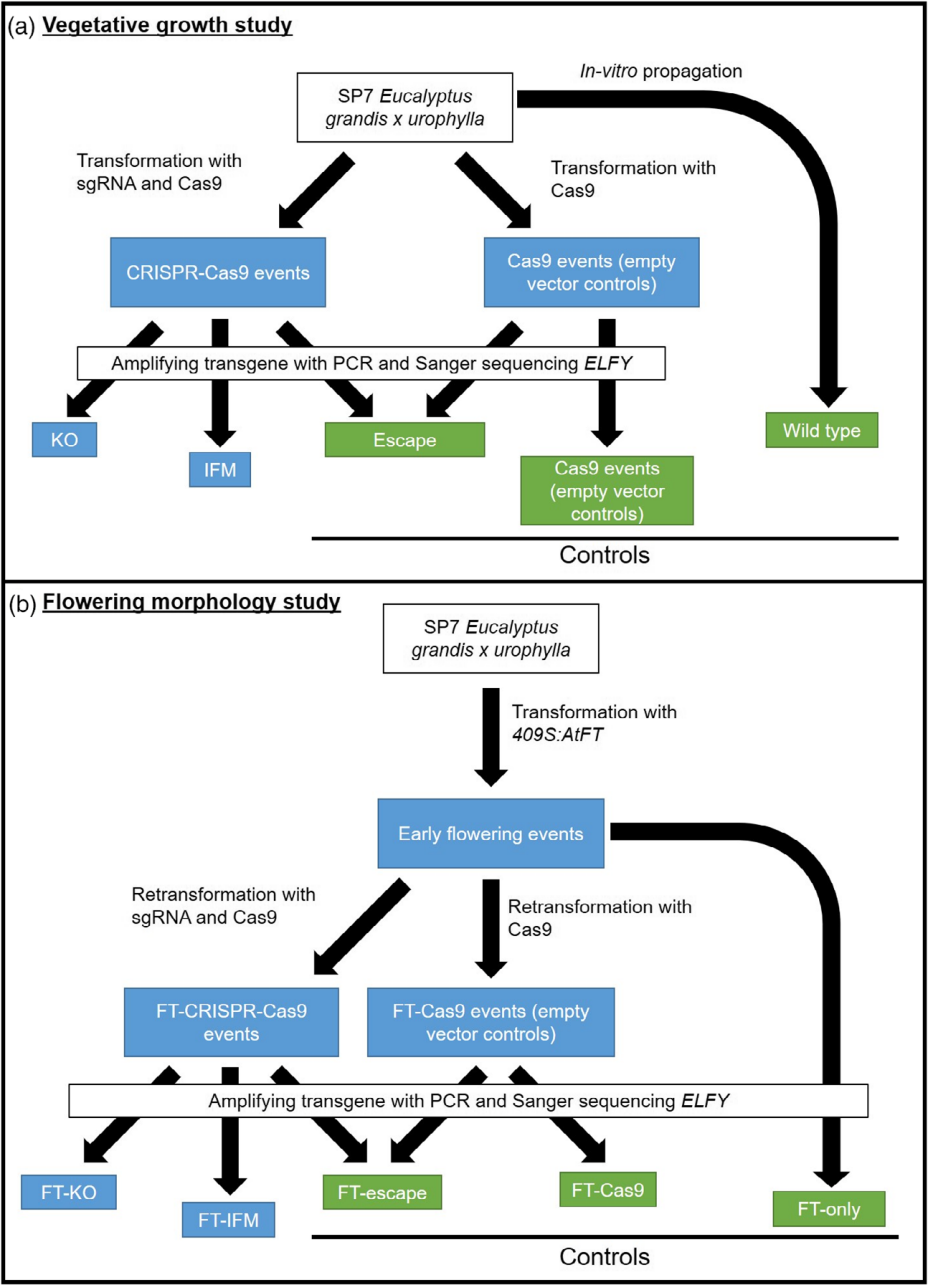
Overview of study design. (a) The ‘WT trial’ of vegetative growth in non‐flowering trees. (b) The ‘FT trial’ of floral morphology in early‐flowering trees. The original *Eucalyptus grandis* x *urophylla* hybrid clone is shown in white. The control groups (i.e., wild type, Cas9‐control events, and escape‐control events for WT trial, and FT‐only‐control events, FT‐Cas9‐control events, and FT‐escape‐control events for the FT trial) are shown in green boxes. The mutant events (i.e., KO and IFM for the WT trial, and FT‐KO and FT‐IFM for the FT trial) are shown in blue boxes. Cas9‐control and FT‐Cas9‐control transgenic lines do not contain sgRNAs. Escape, non‐transgenic but *Agrobacterium* cocultivated and regenerated lines. IFM, in‐frame mutant. KO, knockout based on sequence and phenotype. WT, wild type, not cocultivated or regenerated but micropropagated.

**Figure 6:**
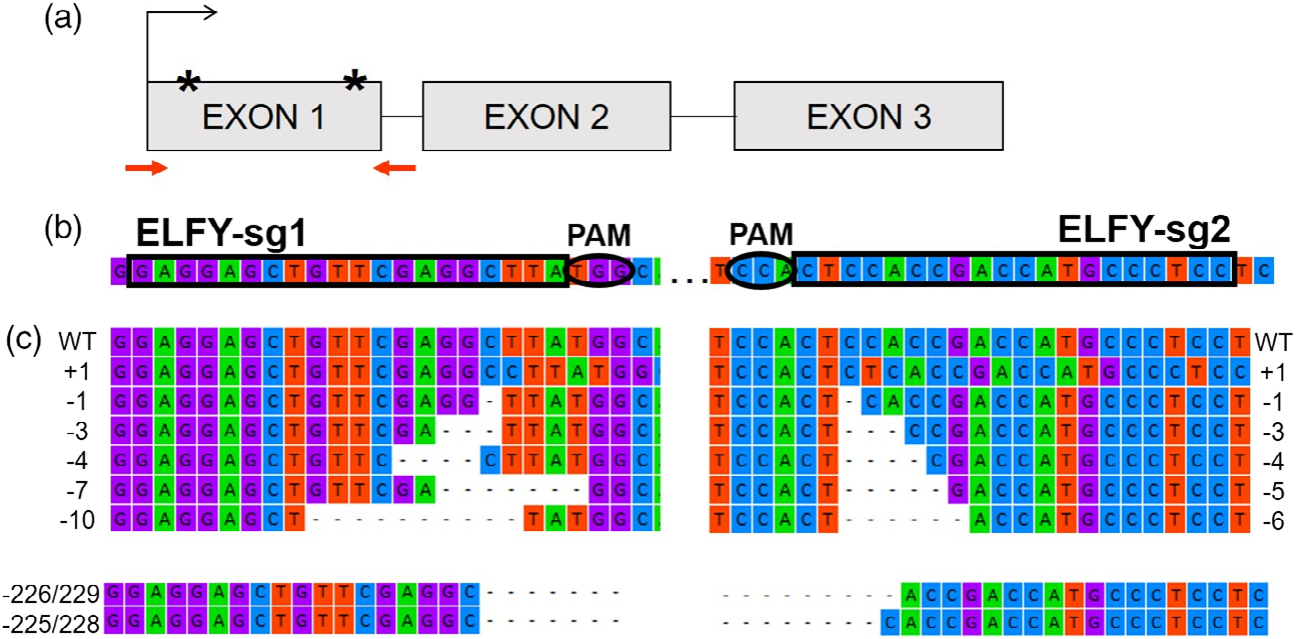
Summary of gene‐editing strategy in *ELFY*. (a) Schematic of the *ELFY* gene with the two sgRNA:*Cas9* targets (stars), target site one for ELFY‐sg1 and target site two for ELFY‐sg2. The sequencing primers are shown as red arrows. (b) Nucleotide sequence of the two target sites. The three periods correspond to the 213 bases (216 in *E. urophylla*) between target sites. The sequences matching the sgRNAs are surrounded by a black square and the protospacer adjacent motif (PAM) sequences by a black oval. (c) Common mutations seen among CRISPR‐Cas9 transgenic events modified with ELFY‐sg1 (top left), ELFY‐sg2 (top right) and ELFY‐sg1sg2 (bottom).

An overview of the plant materials produced and nomenclature is provided in Figure 5, and a glossary of terms used in Table S1. Sterile *in vitro* cultures of wild‐type (WT) hybrid *Eucalyptus* clone SP7 (*Eucalyptus grandis* × *urophylla*) were kindly provided by FuturaGene (http://www.futuragene.com/pt/). Two *AtFT* overexpressing lines (lines 4‐2 and 30‐3 transformed with pCAM:409S:AtFT under Hygromycin selection, FT‐4 and FT‐30 hereafter) were previously generated in our laboratory (Klocko *et al*., 2016b; Figure S1). We determined the first exon’s sequence of the *LFY* (AT5G61850) orthologue, *ELFY* (EUGRSUZ_K02192), in SP7 using TOPO^TM^ TA Cloning^TM^ (www.thermofisher.com) and the Sanger Sequencing service provided by the Center for Genome Research and Biocomputing (CGRB) at Oregon State University (cgrb.oregonstate.edu/core/sanger‐sequencing). We used the sgRNA design online tool ZiFit (Sander *et al*., 2007, 2010) to identify two different CRISPR Cas9 target sites in *ELFY* (Figure 6a,b, Figure S2). We created three CRISPR Cas9 constructs to target *ELFY* (i.e., two constructs with one sgRNA: ELFY‐sg1 and ELFY‐sg2, and one construct with the two sgRNAs: ELFY‐sg1sg2) and a single construct that lacked any sgRNAs as an empty vector control (i.e., a construct with only the Cas9 nuclease sequence). Constructs were assembled as in our previous study (Elorriaga *et al*., 2018) with some modifications (Methods S1).

### *Plant* *transformation, regeneration, and transgene genotyping*

The three CRISPR Cas9 constructs and the empty vector control construct were transformed into WT and FT SP7 using *Agrobacterium*‐mediated transformation methods (Methods S2; Chauhan *et al*., 2014). Genomic DNA from individual shoots was obtained according to Keb‐Llanes *et al*., (2002) and used for transgene confirmation and genotyping (primer details in Table S2).

### *Haplotype* *validation and allele‐specific PCR*

We identified natural allelic variants in both *ELFY* alleles using TOPO^TM^ TA Cloning^TM^ (www.thermofisher.com) following the manufacturer’s instructions. We genotyped each event’s alleles using allele‐specific PCR (Cha *et al*., 1992; Newton *et al*., 1989) (primer details in Table S2, Figure S2). We used three single nucleotide polymorphisms (SNPs, located at positions 12, 328, and 335 from the translational start site in the *E. grandis* allele; Figure S2) to design allele‐specific primers. Amplicons were sequenced using the Sanger Sequencing service provided by the CGRB (Methods S2). Sequences were aligned and translated using MEGA6 (Tamura *et al*., 2013).

### *Rooting* *and greenhouse conditions*

We selected events with predicted knockout mutations in both *ELFY* alleles. We propagated these events to generate multiple identical ramets (trees). Individual rooted ramets were transferred to soil in two‐inch square pots to acclimate to *ex vitro* conditions. After a month of acclimation in a humid glasshouse, we moved the ramets to a greenhouse and transplanted each one to an eight‐inch circular pot. All the transgenic events were randomized in one block with non‐transgenic WT SP7 control ramets that were grown and propagated in tissue culture.

### *Vegetative* *data measurements and statistical analyses*

We recorded two growth‐related traits: stem height and diameter, and four leaf‐related traits: relative chlorophyll density (using a Soil Plant Analysis Development, SPAD‐502, meter), leaf area, leaf perimeter, and leaf weight. From these, we derived stem volume index and specific leaf weight. The details on the measurements and the statistical analyses can be found in the supplementary methods (Methods S2).

### *Analyses* *of floral morphology in the* *FT‐CRISPR trial*

Flowering was first recorded after the ramets were randomized in the greenhouse. Some ramets had flowered with a few buds already while acclimating in the glasshouse. Flower morphology was recorded every month for twelve months. Flower buds and flowers were imaged whole and dissected longitudinally using a Keyence VHX‐1000 digital microscope. Buds and flowers, from early to late developmental stages, were dissected to determine if any developing or underdeveloped reproductive organs were present.

### *RNA* *isolation and cDNA synthesis*

We collected flower buds after they had just shed their calycine operculum (˜week 8 of bud development) in the early afternoon of 4 October 2018. We sampled buds from six FT‐KO events: 30‐2, 30‐10, 30‐11, 30‐31, 30‐40 and 30‐45. We also sampled buds from two FT‐Cas9‐control events, Cas9‐30‐14 and Cas9‐30‐5, and from two ramets of the FT‐only‐control FT‐30. We also collected flowers buds at the earliest recognizable stage (i.e., week 0 of bud development) and during bract shedding (˜week 4 of bud development) in the early afternoon of 23 March 2020. The samples earlier in development were from FT‐Cas9‐control event Cas9‐30‐14 and FT‐KO events 30‐10 and 30‐11. Two to three buds were collected from two ramets (approximately one gram of tissue in total) of the same event and mixed together for RNA isolation. The buds were sampled, frozen immediately in liquid N_2_, and kept at −80 °C until RNA isolation. RNA was extracted according to Howe *et al*., (2013). The RNA samples were treated with DNaseI (New England Biolabs, Ipswich, MA, USA) and submitted to the CGRB for analysis by the Agilent Bioanalyzer 2100 to determine their integrity. The SuperScript III First‐Strand Synthesis system (Invitrogen) was used to synthesize cDNA from the DNase‐treated RNAs.

### *Gene* *expression and statistical analysis*

Real‐time quantitative PCR (qPCR) analysis was performed in a StepOnePlus Real‐Time PCR system (Applied Biosystems). We recorded the expression of *ELFY* and other genes in the flower development pathway (Bouché *et al*., 2016; Smaczniak *et al*., 2012; Theißen *et al*., 2016; Wils and Kaufmann, 2017) that were upstream, downstream, or at the same developmental stage as *LFY* in *Arabidopsis* (Table S2, Methods S2). The details of the experimental design are in the supplementary methods (Methods S2, Table S4). The DataAssist v3.01 software (Applied Biosystems) conducted a two‐sample two‐tailed Student’s t‐test to determine if expression of the FT‐control‐events was different from that of the FT‐KO transgenic events for each gene.

## Author contributions

EE, SHS, and ALK designed the study. EE also sequenced the target gene, designed and constructed the vectors, gathered, analysed and interpreted the data, and wrote the manuscript. CM performed the plant stable transformation, regeneration and selection, and transplanting. ALK helped with vector construction. MP created Figure S1. XA sequenced several independent transgenic events. AAM supervised the work at University of Pretoria. SHS with help from ALK obtained funding for the study and supervised the overall study. The manuscript was read and approved by all the authors.

## Conflict of interest

The authors declare no conflicts of interest.

## Supporting information


**Video S1** X‐ray projection of FT‐only‐control inflorescence.

**Video S2** X‐ray projection of FT‐KO transgenic inflorescence.

**Figure S1** Images of flowers produced in FT‐only‐control FT‐30
**Figure S2** DNA sequence alignment of the two alleles of ELFY in the hybrid Eucalyptus clone SP7
**Figure S3** Allelic chromatograms of FT‐events in gene expression experiments
**Figure S4** Partial peptide alignment of the N‐terminal in WT and some mutants
**Figure S5** Developmental sequence of flower formation in the greenhouse
**Figure S6** Sterile floral‐like buds seen in different FT‐KO events
**Figure S7** Flower buds and flowers of FT‐only and FT‐KO events in a greenhouse trial at the University of Pretoria in South Africa
**Figure S8** *ELFY* gene expression from qPCR at three different bud development stages
**Figure S9** Scattergram of the relative expression of *ELFY* across the relative expression of *AtFT* and/or *EFT*
**Figure S10** 3D representation of X‐ray projections of inflorescences
**Figure S11** Underdeveloped organs appeared occasionally in six‐month‐old or older buds from FT‐KO events
**Figure S12** Peptide alignment of the N‐terminal domain in *LFY* and orthologous transcription factors
**Figure S13** Peptide alignment of the N‐terminal domain of the FT‐IFM events
**Figure S14** Leaf phenotypes of potted plants in WT trial
**Figure S15** Leaf phenotypes of potted plants in FT trial
**Figure S16** Leaf phenotypes of FT‐IFM and FT‐control events
**Table S1** Glossary.
**Table S2** Primers.
**Table S3** Transformation efficiency rates.
**Table S4** Gene names and IDs for qPCR experiments.
**Table S5** Predicted knock‐out (i.e., loss‐of‐function) rates based on the occurrence of frameshifts, large deletions (i.e. ≥222 bp), and deletions of highly conserved amino acids.
**Table S6** Inventory of plants in the greenhouse trials.
**Table S7** Phenotypes seen in FT‐KO events kept in the greenhouse.
**Methods S1** Target Sequence cloning protocol.
**Methods S2** Additional methods.
