# Supplementary material for: Genetic containment in vegetatively propagated forest trees: CRISPR disruption of LEAFY function in Eucalyptus gives sterile indeterminate inflorescences and normal juvenile development
Source: Plant Biotechnol J. 2021 May 4;19(9):1743–55. doi: 10.1111/pbi.13588 (PMC8428835; doi:10.1111/pbi.13588)
Supplement: Supplementary file 3 — Figure S1 Images of flowers produced in FT‐only‐control FT‐30 Figure S2 DNA sequence alignment of the two alleles of ELFY in the hybrid Eucalyptus clone SP7 Figure S3 Allelic chromatograms of FT‐events in gene expression experiments Figure S4 Partial peptide alignment of the N‐terminal in WT and some mutants Figure S5 Developmental sequence of flower formation in the greenhouse Figure S6 Sterile floral‐like buds seen in different FT‐KO events Figure S7 Flower buds and flowers of FT‐only and FT‐KO events in a greenhouse trial at the University of Pretoria in South Africa Figure S8 ELFY gene expression from qPCR at three different bud development stages Figure S9 Scattergram of the relative expression of ELFY across the relative expression of AtFT and/or EFT Figure S10 3D representation of X‐ray projections of inflorescences Figure S11 Underdeveloped organs appeared occasionally in six‐month‐old or older buds from FT‐KO events Figure S12 Peptide alignment of the N‐terminal domain in LFY and orthologous transcription factors Figure S13 Peptide alignment of the N‐terminal domain of the FT‐IFM events Figure S14 Leaf phenotypes of potted plants in WT trial Figure S15 Leaf phenotypes of potted plants in FT trial Figure S16 Leaf phenotypes of FT‐IFM and FT‐control events Table S1 Glossary. Table S2 Primers. Table S3 Transformation efficiency rates. Table S4 Gene names and IDs for qPCR experiments. Table S5 Predicted knock‐out (i.e., loss‐of‐function) rates based on the occurrence of frameshifts, large deletions (i.e. ≥222 bp), and deletions of highly conserved amino acids. Table S6 Inventory of plants in the greenhouse trials. Table S7 Phenotypes seen in FT‐KO events kept in the greenhouse. Methods S1 Target Sequence cloning protocol. Methods S2 Additional methods. [file PBI-19-1743-s003.pdf]

Supplemental Information – Elorriaga et al. 2020

Fig. S1 Images of *E. grandis* flowers produced in transgenic overexpression event (FT-only-control event 30-3).

Fig. S2 DNA sequence alignment of the two alleles of *ELFY* in the hybrid *Eucalyptus* clone SP7.

Fig. S3 Partial peptide alignment of the N-terminal in WT and some mutants.

Fig. S4 Developmental sequence of flower formation in the greenhouse.

Fig. S5 Sterile floral-like buds seen in different FT KO events.

Fig. S6 Flower buds and flowers of FT-only and FT KO events in a greenhouse trial at the University of Pretoria in South Africa.

Fig. S7 *ELFY* gene expression from qPCR at three different bud development stages.

Fig. S8 Scattergram of the relative expression of *ELFY* across the relative expression of *AtFT* and/or *EFT*.

Fig. S9 3D representation of X-ray projections of inflorescences.

Fig. S10 Underdeveloped organs appeared occasionally in six-month-old or older buds from *FT* KO events.

Fig. S11 Peptide alignment of the N-terminal domain in *LFY* and orthologous transcription factors.

Fig. S12 Peptide alignment of the N-terminal domain.

Fig. S13 Leaf phenotypes of potted plants in WT trial.

Fig. S14 Leaf phenotypes of potted plants in FT trial.

Fig. S15 Leaf phenotypes of IFM and control events in FT trial.

Table S1 Glossary.

Table S2 Primers table.

Table S3 Gene names and IDs for qPCR experiments.

Table S4 Predicted knock-out (i.e., loss-of-function) rates based on the occurrence of

frameshifts, large deletions (i.e. >=222 bp), and deletions of highly conserved amino acids.

Table S5 Inventory of plants in the greenhouse trials.

Table S6 Phenotypes seen in FT KO events kept in the greenhouse.

Methods S1 Target Sequence cloning Protocol.

Methods S2 Eucalypt transformation and regeneration in brief.

Video/Movie S1 X-ray projection of FT-only inflorescence.

Video/Movie S2 X-ray projection of FT KO inflorescence.

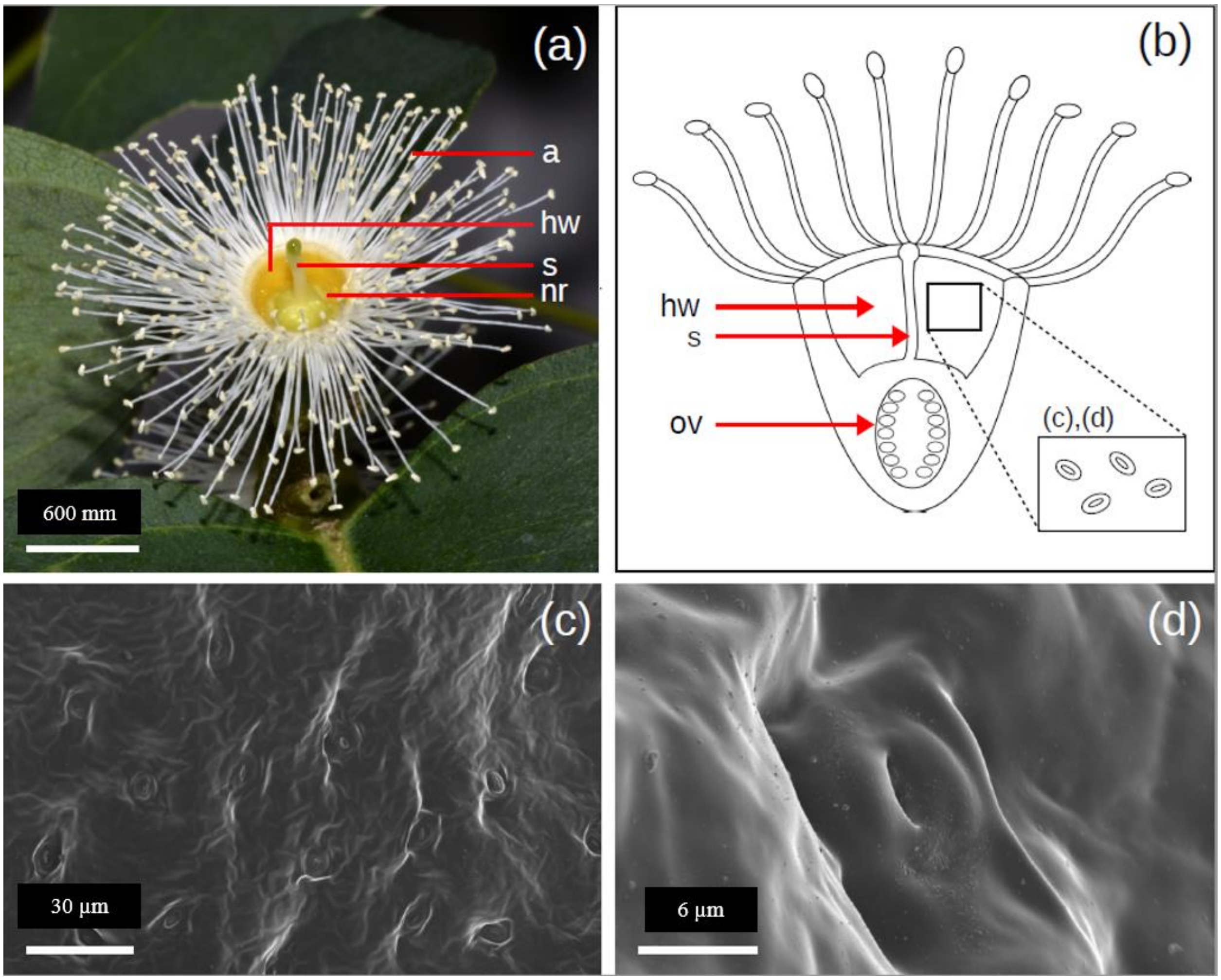

Fig. S1 Images of flowers produced in FT-only-control FT-30. (**a**) Opened flower showing anthers (a), style (s), hypanthium wall (hw), and nectary ring (nr). (**b**) Diagram of flower showing style (s), ovary (ov), and hypanthium wall (hw), as well as the position of the images shown in panels (c) and (d). (**c**) and (**d**) Scanning electron microscopy (SEM) micrographs of hypanthium wall (hw) showing nectary pores at 1,150X and 7,480X magnification, respectively.

```
>>>E.grandis, 439 nt vs E.uro, 442 nt
Waterman-Eggert score: 2156;  165.9 bits; E(1) <  2.2e-45
98.6% identity (98.6% similar) in 442 nt overlap (1-439:1-442)

                  10        20        30      * 40        50        60
E.gran  GCTGAAGCCATTATGCAGCGCGAGAATGGATCCAGAAGCATTTGCGGTTGTGGGGTTGCG
        :::::::::::::::::::::::::::::::::::: :::::::::::::::::::::::
E.uro   GCTGAAGCCATTATGCAGCGCGAGAATGGATCCAGAGGCATTTGCGGTTGTGGGGTTGCG
                  10        20        30        40        50        60

                  70        80        90       100       110       120
E.gran  AACGATGGGGGGACTGGAGGAGCTGTTCGAGGCTTATGGCATAAGGTACCTCACGGCCTC
        ::::::::::::::::::::::::::::::::::::::::::::::::::::::::::::
E.uro   AACGATGGGGGGACTGGAGGAGCTGTTCGAGGCTTATGGCATAAGGTACCTCACGGCCTC
                  70        80        90       100       110       120

                 130       140       150       160       170       180
E.gran  CAGGATAGCGGAAATGGGGTTTACGGCCAACACCCTCCTCGACATGAAGGAGGAGGAGCT
        ::::::::::::::::::::::::::::::::::::::::::::::::::::::::::::
E.uro   CAGGATAGCGGAAATGGGGTTTACGGCCAACACCCTCCTCGACATGAAGGAGGAGGAGCT
                 130       140       150       160       170       180

                 190       200       210       220       230       240
E.gran  CGACGACATGATGAACTCCCTCTCCCACATCTTCCGCTGGGACCTCCTCGTCGGCGAGCG
        ::::::::::::::::::::::::::::::::::::::::::::::::::::::::::::
E.uro   CGACGACATGATGAACTCCCTCTCCCACATCTTCCGCTGGGACCTCCTCGTCGGCGAGCG
                 190       200       210       220       230       240

                 250       260       270       280       290
E.gran  CTACGGCATCAAGGCCGCCATCCGCGCCGAGCGCCGACGCCTCCTCGAAGCCGATGACC-
        ::::::::::::::::::::::::::::::::::::::::::::::::::::::::::: 
E.uro   CTACGGCATCAAGGCCGCCATCCGCGCCGAGCGCCGACGCCTCCTCGAAGCCGATGACCA
                 250       260       270       280       290       300

        300       310       320       330       340       350 *
E.gran  --GCCGCCACCACCTCCACTCCACCGACCATGCCCTCCTCGATGCTCTCTCCCACAAAGG
          ::::::::::::::::::::::::::::::::::::::::::::::::::::: ::::
E.uro   CCGCCGCCACCACCTCCACTCCACCGACCATGCCCTCCTCGATGCTCTCTCCCACCAAGG
                 310       320       330       340       350       360

          *
        360       370       380       390       400       410
E.gran  TAACTTAACTCGGCCTCTTTGATTTTTGACGTTTTGGGTCATTATGATTATTCCCACGCA
        :: :::::::::::::::::::::::::::::::::::::::::::::::::::::::::
E.uro   TACCTTAACTCGGCCTCTTTGATTTTTGACGTTTTGGGTCATTATGATTATTCCCACGCA
                 370       380       390       400       410       420

        420       430
E.gran  GGGCTGTCGGAGGAACAAGTGA
        ::::::::::::::::::::::
E.uro   GGGCTGTCGGAGGAACAAGTGA
                 430       440
```

Legend

Egrandis_F3
Euro_F3
Egrandis_R1
Euro_R1
Target 1
Target 2
Protospacer adjacent motif (PAM)
ATG: translational start site
*: SNP

Fig. S2 DNA sequence alignment of the two alleles of *ELFY* in the hybrid *Eucalyptus* clone SP7. The top allele is from the *Eucalyptus grandis* parent and the bottom allele from *E. urophylla* parent. Colored boxes show the location of the allele-specific primers. The first and second CRISPR Cas9 target sites are highlighted in yellow and teal, respectively. And the protospacer adjacent motif (PAM) found directly after both target sites are highlighted in fuchsia. The translational start site is in bold and a one size larger font. Single nucleotide polymorphisms have an asterisk (*) above the *E. grandis* nucleotide.

FT-only-control

a) FT-30

E. grandis allele (site one)

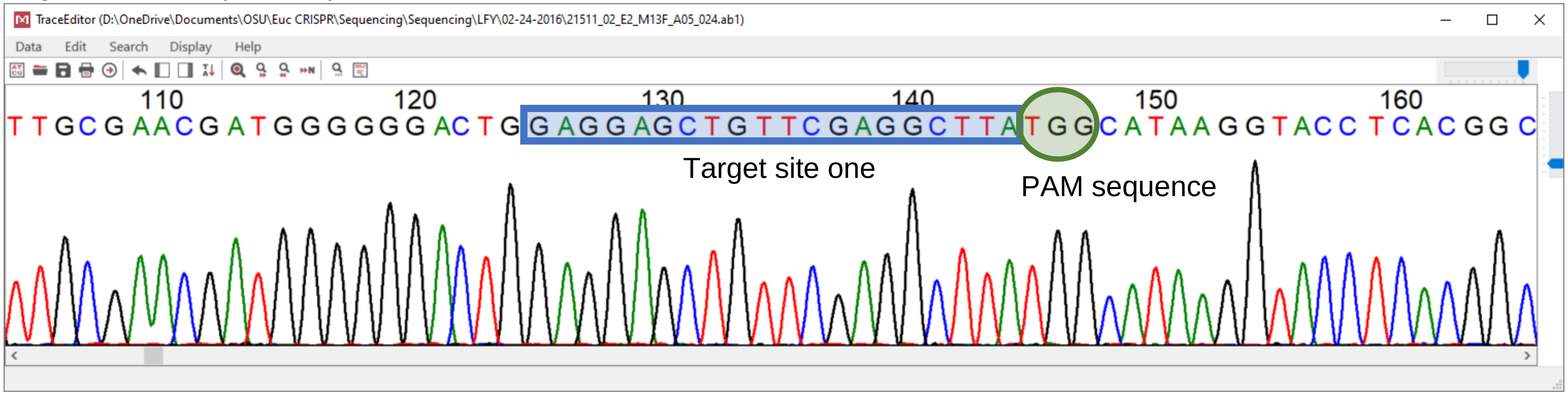

E. grandis allele (site two)

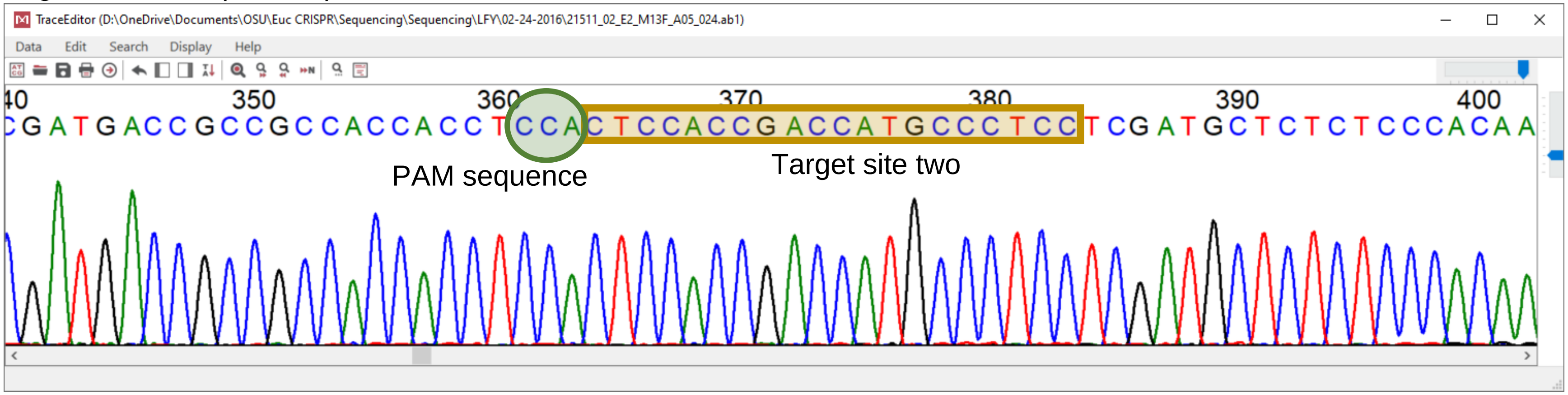

E. urophylla allele (site one)

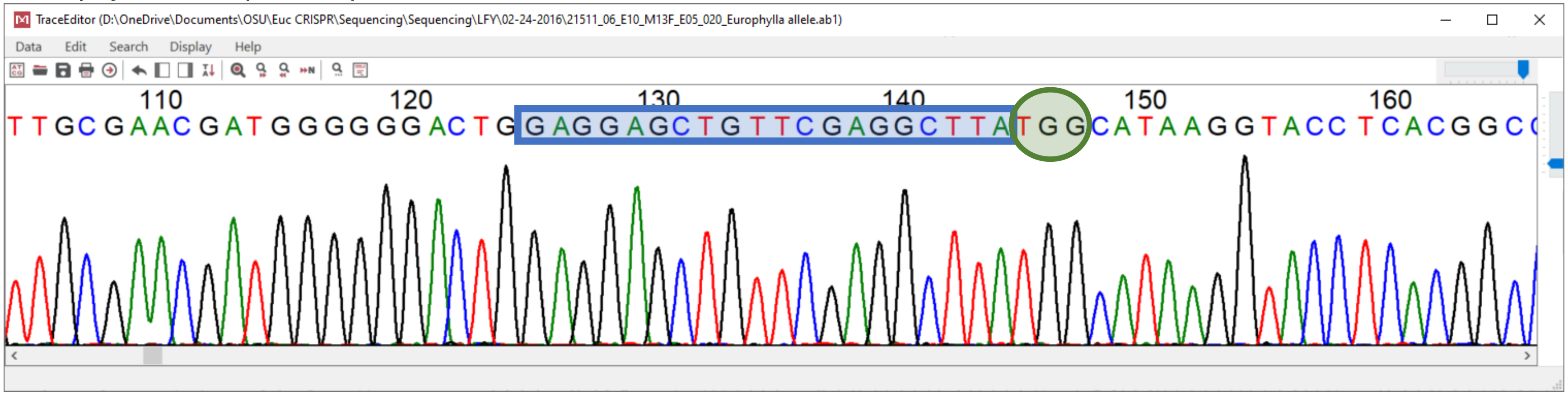

E. urophylla allele (site two)

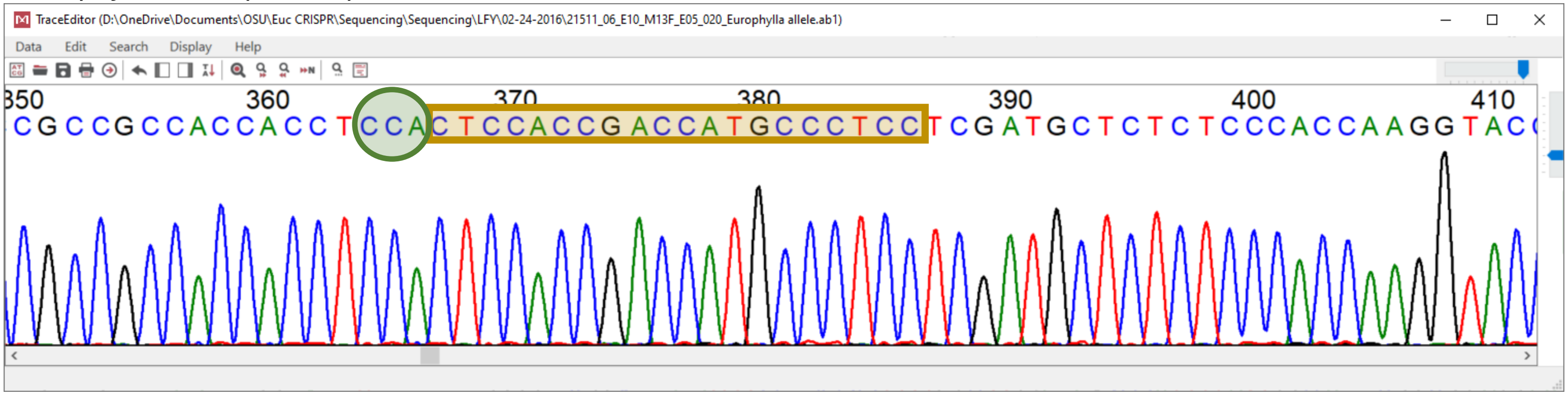

FT-Cas9-controls

b) Cas9-30-14

E. grandis allele (site one)

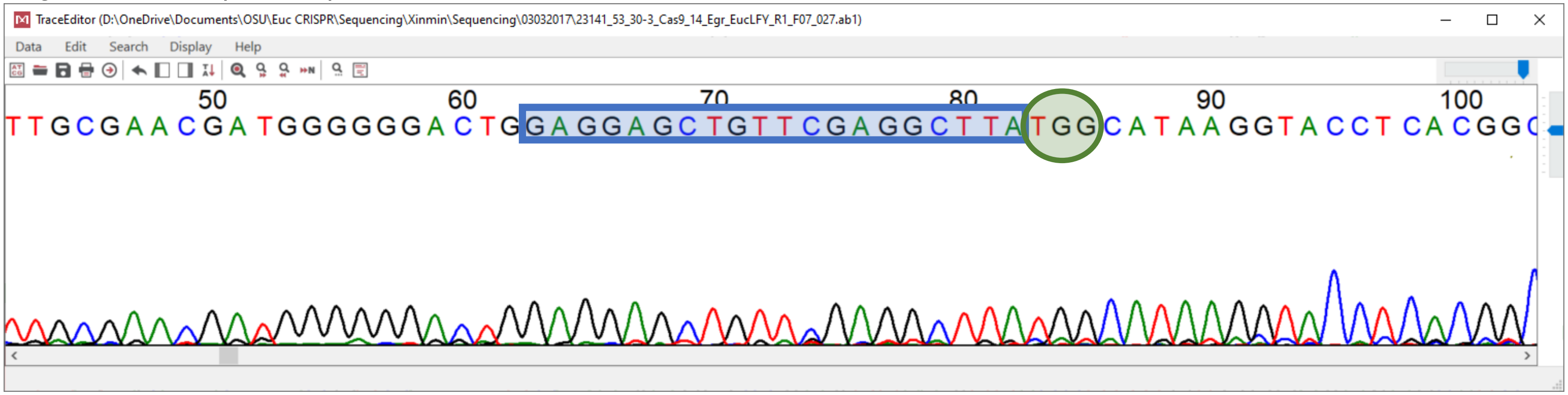

E. grandis allele (site two)

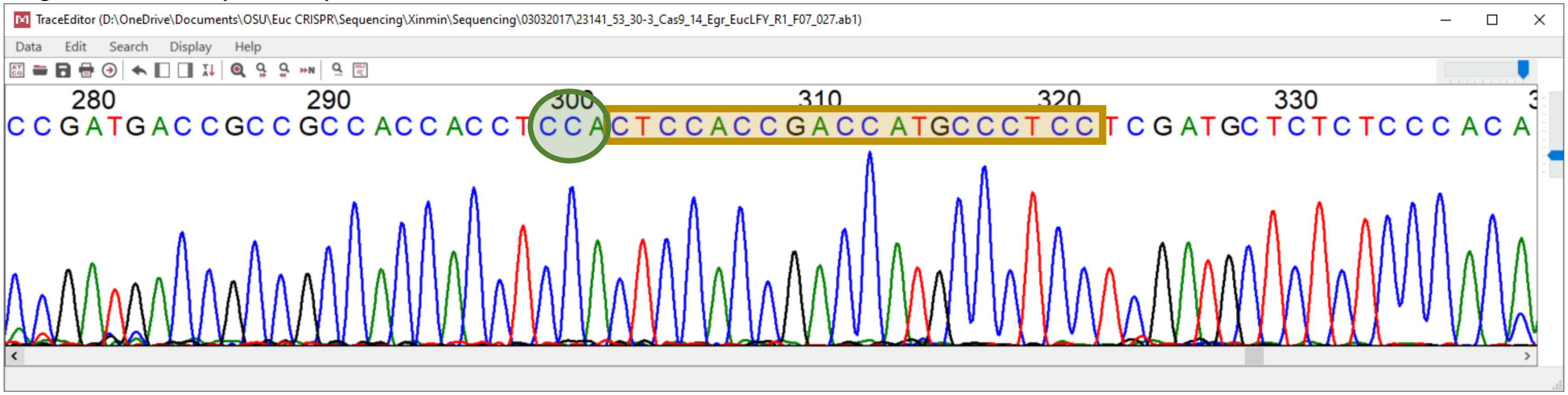

E. urophylla allele (site one)

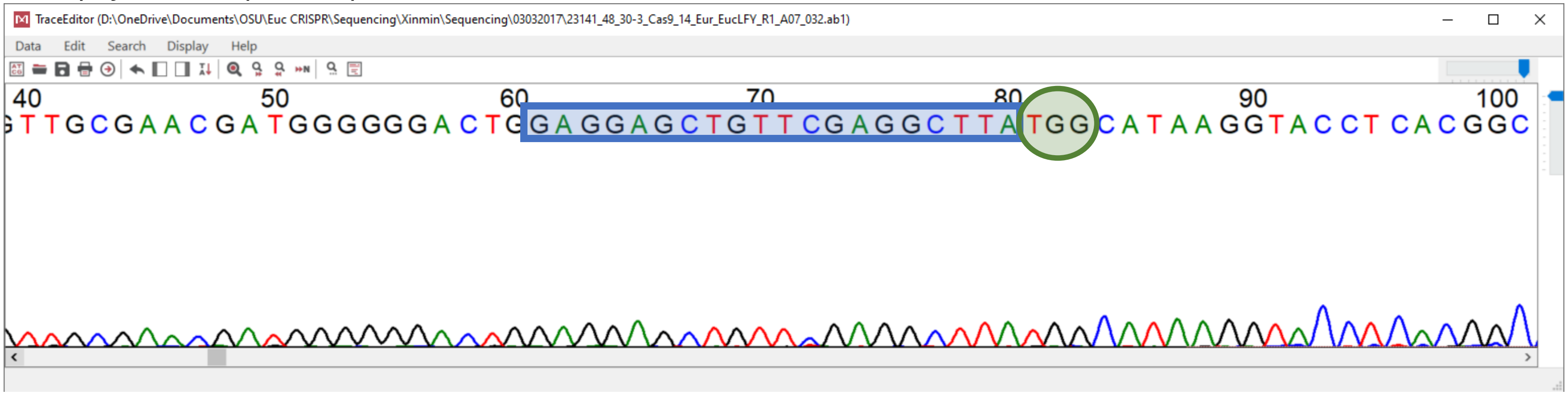

E. urophylla allele (site two)

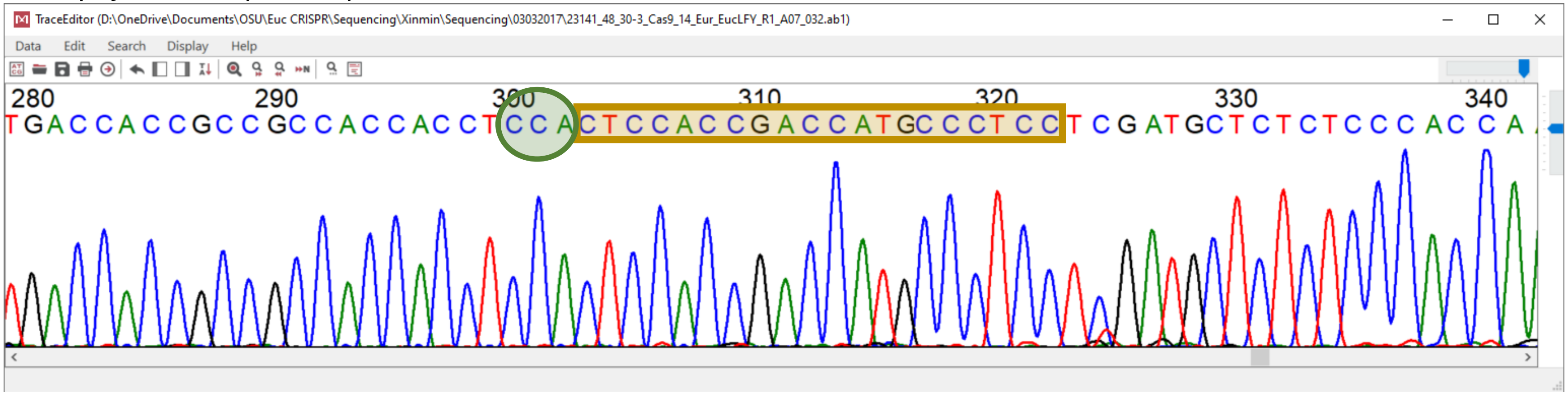

FT-KOs

Events with ELFY-sg1

c) FT-KO 30-10:

E. grandis allele

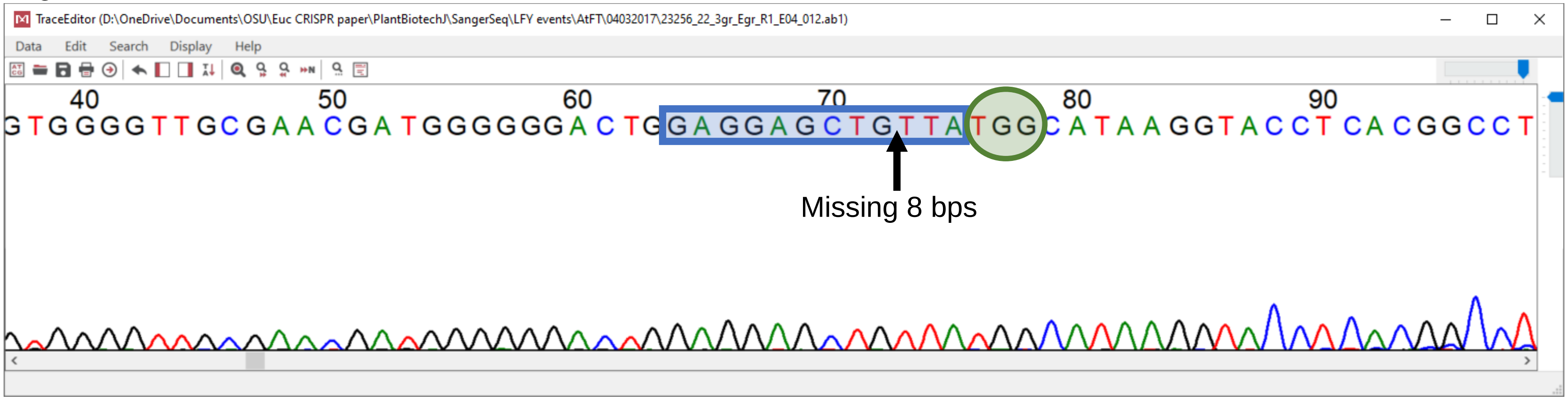

E. urophylla allele

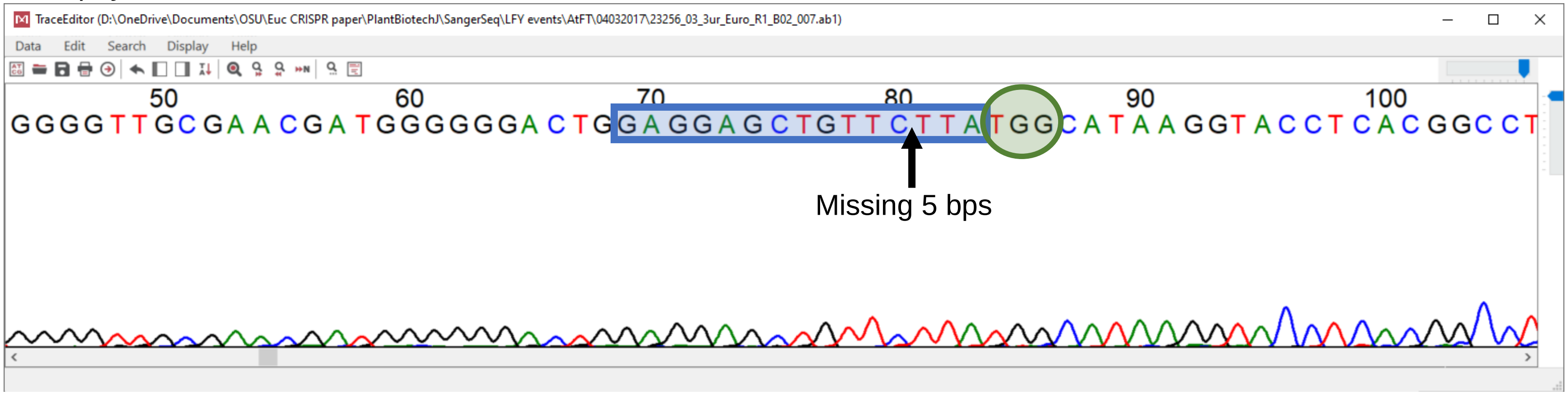

d) FT-KO 30-11:

E. grandis allele

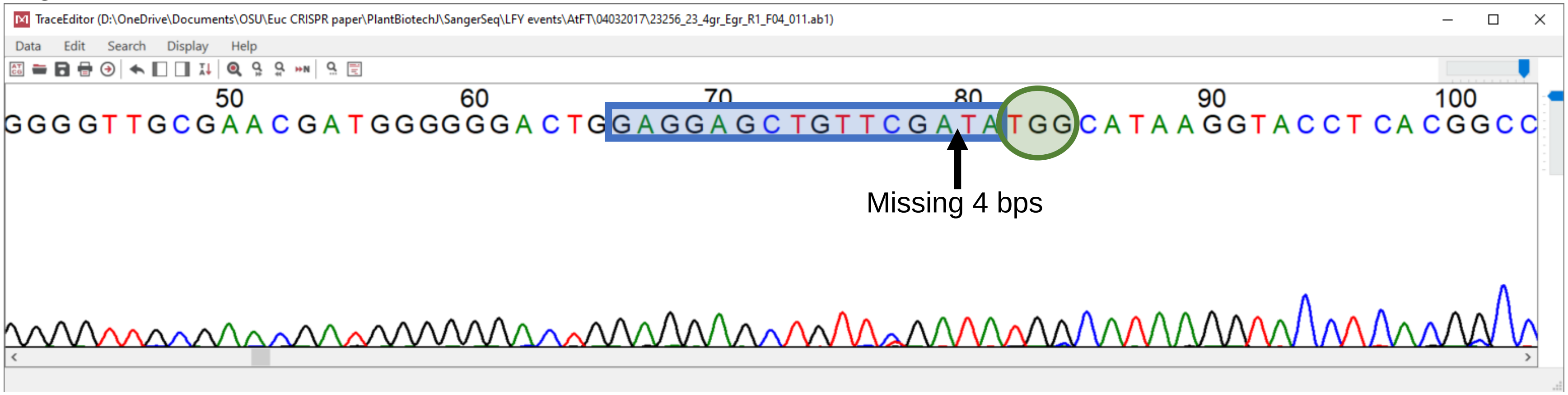

E. urophylla allele

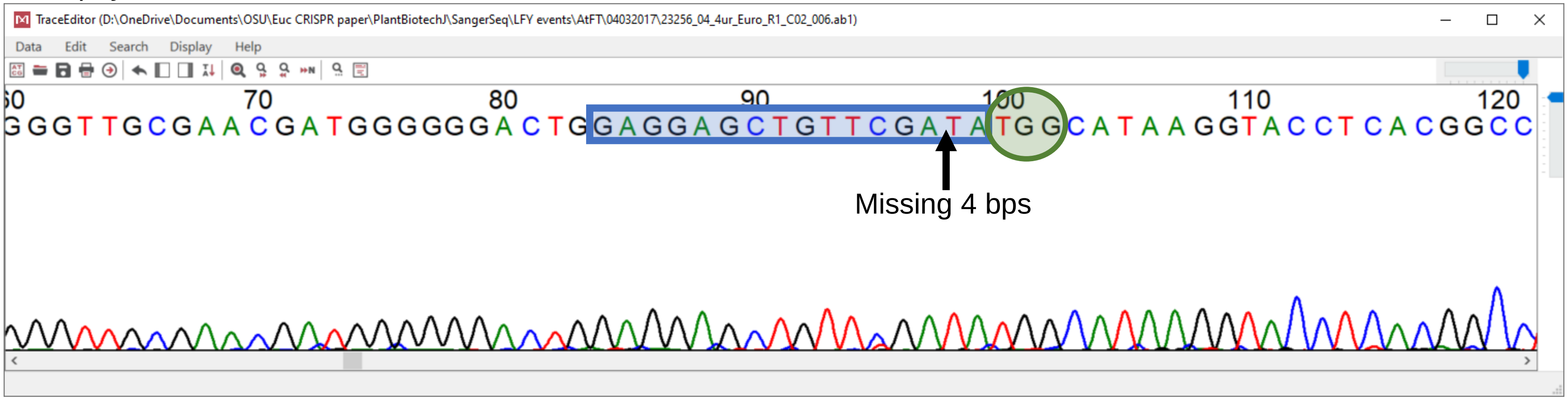

Events with ELFY-sg2

e) FT-KO 30-31:

E. grandis allele

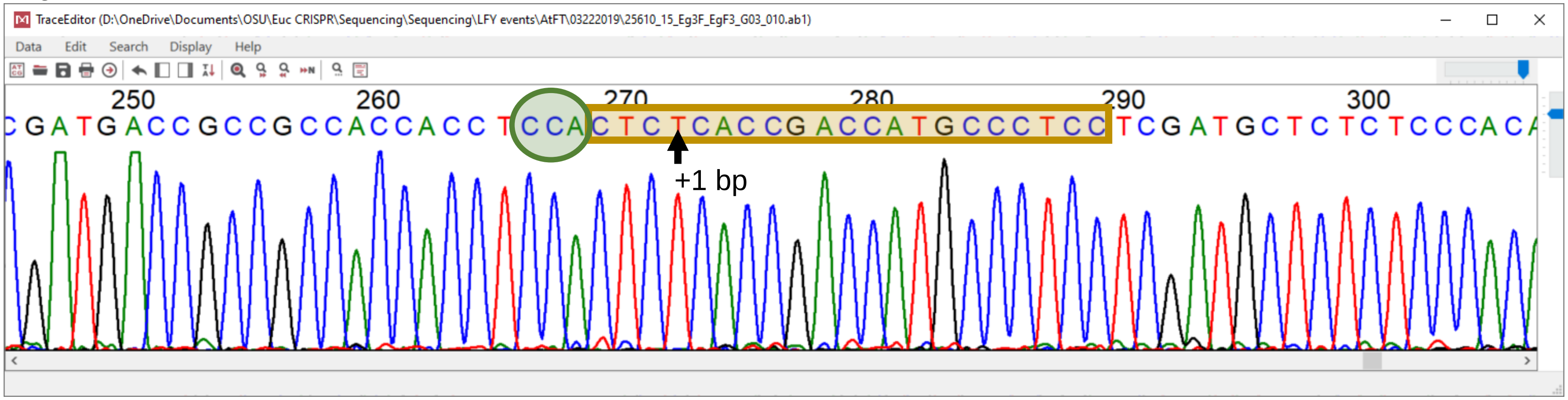

E. urophylla allele (and the *E. grandis* allele): This reaction amplified both alleles with the *E. urophylla*-specific primers. The double peaks happen right on the site of the mutation. By removing the peaks corresponding to the *E. grandis* allele (see above), we determined that this allele had a 23bp deletion.

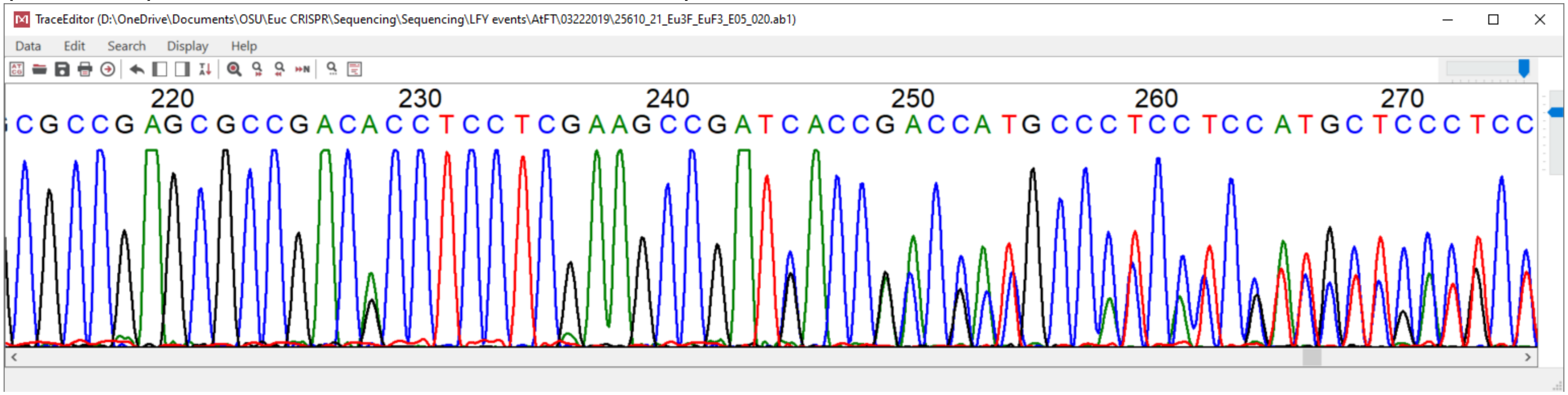

f) FT-KO 30-45:

E. grandis allele

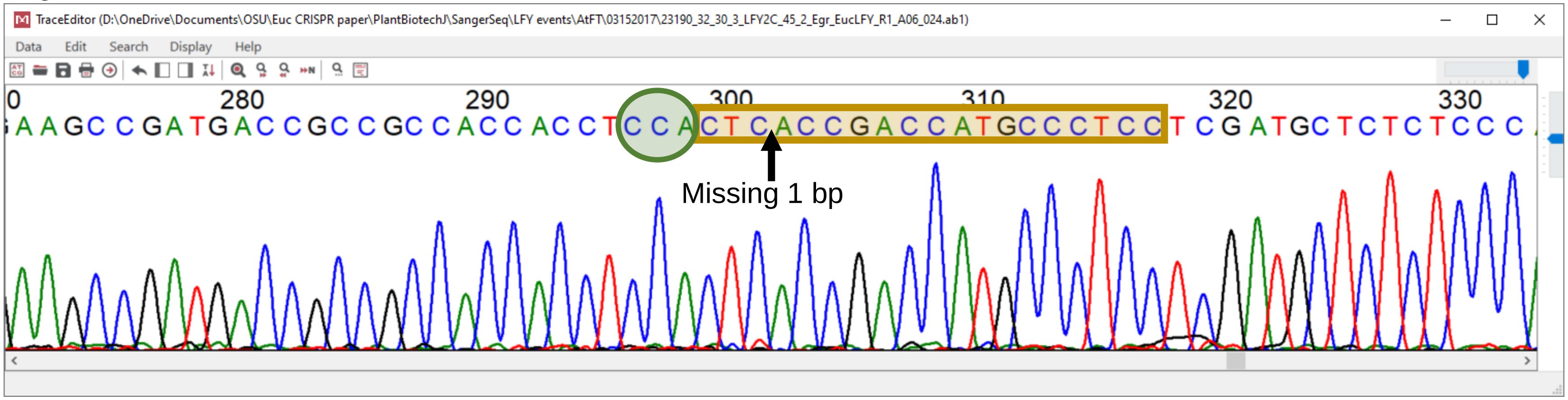

E. urophylla allele

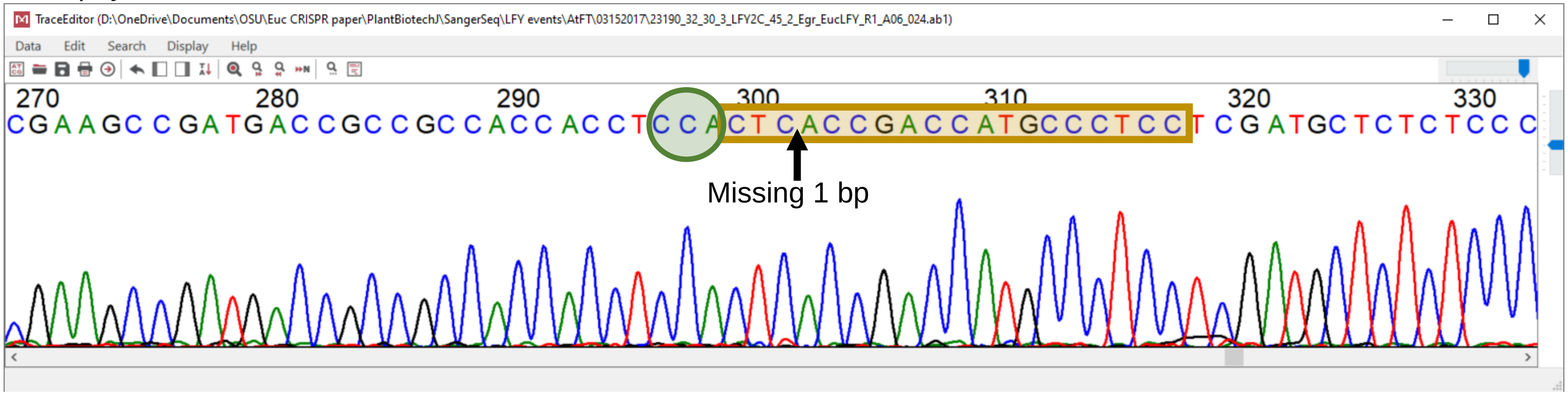

Events with ELFY-sg1sg2

g) FT-KO 30-2:

E. grandis allele (site one)

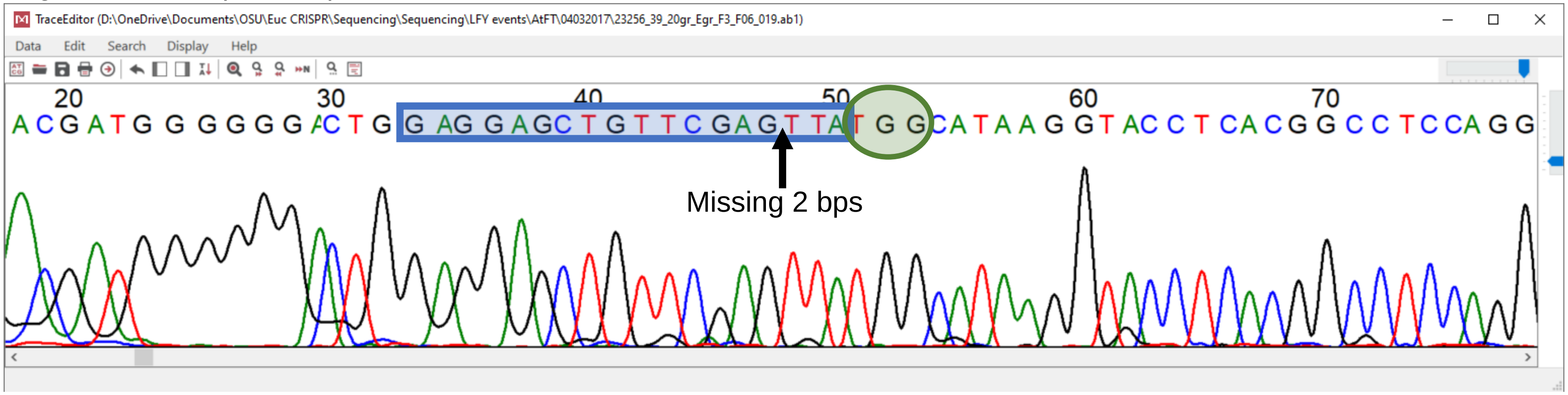

E. grandis allele (site two)

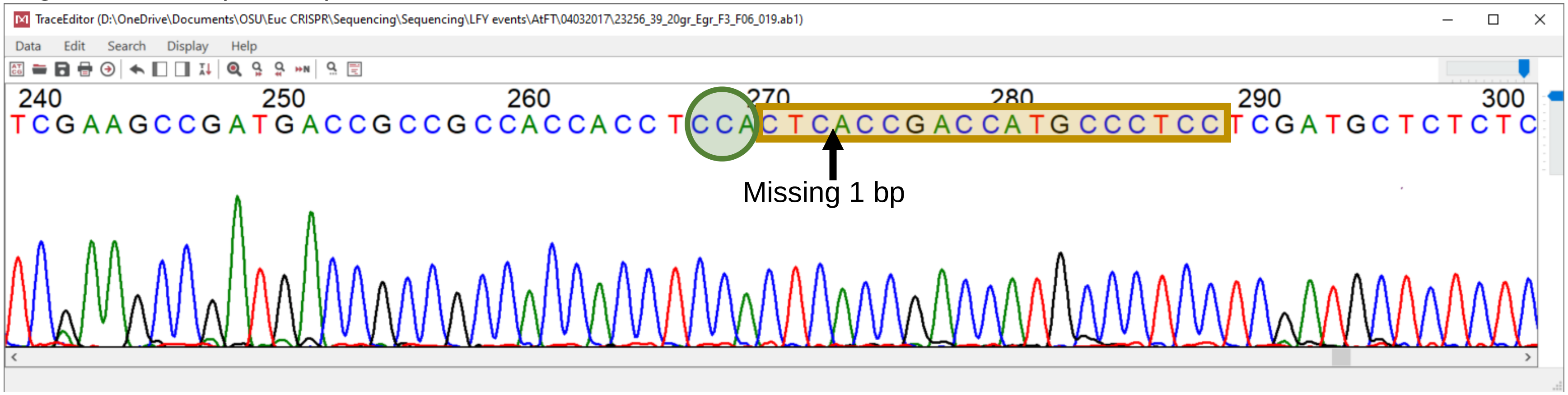

E. urophylla allele (site one)

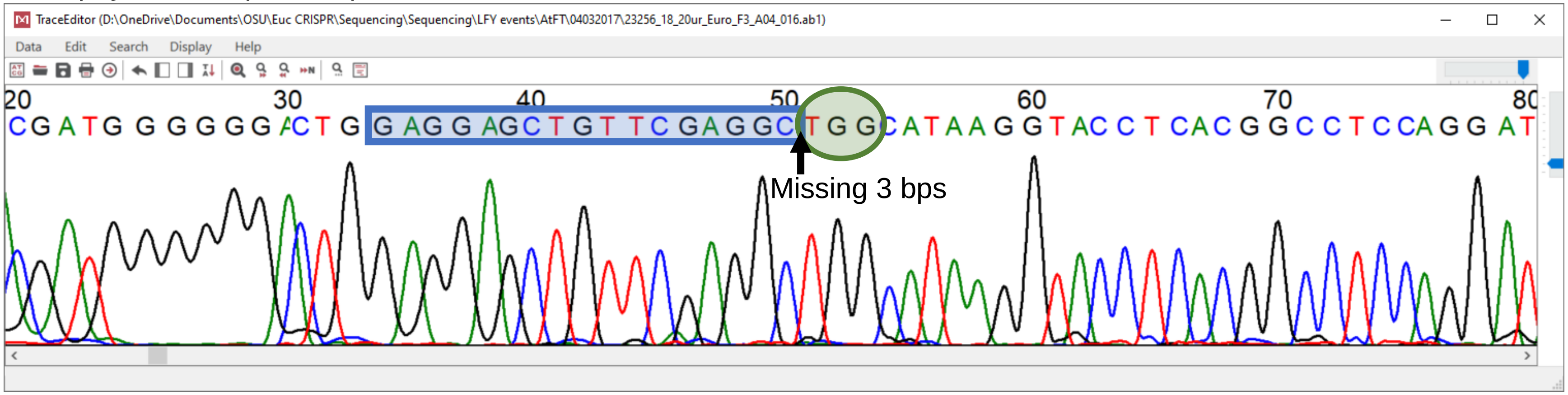

E. urophylla allele (site two)

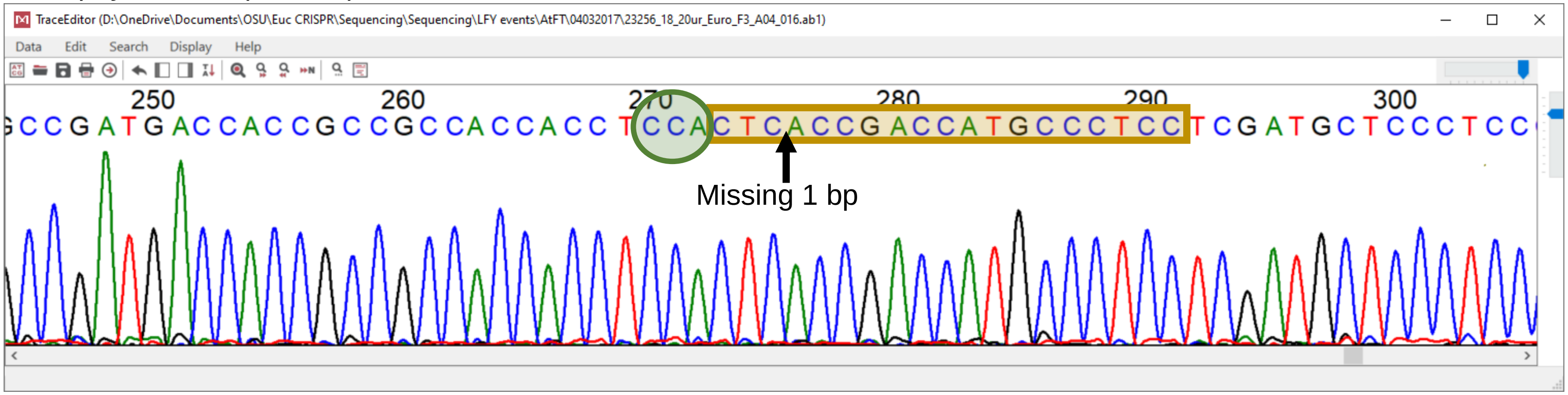

h) FT-KO 30-40:

E. grandis allele (site one)

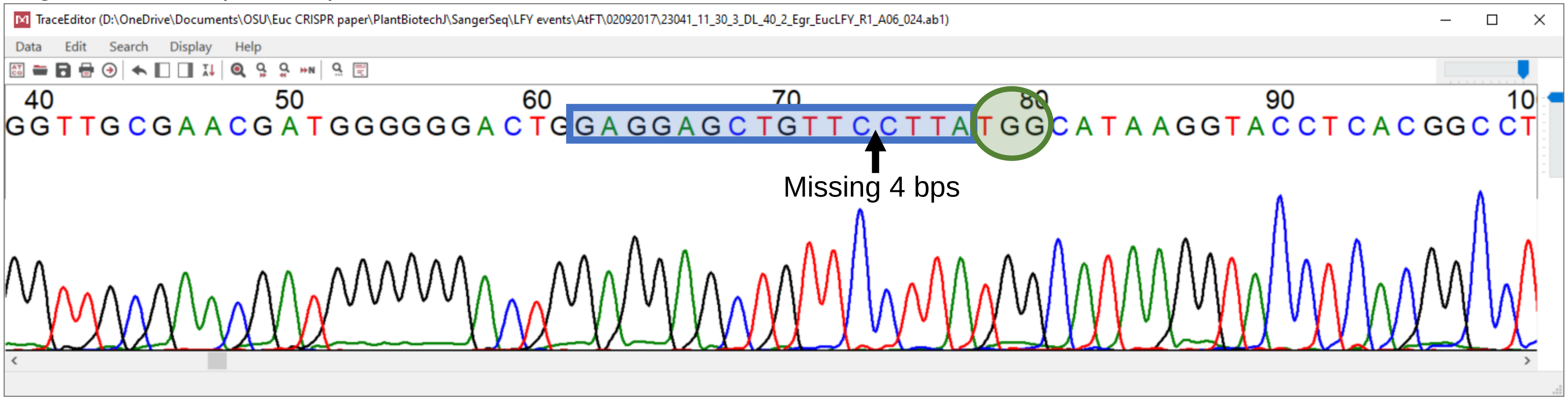

E. grandis allele (site two)

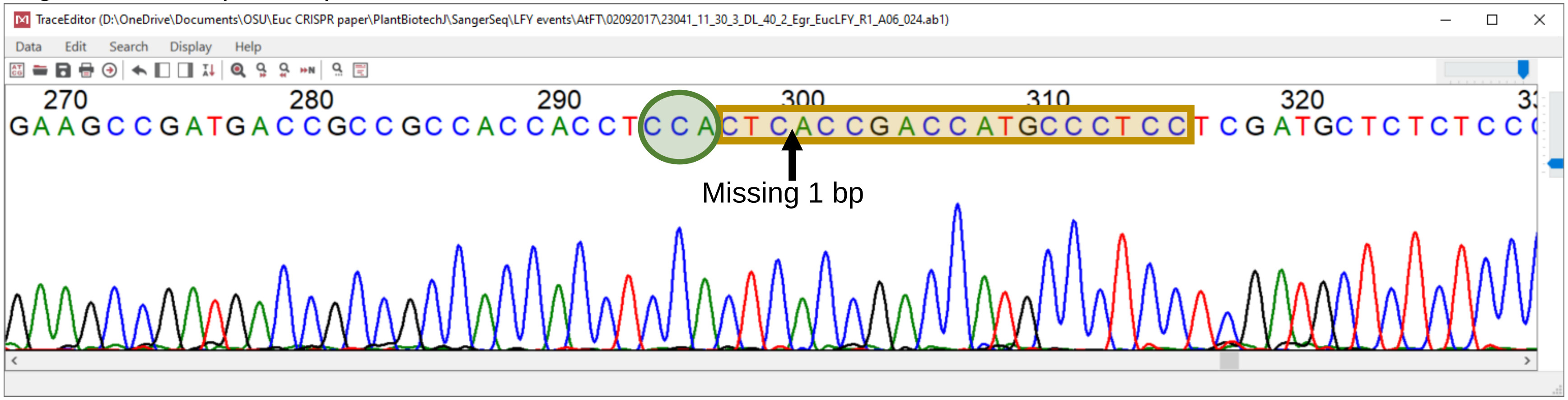

E. urophylla allele

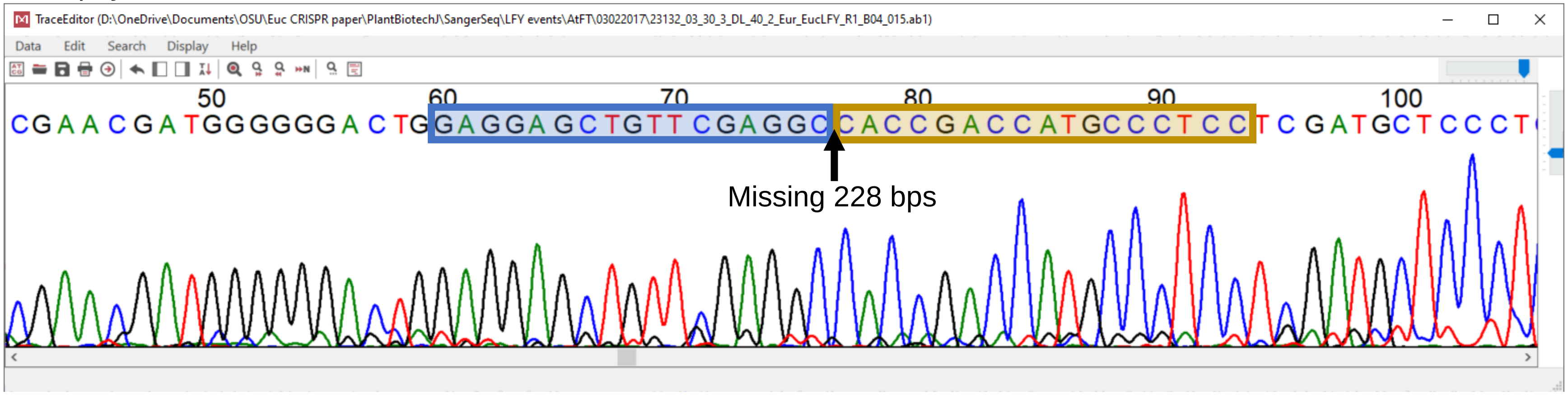

Fig. S3 Allelic chromatograms of FT-events in gene expression experiments. (**a**) Chromatograms of FT-only-control event FT-30. (**b**) Chromatograms of FT-Cas9-control event Cas9-30-14. (**c**) Chromatograms of FT-KO event 30-10 (transformed with ELFY-sg1). (**d**) Chromatograms of FT-KO event 30-11 (transformed with ELFY-sg1). (**e**) Chromatograms of FT-KO event 30-31 (transformed with ELFY-sg2). (**f**) Chromatograms of FT-KO event 30-45 (transformed with ELFY-sg2). (**g**) Chromatograms of FT-KO event 30-2 (transformed with ELFY-sg1sg2). (**h**) Chromatograms of FT-KO event 30-40 (transformed with ELFY-sg1sg2). The ELFY-sg1 binding site is highlighted with a blue box (i.e., target site one). The ELFY-sg2 binding site is highlighted with a tan box. Protospacer adjacent motif (PAM) sequences are highlighted with a green oval. Black arrows point to the location of the mutations.

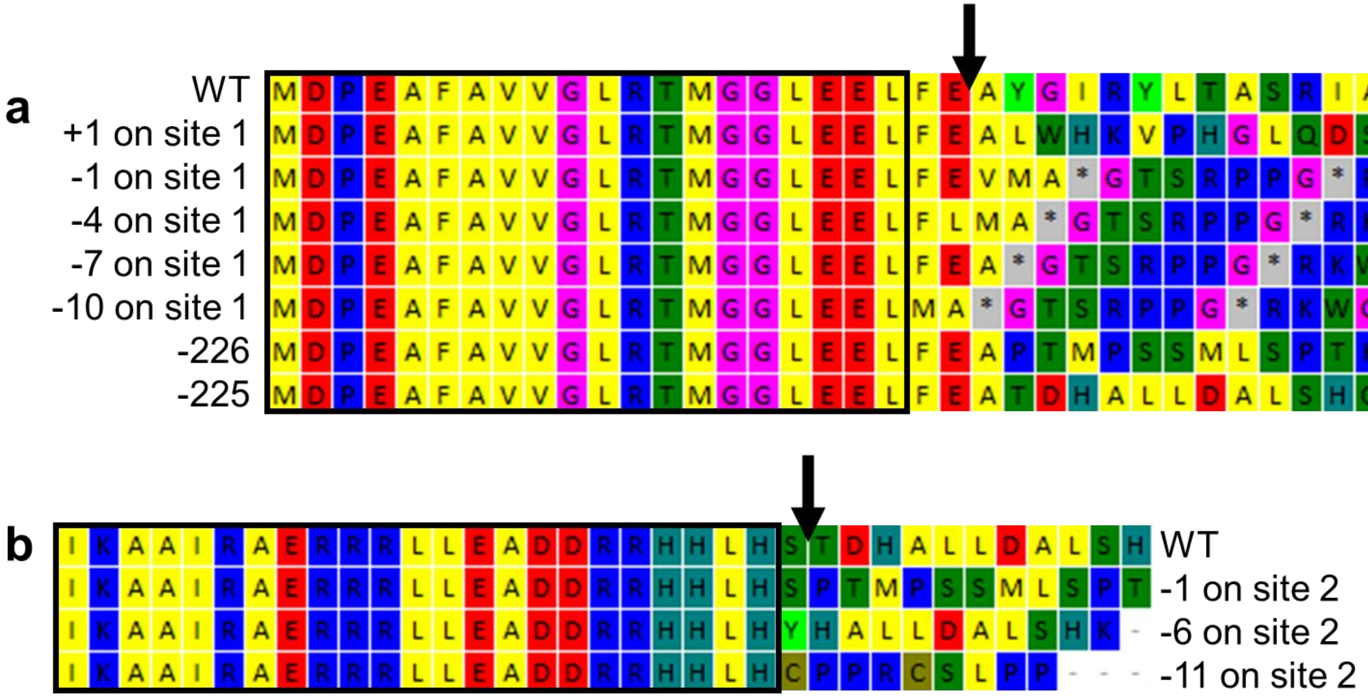

Fig. S4 Partial peptide alignment of the N-terminal in WT and some mutants. (**a**) Peptide sequence of the first 35 amino acids of the N-terminal motif in WT and KO events transformed with ELFY-sg1 (i.e., events with mutations on site one) and ELFY-sg1sg2 (i.e., events with a deletion from site one through site two) are shown. The last two peptide sequences shown have large deletions that reduced their exon size from 109 to 35. (**b**) Peptide sequence of the last 35 amino acids of the N-terminal motif in WT and KO events transformed with ELFY-sg2 (i.e., events with mutations on site 2). The black rectangles show the amino acids that remained unmodified. The black arrows indicate where the modifications to the peptide sequence are expected to happen for each target. All these peptide sequences belonged to confirmed KO events.

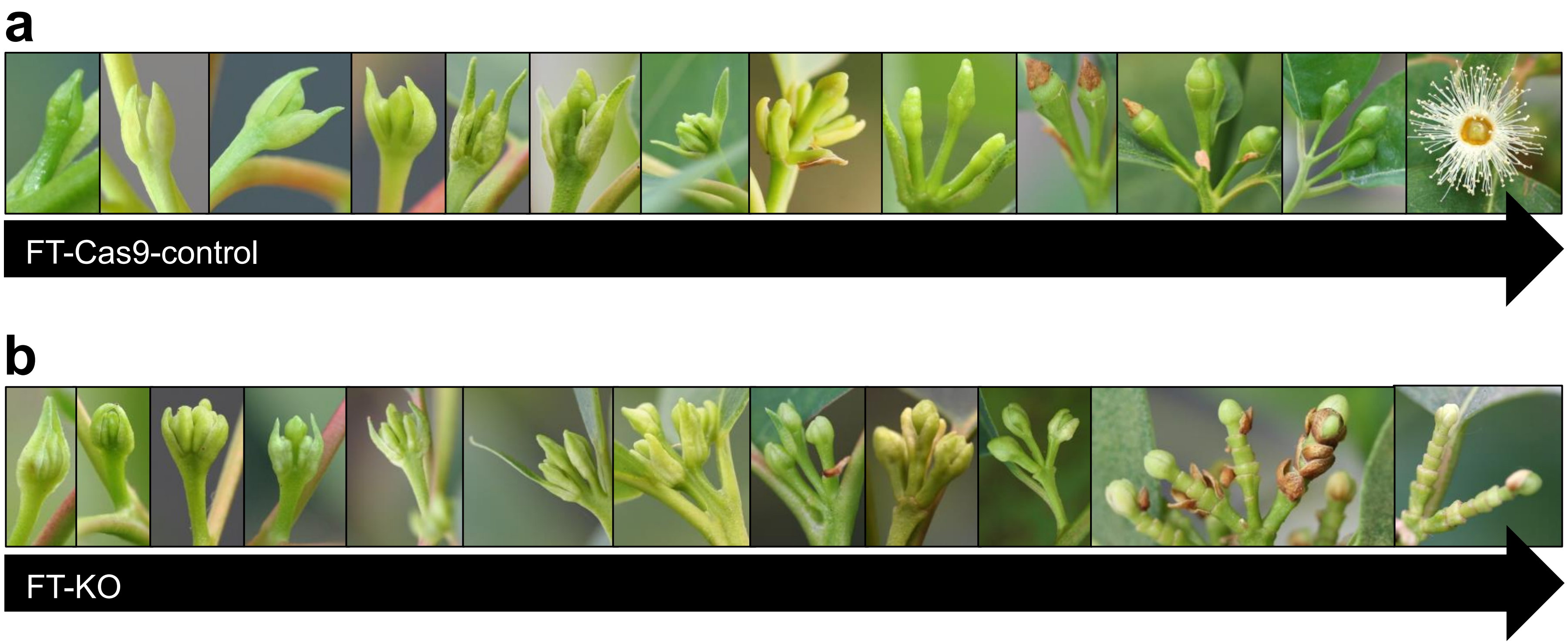

Fig. S5 Developmental sequence of flower formation in the greenhouse. (**a**) Flower buds and flowers of FT-Cas9-control events. The entire sequence spans approximately four months. (**b**) Flower buds of FT-KO events. The entire sequence spans approximately seven months.

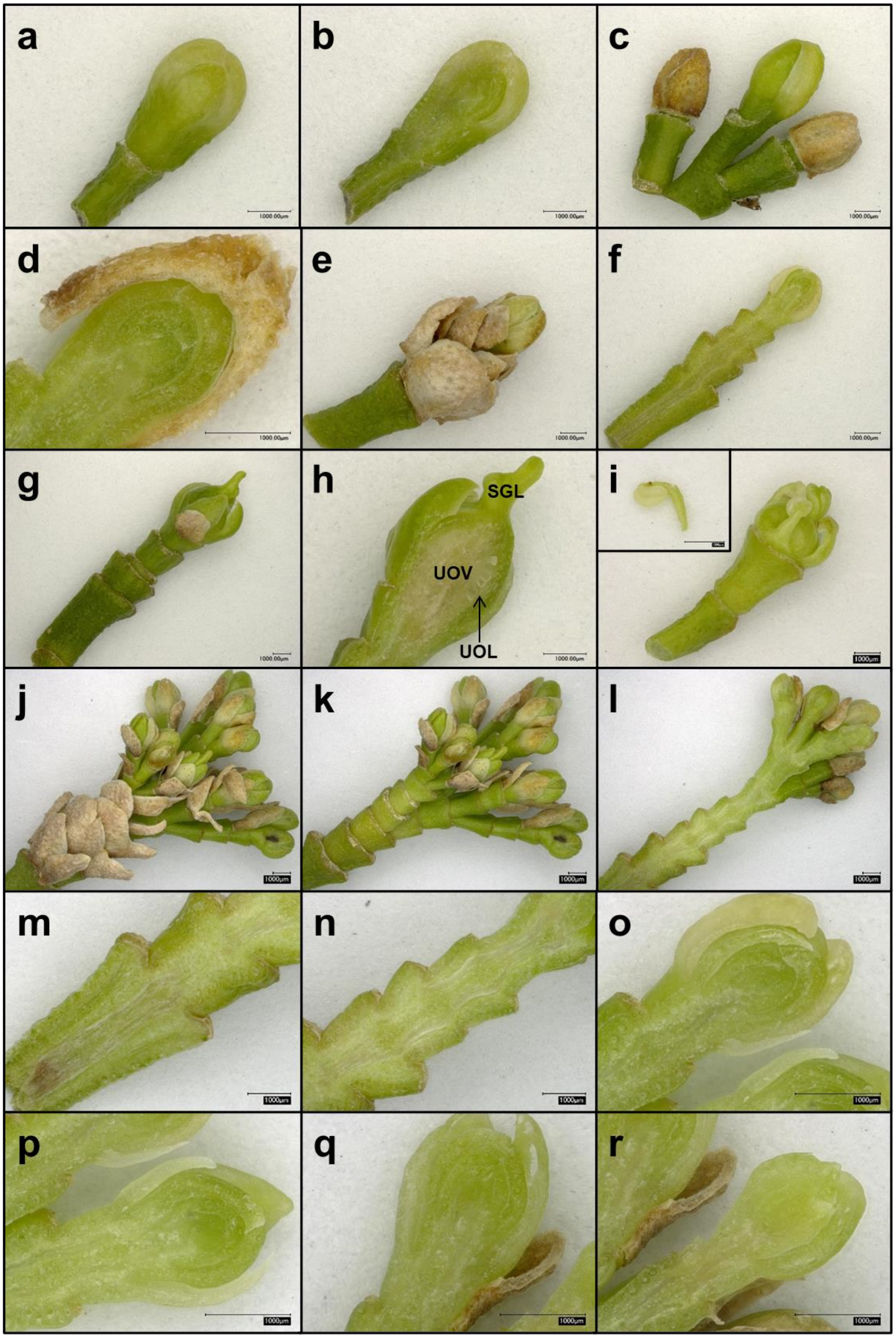
a
b
c
d
e
f
g
h
SGL
UOV
UOL
i
j
k
l
m
n
o
p
q
r

Fig. S6 Sterile floral-like buds seen in different FT-KO events. (**a** - **h**) Correspond to floral-like buds with underdeveloped ovules belonging to FT-KO event 30-6. (**a**) Early bud and (**b**) its longitudinal section with no sign of reproductive organs. (**c**) Umbel with more developed buds than (a) and (b). (**d**) Longitudinal section of bud in (c) with no reproductive organs. (**e**) Late bud with four layered pedicels surrounded by many bract-like organs. (**f**) longitudinal section of bud in (e) with no reproductive organs. (**g**) Late bud with four layered pedicels. (**h**) longitudinal section of bud in (g) with underdeveloped ovules. (**i**) Sterile bud from FT-KO event 30-30 with four underdeveloped stamen-like organs surrounding the hypanthium. One single stamen-like organ in photo insert. (**j** - **r**) Correspond to floral-like buds from FT-KO event 30-10 with many repeated bract-like and pedicel-like organs, and no underdeveloped ovules or anthers. (**j**) Umbel surrounded by many bract-like organs. (**k**) Umbel with bracts removed showing three pedicel-like repeated layers before splitting and generating more pedicel-like organs. (**l** - **r**) Longitudinal sections of the buds showing the lack of discernable ovules and stamens. SGL, stigma-like. UOL, underdeveloped ovules. UOV, underdeveloped ovary

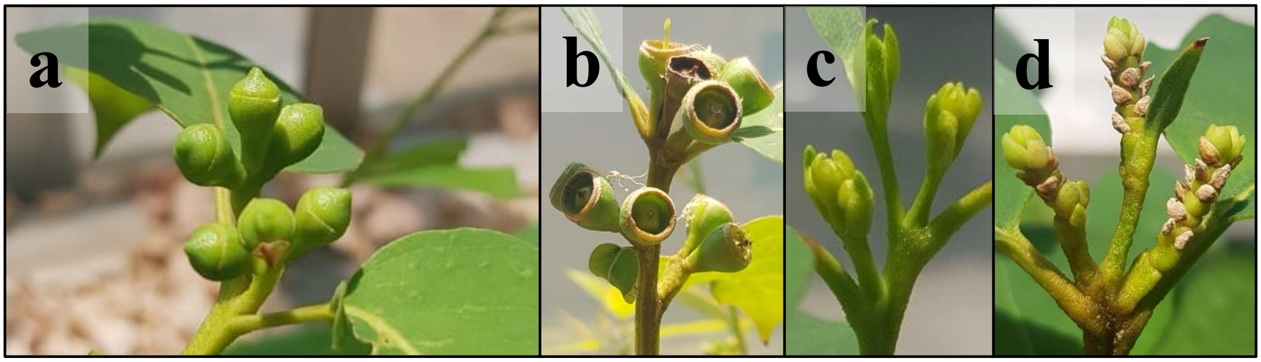

Fig. S7 Flower buds and flowers of FT-only and FT-KO events in a greenhouse trial at the University of Pretoria in South Africa. The FT-controls (i.e., FT-only-control and FT-Cas9-controls) and FT-KO events had essentially the same flowering phenotypes seen in Oregon. (**a**) Flowers buds from FT-only-control FT-30 with bracts and calycine opercula shed. (**b**) Developing seed capsules from FT-only-control FT-30 with stamens shed and stigmas dried out. (**c**) Three umbels early in development with early buds from FT=KO event 30-11. (**d**) Umbel with four mutant flowering buds from FT-KO event 4-66 with layers of pedicel-like and bract-like organs. The bract-like organs dry out and eventually fall off.

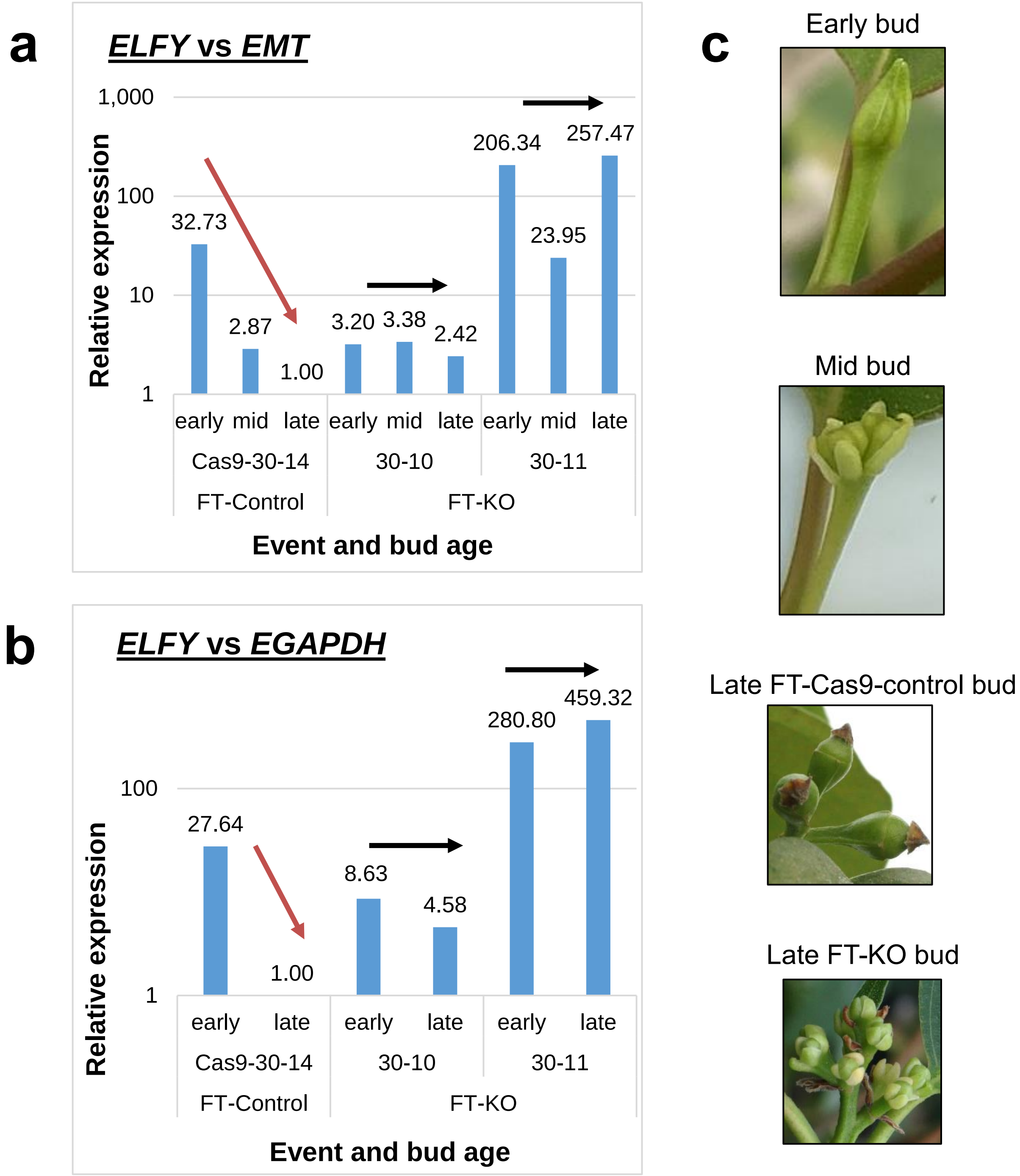
a
ELFY vs EMT
Relative expression
1,000
100
10
1
32.73
2.87
1.00
3.20
3.38
2.42
206.34
23.95
257.47
early mid late early mid late early mid late
Cas9-30-14
30-10
30-11
FT-Control
FT-KO
Event and bud age
b
ELFY vs EGAPDH
Relative expression
100
1
27.64
1.00
8.63
4.58
280.80
459.32
early late early late early late
Cas9-30-14
30-10
30-11
FT-Control
FT-KO
Event and bud age
c
Early bud
Mid bud
Late FT-Cas9-control bud
Late FT-KO bud

Fig. S8 *ELFY* gene expression from qPCR at three different bud development stages. (**a** and **b**) Early-, mid-, and late-bud *ELFY* gene expression seen in one FT-Cas9-control event, Cas9-30-14, and in two independent FT-KO events, 30-10 and 30-11, with (**a**) *EMT* and (**b**) *EGAPDH* as housekeeping reference gene. All qPCR reactions had three technical replicates. (**c**) Photos of the flower or floral-like buds at different stages. The y-axes are shown in a logarithmic scale to visualize all the values easily. Inferred, general expression patterns are shown by orange arrows for the FT-Cas9-control event and black arrows for the FT-KO events.

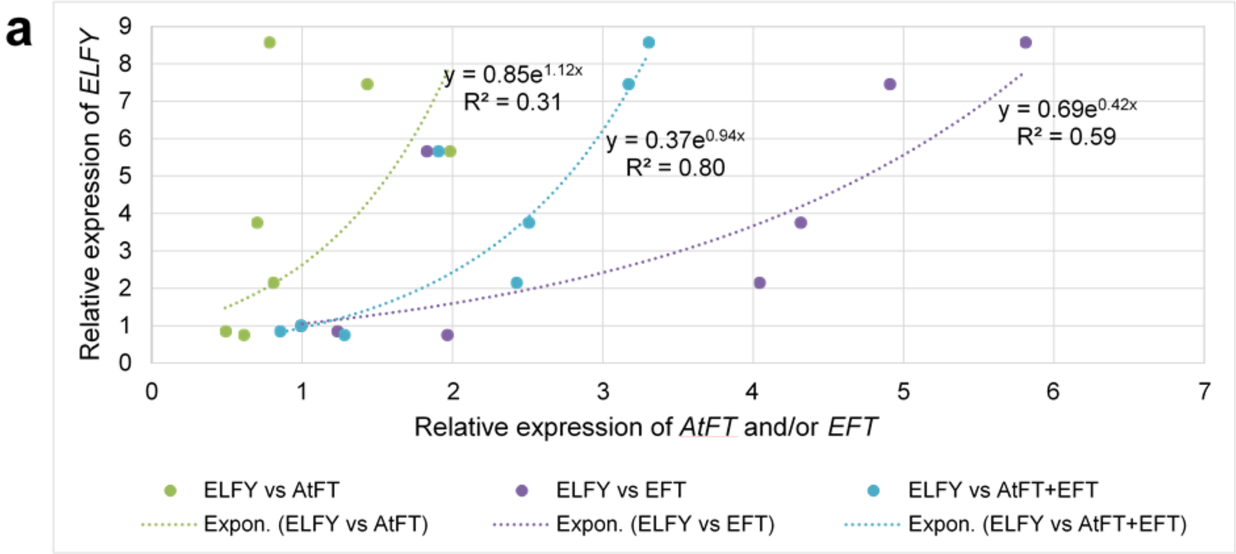

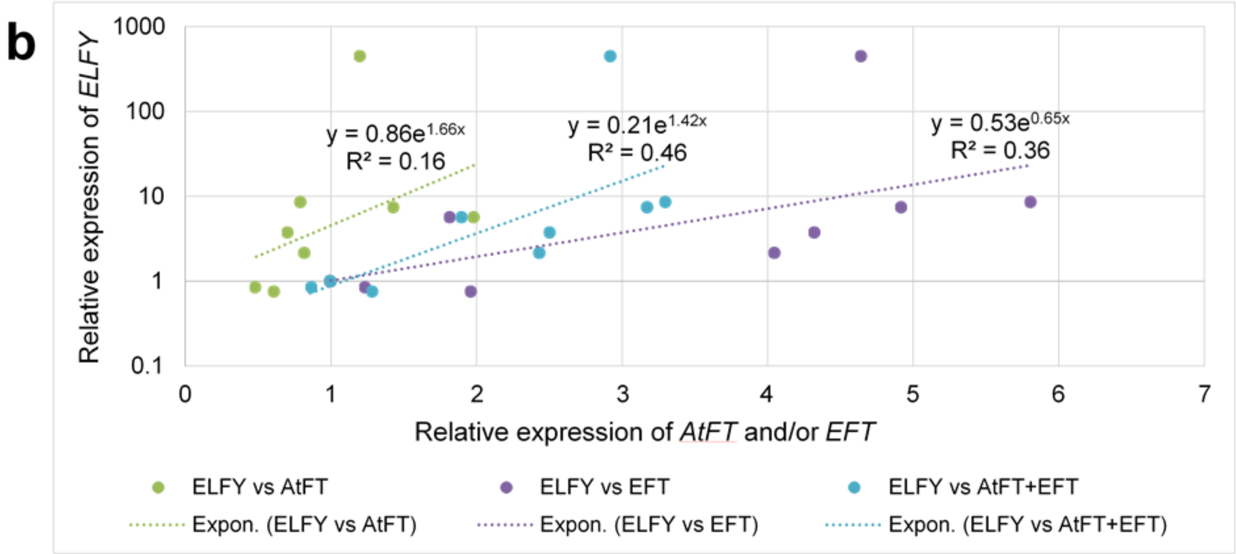

Fig. S9 Scattergram of the relative expression of *ELFY* across the relative expression of *AtFT* and/or *EFT*. (**a**) Semi-logarithmic graph with the highly expressing FT-KO event 30-11 included, and (**b**) linear graph without the FT-KO event 30-11. Exponential trendlines were fitted to each scattergram and coefficients of determination shown. Coefficient of determination (i.e., R-squared) were added to each scattergram to show the strength of the relationship between the relative expression of *ELFY* and the relative

expression of *AtFT* and/or *EFT*.

a

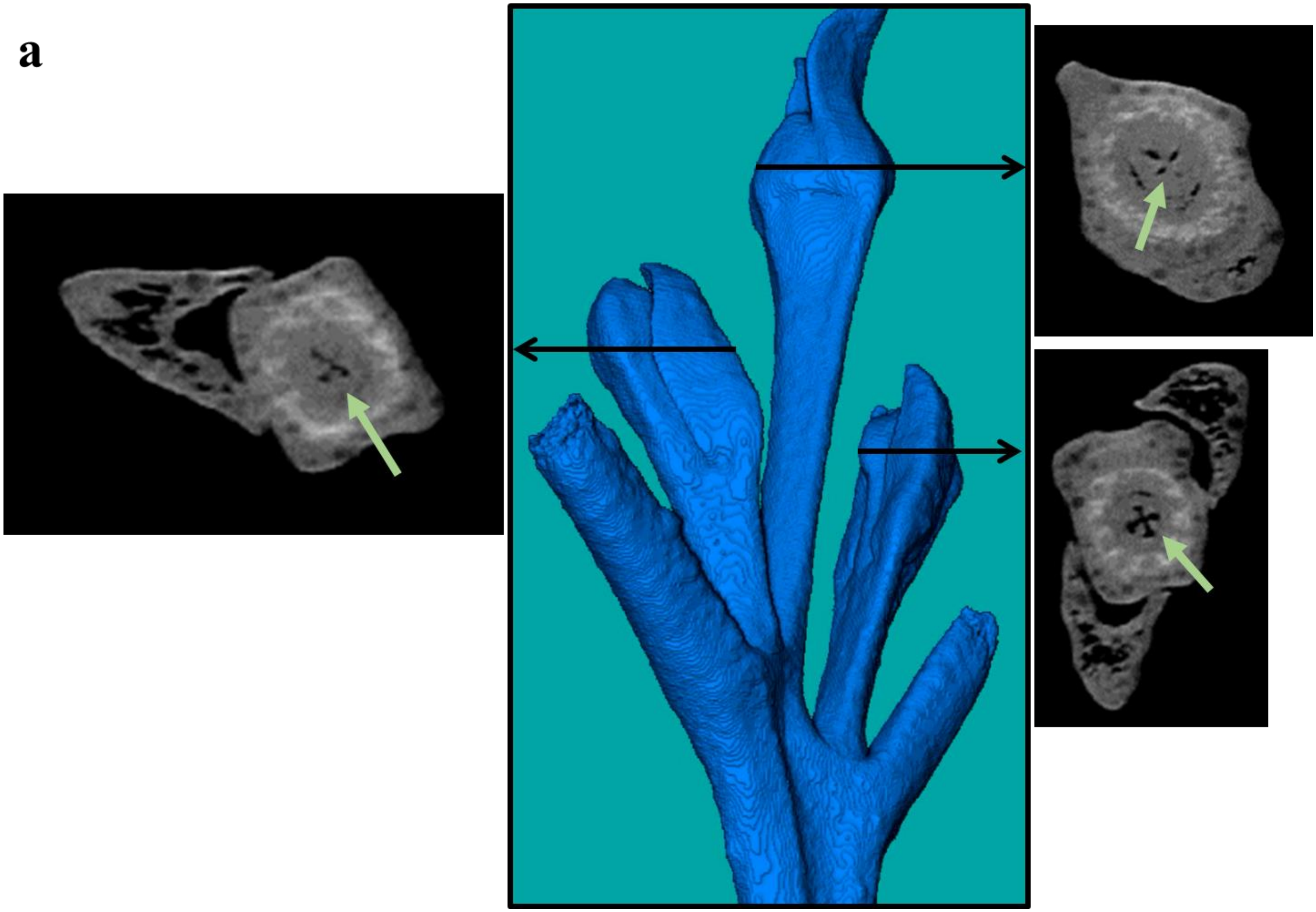

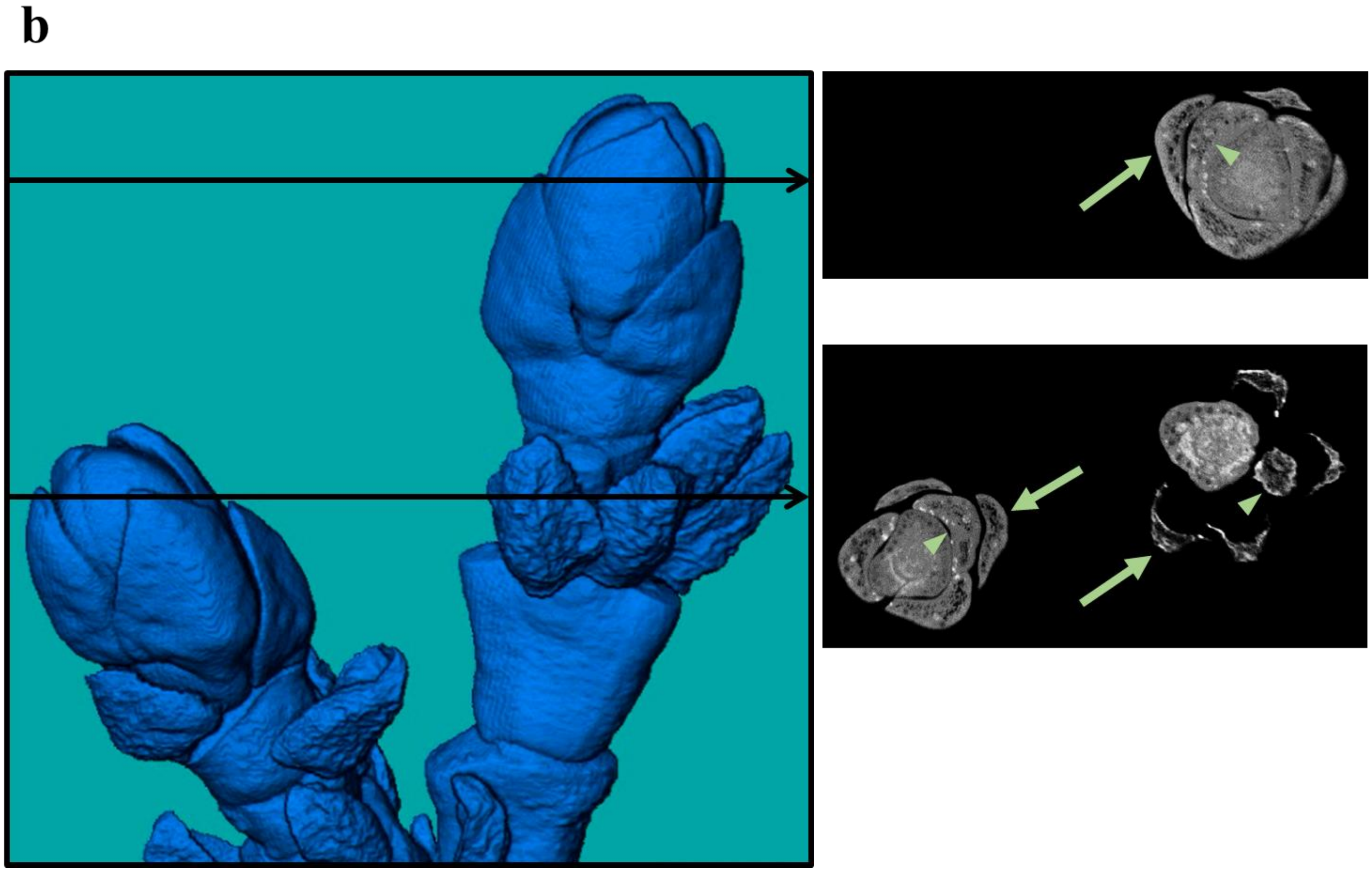

Fig. S10 3D representation of X-ray projections of inflorescences. (**a**) 3D representation of a young FT inflorescence after X-ray scanning. Arrows point to four locules where carpels were in development. The inflorescence was harvested from FT-only-control FT-30. (**b**) 3D representation of a ten-month-old inflorescence from FT-KO event 30-16 after X-ray scanning. Slices show repeated bract-like organs. Arrow points the outer most bract and arrowheads point to the next bract in the repeated succession. No reproductive organ development was seen in these or other cross-sections from the image of this event.

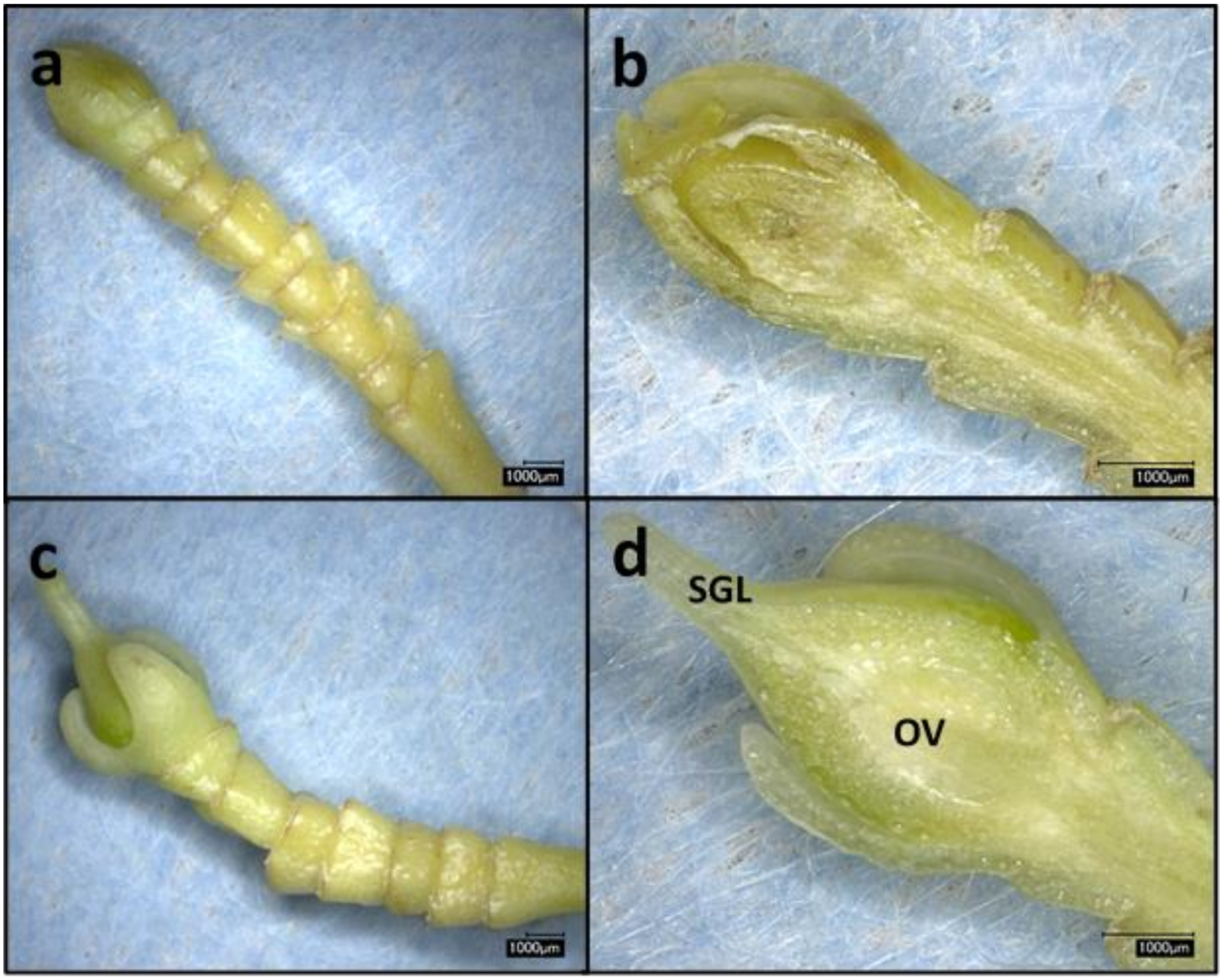

Fig. S11 Underdeveloped organs appeared occasionally in six-month-old or older buds from FT-KO events. The buds in these images belonged to FT-KO event 4-66. (**a**) Long-lived bud with nine visible layered pedicel-like organs. (**b**) No reproductive organs seen in longitudinal section of bud in (a). (**c**) Long-lived bud with seven visible layered pedicel-like organs, bract-like organ covering a hypanthium-like structure. (**d**) Undeveloped ovary with ovules and stigma-like organ seen inside dissected bud in (c). No male reproductive organs were visible. OV, ovary. SGL, stigma-like.

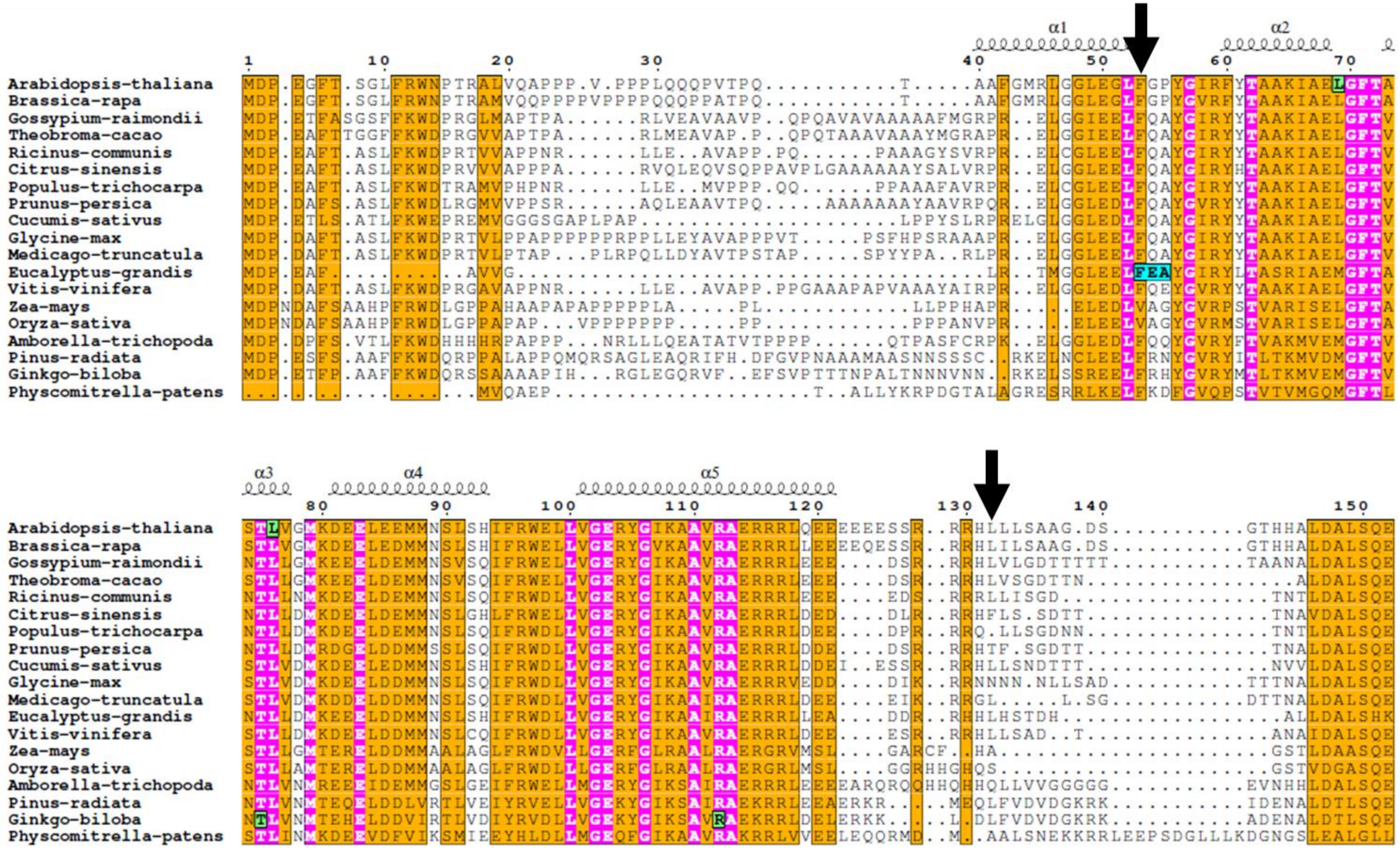

Fig. S12 Peptide alignment of the N-terminal domain in *LFY* and orthologous transcription factors. The three amino acids removed in event FT-KO 4-8 are in bold and inside cyan boxes. The two amino acids removed in event FT-KO 30-6 are the glutamic acid and alanine missing from event FT-KO 4-8. The amino acids modified by Siriwardana & Lamb (2012) and Sayou *et al.* (2016) are in bold and inside green boxes. Orange and pink indicate 70 and 100% similarity across all the sequences, respectively. Periods indicate no amino acid. The black arrows point to the location of the target sites where modifications to the peptide sequence would be expected to occur most frequently.

```
Egrandis   1 MDPEAFAVVGLRTMGGLEELFEAYGIRYLTASRIAEMGFTANTLLDMKEEELDDMMNSLS 60
4-1        1 MDPEAFAVVGLRTMGGLEELFEAYGIRYLTASRIAEMGFTANTLLDMKEEELDDMMNSLS 60
4-7        1 MDPEAFAVVGLRTMGGLEELFEAYGIRYLTASRIAEMGFTANTLLDMKEEELDDMMNSLS 60
4-72       1 MDPEAFAVVGLRTMGGLEELFEAYGIRYLTASRIAEMGFTANTLLDMKEEELDDMMNSLS 60
30-41      1 MDPEAFAVVGLRTMGGLEELFEAYGIRYLTASRIAEMGFTANTLLDMKEEELDDMMNSLS 60
             ************************************************************

Egrandis  61 HIFRWDLLVGERYGIKAAIRAERRRLLEADDHRRHHLHST-----DHALLDALSHK 112
4-1       61 HIFRWDLLVGERYGIKAAIRAERRRLLEADDHRRHHLHS------DHALLDALSHK 111
4-7       61 HIFRWDLLVGERYGIKAAIRAERRRLLEADDHRRHHLHSSPSAPSAHALLDALSHK 117
4-72      61 HIFRWDLLVGERYGIKAAIRAERRRLLEADDHRRHHLHS------DHALLDALSHK 111
30-41     61 HIFRWDLLVGERYGIKAAIRAERRRLLEADDHRRHHLHS-------HALLDALSHK 110
             ***************************************       **********
```

Fig. S13 Peptide alignment of the N-terminal domain of the FT-IFM events. The top row of the alignment shows the sequence of the N-terminal of the *Eucalyptus grandis* ELFY protein. The four events below (i.e., 4-1, 4-7, 4-72, and 30-41) were the in-frame-mutation (IFM) events. These events had in-frame-mutations in one allele (events 4-7, 4-72, and 30-41) or both alleles (event 4-1). The amino acids removed from all the FT-IFM events were not highly conserved in the 19-species alignment (Fig. S12). The amino acids in bold were highly conserved in the 19-species alignment (Fig. S12).

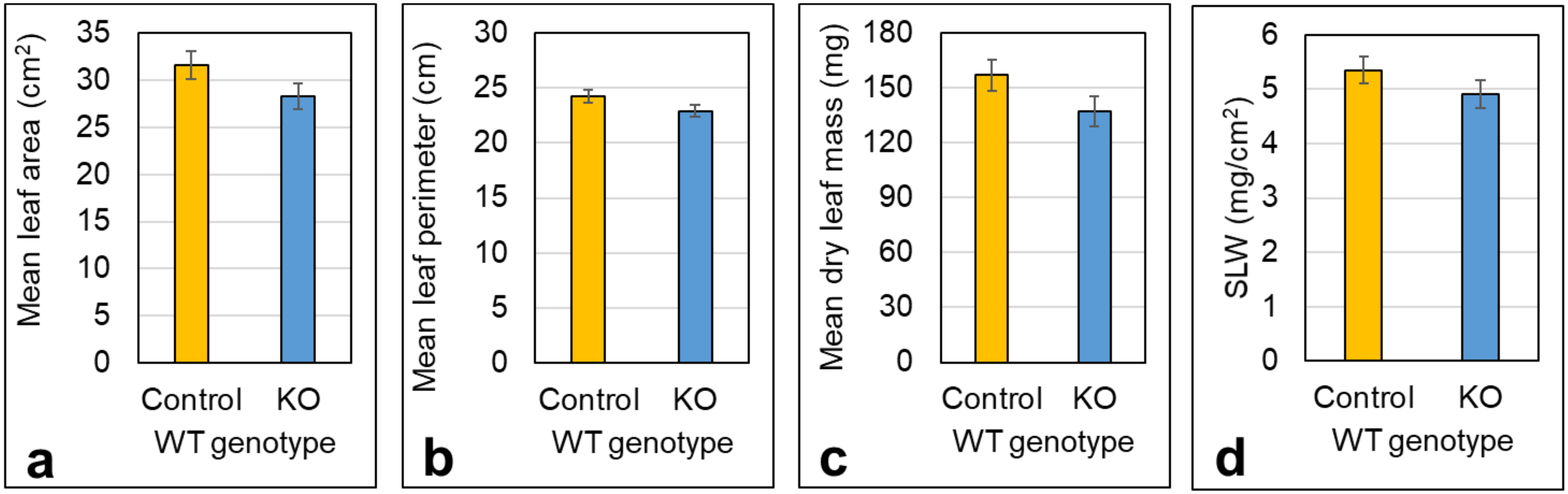

e

Contrast	Leaf area (cm^3)	P-value	Perimeter (cm)	P-value	Leaf dry mass (mg)	P-value	Specific leaf weight (mg/cm^3)	P-value
Control - KO	3.292	0.125	1.334	0.125	15.339	0.237	0.44568	0.235

Fig. S14 Leaf phenotypes of potted plants in WT trial. (**a**) Mean leaf area of KO and control events (i.e., escapes, WT, and Cas9-only). (**b**) Mean leaf perimeter. (**c**) Mean dry leaf weight. (**d**) Mean specific leaf weight (SLW). (**e**) Table of estimated mean differences and p-values corresponding to the Student's t-test on the means of control versus KO in area, leaf perimeter, leaf dry weight, and specific leaf weight. Error bars represent ± SE of means. KO, knock-out event. SLW, specific leaf weight.

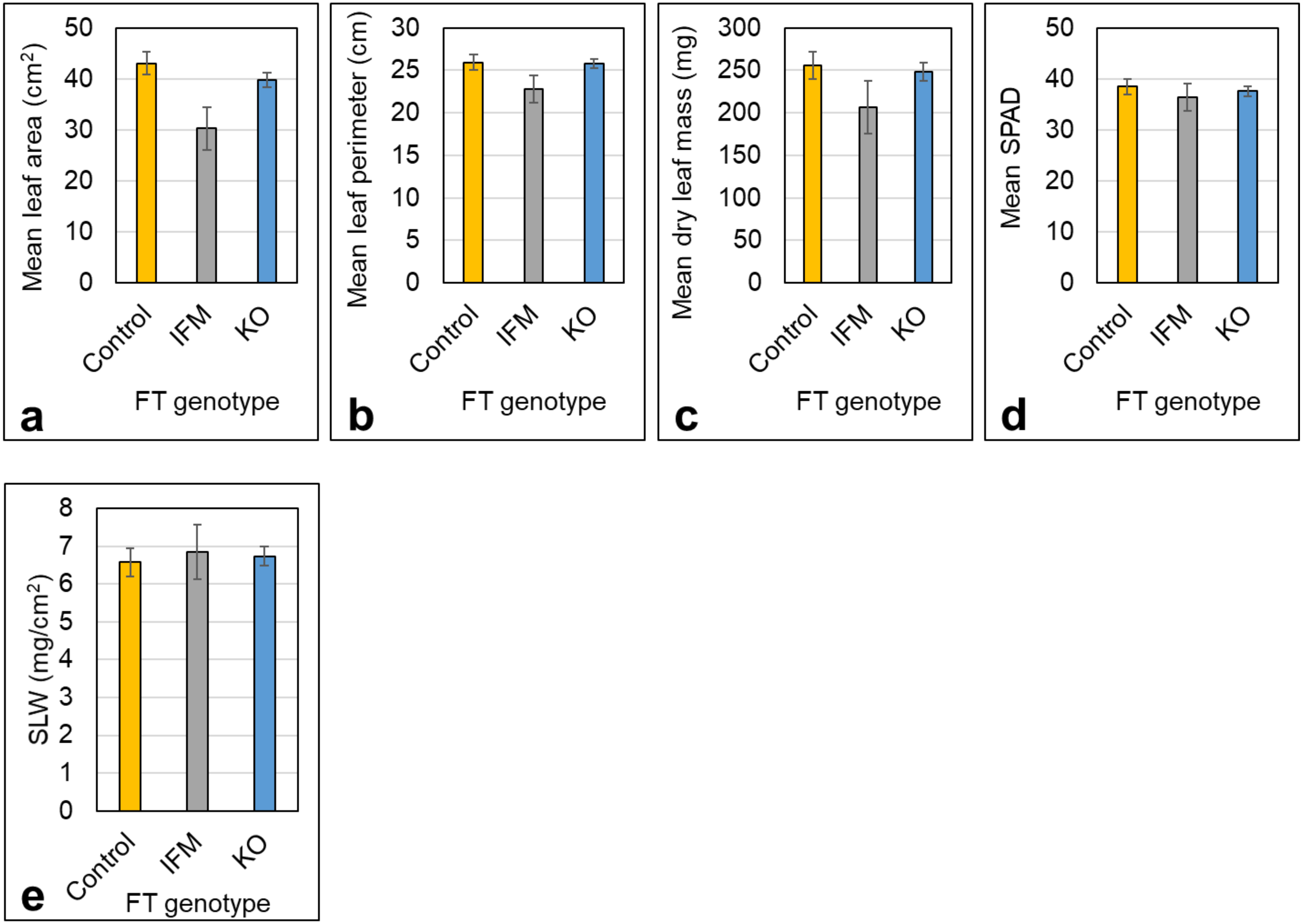

a
Mean leaf area (cm^2)
50
40
30
20
10
0
Control
IFM
KO
FT genotype
b
Mean leaf perimeter (cm)
30
25
20
15
10
5
0
Control
IFM
KO
FT genotype
c
Mean dry leaf mass (mg)
300
250
200
150
100
50
0
Control
IFM
KO
FT genotype
d
Mean SPAD
50
40
30
20
10
0
Control
IFM
KO
FT genotype
e
SLW (mg/cm^2)
8
7
6
5
4
3
2
1
0
Control
IFM
KO
FT genotype

f

Contrast	Leaf area (cm^2)	P-value	Perimeter (cm)	P-value	Leaf dry weight (mg)	P-value	RCD	P-value	SLW (mg/cm^2)	P-value
**Control - IFM**	12.898	**0.027**	3.210	0.205	51.678	0.320	2.049	0.789	-0.262	0.944
**Control - KO**	3.303	0.437	0.177	0.984	9.208	0.887	0.902	0.869	-0.158	0.934
**IFM - KO**	-9.595	0.090	-3.034	0.193	-42.470	0.411	-1.147	0.915	0.104	0.990

Fig. S15 Leaf phenotypes of potted plants in FT trial. (**a**) Mean leaf area of FT-KO plants, FT-IFM plants, and FT-control plants (i.e., FT-only-controls, FT-Cas9-controls, and FT-escape-controls). (**b**) Mean leaf perimeter. (**c**) Mean dry leaf weight. (**d**) Relative chlorophyll density (RCD; i.e., SPAD value). (**e**) Mean specific leaf weight (SLW). (**f**) Table of estimated mean differences and p-values corresponding to the t-test on the means of each contrast for leaf area, leaf perimeter, dry leaf weight, relative chlorophyll density, and specific leaf weight. The one statistically significant t-test result ($P<0.05$) is highlighted. Error bars represent ± SE of means. IFM, in-frame mutant. KO, knock-out event. SLW, specific leaf weight.

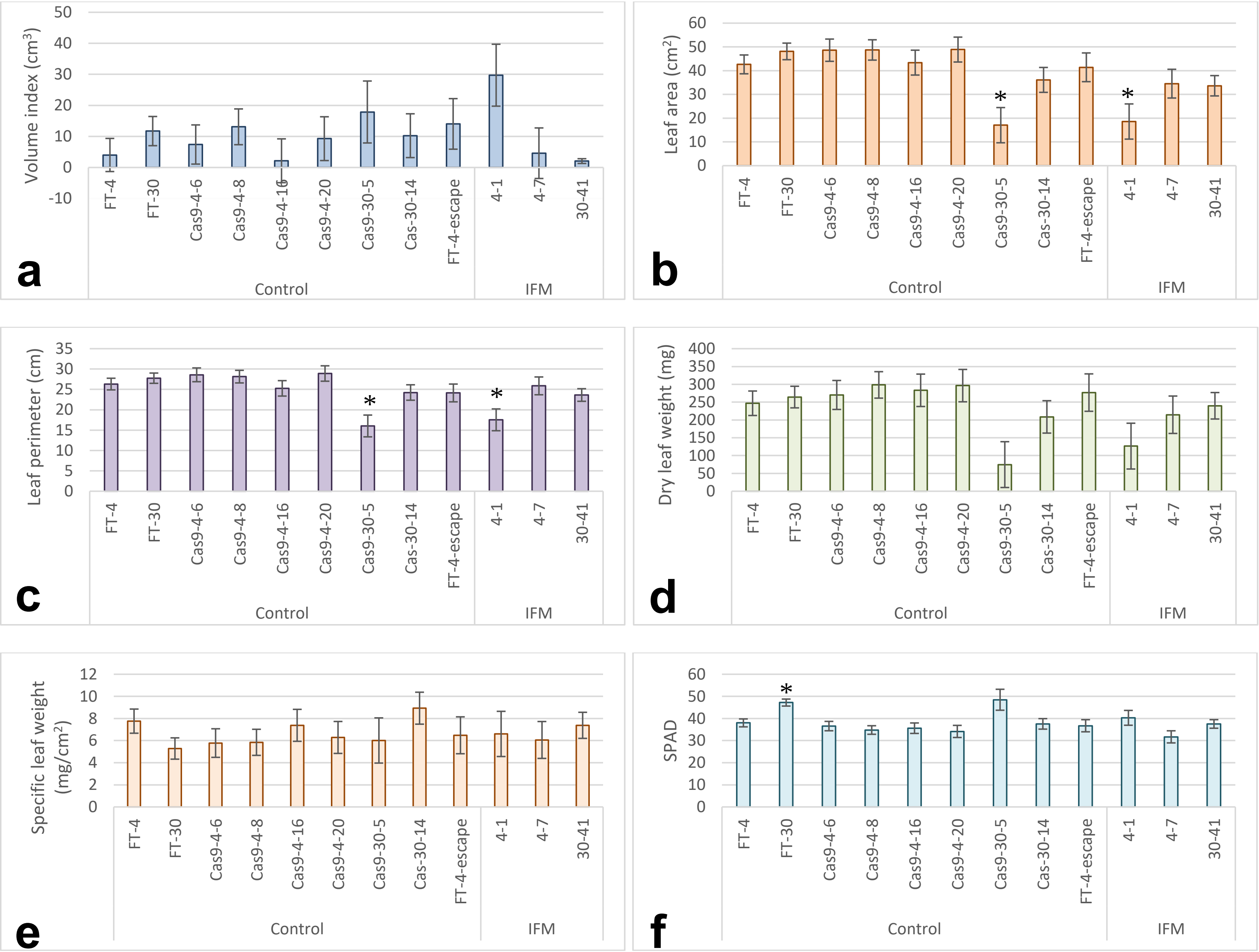
a
Volume index (cm³)
50
40
30
20
10
0
-10
FT-4
FT-30
Cas9-4-6
Cas9-4-8
Cas9-4-16
Cas9-4-20
Cas9-30-5
Cas-30-14
FT-4-escape
4-1
4-7
30-41
Control
IFM
b
Leaf area (cm²)
60
50
40
30
20
10
0
*
*
c
Leaf perimeter (cm)
35
30
25
20
15
10
5
0
*
*
d
Dry leaf weight (mg)
400
350
300
250
200
150
100
50
0
e
Specific leaf weight (mg/cm²)
12
10
8
6
4
2
0
f
SPAD
60
50
40
30
20
10
0
*

Fig. S16 Leaf phenotypes of FT-IFM and FT-control events. (**a**) Mean volume index, (**b**) mean leaf area, (**c**) mean leaf perimeter, (**d**) mean dry leaf weight. (**e**) mean specific leaf weight (SLW), and (**f**) relative chlorophyll density (RCD; i.e., SPAD value) of FT-IFM and FT-control events. The controls include FT-only-control events FT-4 and FT-30, the FT-Cas9-control events Cas9-4-6, Cas9-4-8, Cas9-4-16, Cas-9-20, Cas9-30-5, and Cas-30-14, and FT-escape-control event FT-4-escape. Error bars represent ± SE of means. An asterisk above a bar indicates that the average value of that trait for that event was found significantly different (significance level of 0.05) compared to the average of one or more other events (control or IFM). IFM, in-frame mutant.

Table S1 Glossary.

**Term**	**Meaning**
AG	*AGAMOUS*
AP3	*APETALA3*
CAL	*CAULIFLOWER*
CIM	callus induction medium
ELFY	Homolog of *LFY* (*Arabidopsis*) in *Eucalyptus*
ELFY-sg1	CRISPR Cas9 construct targeting the first site on the *ELFY* gene
ELFY-sg2	CRISPR Cas9 construct targeting the second site on the *ELFY* gene
ELFY-sg1sg2	CRISPR Cas9 construct targeting simultaneously the first site and the second site on the *ELFY* gene
Escape	Non-transgenic but *Agrobacterium* cocultivated and regenerated
KO	Knock-out events; events predicted to have alterations in their floral morphology based on expected protein translations
FMI	floral meristem identity
FOI	floral organ identity
FPI	floral pathway integrator
FT	*FLOWERING LOCUS T*
FT CRISPR Cas9	Early-flowering background transformed with sgRNA transcriptional unit and Cas9 transgene
FT-Cas9-control	Early-flowering background transformed with Cas9 transgene only; empty-vector
FT-escape-control	Early-flowering background that was not transformed but went through *Agrobacterium* cocultivation and plant regeneration
FT-only-control	Original insertion events transformed with *AtFT*; early-flowering background lines
FUL	*FRUITFULL*
GAPDH	*GLYCERALDEHYDE-3-PHOSPHATE DEHYDROGENASE*

IFM	In-frame mutant; these had modified amino acid/s but no frameshifts. No phenotypic differences seen.
LFY	*LEAFY*
MT	*METALLOTHIONEIN*
PI	*PISTILLATA*
Ramet	Individual clone of a specific transgenic event
SEM	shoot elongation medium
sgRNA	single guide RNA
SHP2	*SHATTERPROOF 2*
SIM	shoot induction medium
SMM	shoot multiplication medium
SNP	single nucleotide polymorphism
SP7	*Eucalyptus grandis x urophylla* clone
SPL3	*SQUAMOSA PROMOTER BINDING PROTEIN-LIKE 3*
SPL9	*SQUAMOSA PROMOTER BINDING PROTEIN-LIKE 9*
STK	*SEEDSTICK*
WT (Wild type)	not cocultivated but micropropagated prior to transplant into greenhouse

Table S2 Primers.

Primer name	Primer sequence (5' to 3')	Use	Reference
AtU626_F1	CTTCAAAAGTCCCACATCGC	Genotyping for presence of transgene	Elorriaga *et al.* (2018)
sgRNA_R1	GCCGCCAGTGTGATGGATA		
Cas9_F1	CACGACGGAGACTACAAGGA		
RB_R1	CGGATAAACCTTTTCACGCCC		
Egrandis_F3	GCGCGAGAATGGATCCAGAA	Amplifying the *LFY* allele from *E. grandis*	This study
Egrandis_R1	GAGGCCGAGTTAAGTTACCTTT		
Euro_F3	GCGCGAGAATGGATCCAGAG	Amplifying the *LFY* allele from *E. urophylla*	
Euro_R1	GAGGCCGAGTTAAGGTACCTTG		
ATFT_FWD	GGCCAAAGAGAGGTGACTAATG	Gene expression	
ATFT_REV	GGTCTTCTCCACCAATCTCAAC		
EAG_FWD	GGCATATGAACTCTCCGTTCTT		
EAG_REV	TACCTCTCGATGGTTGCTTTG		
EAP3_FWD	GGGAAGGACTGAATGGTATGAG		
EAP3_REV	TTGATTGCCGAGCGTCTT		
ECAL_FWD	TCAAGTGCAGGGAAGTTGG		
ECAL_REV	CTCAAGGGATCAAGCTCTTCTC		
EFT_FWD	CCAGCCCTAGTGATCCTAATCT		
EFT_REV	GGACTTTCGTAGCACACAATCT		
EFUL1_FWD	CAACCTGCTTACAAGGAAAGTAAAG		
EFUL1_REV	AGAACAACAGGTGAGTCAAGG		
EFUL2_FWD	CATCAGCTTGACACTGCTCTTA		
EFUL2_REV	TGTTCTTGCAGCGCCTTAT		
EGAPDH_FWD	GTCTCCGTTTTCGGCATCAGGAAC		
EGAPDH_REV	GCGGAGATGACAACCTTCTTAGCAC		
ELFY_FWD	CTTCGCAAGAGCTTCAAGGA		

ELFY_REV	CATTGAAGATGGCGTCGATGT		
EMT_FWD	CCGTAAATCTGAGAATGTCTTGC		
EMT_REV	ATGCTCTCGGCGTTGTAG		
EPI_FWD	CGGTGAAATCGACAGAATCAAG		
EPI_REV	AGGACATCATCTAGGACAATGAG		
ESHP2_FWD	CGTGGCAGGCTCTATGAATATG		
ESHP2_REV	GAAACGGACTGTGGATGTGAG		
ESPL3_FWD	GGAGAAAGAGCTCTCACGATTC		
ESPL3_REV	AGAGACCCACGTATCCATTCT		
ESPL9_FWD	ACTCTCGCTTCTCTTCCTCTAT		
ESPL9_REV	CCCGGATATGCACTGAAGTC		
ESTK_FWD	CTCTACGAGTACTCCAACAACAG		
ESTK_REV	TGGGCATTGATCTCTGTGAC		

Table S3 Transformation efficiency rates.

FT trial

FT + CRISPR	No of explants co-cultivated	No of explants with shoots	No of transgenic shoots (PCR)	Efficiency (%)
**ELFY-sg1**	1,266	142	36	2.8
**ELFY-sg2**	896	178	42	4.7
**ELFY-sg1sg2**	869	135	35	4.0
**Cas9**	383	57	13	3.4

WT trial

WT + CRISPR	No of explants co-cultivated	No of explants with shoots	No of transgenic shoots (PCR)	Efficiency (%)
**ELFY-sg1**	425	216	9	2.1
**ELFY-sg2**	461	198	5	1.1
**ELFY-sg1sg2**	501	231	12	2.4
**Cas9**	289	116	3	1.0

Table S4 Gene names and IDs for qPCR experiments. The orthologs in Eucalyptus were determined from a previously published floral transcriptome Vining et al. (2015) and an unpublished RNA-seq database.

**Gene name in Arabidopsis**	**Gene ID in *Arabidopsis thaliana* Araport11**	**Gene ID in *Eucalyptus grandis* version 2.0**
AGAMOUS (*AG*)	At4G18960	Eucgr.E02863
APETALA 3 (*AP3*)	At3G54340	Eucgr.F01615
CAULIFLOWER (*CAL*)	At1G26310	Eucgr.I02059
FLOWERING LOCUS T (*FT*)	At1G65480	Eucgr.B01458
FRUITFULL (*FUL)*	At5G60910	Eucgr.B00634
FRUITFULL (*FUL)*	At5G60910	Eucgr.K02547
glyceraldehyde-3-phosphate dehydrogenase (GAPDH)	At1G13440	Eucgr.H04673
LEAFY (LFY)	At5G61850	Eucgr.K02192
PISTILLATA (*PI*)	At5G20240	Eucgr.E01007
SEEDSTICK (*STK*)	At4G09960	Eucgr.F02981
SHATTERPROOF 2 (*SHP2)*	At2G42830	Eucgr.K01195
SQUAMOSA PROMOTER BINDING PROTEIN-LIKE 3 (*SPL3*)	At2G33810	Eucgr.D02505
SQUAMOSA PROMOTER BINDING PROTEIN-LIKE 9 (*SPL9*)	At2G42200	Eucgr.K01828

Table S5 Predicted knock-out (i.e., loss-of-function) rates based on the occurrence of frameshifts, large deletions (i.e. >=222 bp), and deletions of highly conserved amino acids. KO, knock-out (i.e., loss-of-function) mutant. WT, predicted wild-type peptide.

Population	Total events (alleles)	Predicted phenotype	N° events
**FT LFY-CRISPR**	59 (118)	KO	53 (90%)
		WT	6 (10%)
**WT LFY-CRISPR**	9 (18)	KO	9 (100 %)
		WT	0 (0%)
**All eucalypt**	**68 (136)**	KO	62 (91%)
		WT	6 (9%)

Table S6 Inventory of plants in the greenhouse trials.

WT trial

**Type**	**Event n°**	**Ramet n°**
CRISPR Cas9 transgenic	9	41
Cas9 transgenic control	3	18
Escapes (not transgenic)	2	12
WT control (not transgenic)	1	7
**TOTAL**	**15**	**78**

FT trial

**Type**	**Event n°**	**Ramet n°**
FT CRISPR Cas9 transgenic (originally transformed with FT overexpression and retransformed with ELFY-sg1, ELFY-sg2, or ELFY-sg1sg2)	42	166
FT Cas9 transgenic control (i.e., empty vector)	6	25
FT-only transgenic control	2	23
WT control (not transgenic)	1	5
**TOTAL**	**51**	**219**

Table S7 Phenotypes seen in FT-KO events kept in the greenhouse. Each row corresponds to one FT-KO event. CRISPR corresponds to the specific CRISPR Cas9 nuclease in the FT-KO event. The mutations seen in each site and each allele are specified. The total number of ramets and the number of ramets that flowered are also recorded. The specific peptide modification, the functionality of peptide, and the floral phenotype recorded are also indicated. AAD, amino acid deletion. AAI, amino acid insertion. CAAD, conserved amino acid deletion. Eg, Eucalyptus grandis. Euro, Eucalyptus urophylla. FS, frameshift. FSinNtem, frameshit in N-terminal. InvNterm, inversion in the N-terminal. NtermD, N-terminal deletion. ORGANLESS, no underdeveloped reproductive organs in five layered pedicels. NONE, plants did not flower. UBF, underdeveloped bisexual flower. UFF, underdeveloped female flower.

Event	CRISPR	Eg site 1	Eg site 2	Euro site 1	Euro site 2	N° of ramets	Flowering ramets	Peptide mod.	Functional peptide	Floral phenotype
4-46	EgLFY-sg1sg2	5bp deletion, 1bp insertion + chimera	1bp deletion	228bp deletion		5	5 (100.0 %)	NtermD & FS	No	UBF
4-55	EgLFY-sg1sg2	225bp deletion		222bp insertion		6	6 (100.0 %)	NtermD & FS	No	UBF
30-30	EgLFY-sg1sg2	7bp deletion	2bp deletion	228bp deletion		3	3 (100.0 %)	NtermD & FSinNtem	No	UBF
30-33	EgLFY-sg1sg2	261bp deletion		264bp deletion		5	5 (100.0%)	NtermD & NtermD	No	UBF
30-42	EgLFY-sg1sg2	Inversion (225bp)		228bp deletion		2	2 (100.0 %)	NtermD & InvNterm	No	UBF
4-8	EgLFY-sg1	9bp deletion	NA	1bp insertion	NA	3	3 (100.0 %)	CAAD & FS	No	UFF
4-33	EgLFY-sg1	1bp insertion	NA	7bp deletion	NA	4	4 (100.0 %)	FS & FS	No	UFF
4-54	EgLFY-sg1	4bp deletion	NA	1bp deletion	NA	6	6 (100.0 %)	FS & FS	No	UFF
4-59	EgLFY-sg1	32bp deletion	NA	1bp insertion	NA	6	6 (100.0 %)	FS & FS	No	UFF
4-60	EgLFY-sg1	1bp insertion	NA	10bp deletion	NA	4	4 (100.0 %)	FS & FS	No	UFF

30-6	EgLFY-sg1	6bp deletion	NA	3bp deletion	NA	1	1 (100.0 %)	CAAD & CAAD	No	UFF
30-11	EgLFY-sg1	4bp deletion	NA	4bp deletion	NA	6	6 (100.0 %)	FS & FS	No	UFF
4-9	EgLFY-sg1sg2	31bp deletion	3bp deletion	229bp deletion		6	6 (100.0 %)	FS & FS	No	UFF
4-66	EgLFY-sg1sg2	7bp deletion	1bp deletion	8bp deletion	1bp insertion	6	6 (100.0 %)	FS & FS	No	UFF
4-10	EgLFY-sg1	1bp insertion	NA	10bp deletion	NA	4	4 (100.0 %)	FS & FS	No	ORGANLESS
4-12	EgLFY-sg1	4bp deletion	NA	2bp deletion	NA	3	3 (100.0 %)	FS & FS	No	ORGANLESS
4-34	EgLFY-sg1	1bp insertion	NA	7bp deletion	NA	1	1 (100.0 %)	FS & FS	No	ORGANLESS
30-2	EgLFY-sg1	1bp insertion	NA	10bp deletion	NA	3	3 (100.0 %)	FS & FS	No	ORGANLESS
30-10	EgLFY-sg1	8bp deletion	NA	5bp deletion	NA	5	5 (100.0 %)	FS & FS	No	ORGANLESS
4-4	EgLFY-sg2	NA	1bp deletion	NA	7bp deletion	2	2 (100.0 %)	FS & FS	No	ORGANLESS
4-74	EgLFY-sg2	NA	1bp deletion	NA	1bp deletion	3	3 (100.0 %)	FS & FS	No	ORGANLESS
30-4	EgLFY-sg2	NA	2bp deletion	NA	5bp deletion	2	2 (100.0 %)	FS & FS	No	ORGANLESS
30-5	EgLFY-sg2	NA	1bp deletion	NA	11bp deletion	3	3 (100.0 %)	FS & FS	No	ORGANLESS
30-31	EgLFY-sg2	NA	1bp insertion	NA	26bp deletion	2	2 (100.0 %)	FS & FS	No	ORGANLESS
30-45	EgLFY-sg2	NA	1bp deletion	NA	1bp deletion	3	3 (100.0 %)	FS & FS	No	ORGANLESS
4-37	EgLFY-sg1sg2	261 bp deletion and 2bp insertion		36bp insertion	1bp deletion	3	3 (100.0 %)	FS & FS	No	ORGANLESS

4-65	EgLFY-sg1sg2	22bp deletion	1bp deletion	Inversion (228 bp)		4	2 (50.0 %)	FS & FS	No	ORGANLESS
30-1	EgLFY-sg1sg2	Inversion (225bp)		8bp deletion	3bp deletion	5	5 (100.0 %)	FS & FS	No	ORGANLESS
30-2	EgLFY-sg1sg2	2bp deletion	1bp deletion	3bp deletion	1bp deletion	5	5 (100.0 %)	FS & FS	No	ORGANLESS
30-3	EgLFY-sg1sg2	2bp insertion	1bp insertion	7bp deletion	1bp deletion	1	1 (100.0 %)	FS & FS	No	ORGANLESS
30-16	EgLFY-sg1sg2	226bp deletion		1bp insertion	1bp deletion	1	1 (100.0 %)	FS & FS	No	ORGANLESS
30-40	EgLFY-sg1sg2	4bp deletion	1bp deletion	228bp deletion		2	2 (100.0 %)	FS & FS	No	ORGANLESS
4-1	EgLFY-sg2	NA	3bp deletion	NA	3bp deletion	7	7 (100.0 %)	AAD & AAD	Yes	Normal flower
4-7	EgLFY-sg2	NA	15bp insertion	NA	16bp deletion	3	2 (66.7 %)	AAI & FS	Yes	Normal flower
4-72	EgLFY-sg2	NA	3bp deletion	NA	5bp deletion	6	6 (100.0 %)	AAD & FS	Yes	Normal flower
30-41	EgLFY-sg2	NA	1bp deletion	NA	6bp deletion	6	6 (100.0 %)	AAD & FS	Yes	Normal flower
4-17	EgLFY-sg1	7bp deletion	NA	7bp deletion	NA	6	0 (0.0 %)	FS & FS	NA	NONE
4-18	EgLFY-sg1	7bp deletion	NA	7bp deletion	NA	6	0 (0.0 %)	FS & FS	NA	NONE
4-88	EgLFY-sg1	13bp deletion	NA	7bp deletion	NA	6	0 (0.0 %)	FS & FS	NA	NONE
4-24	EgLFY-sg1sg2	Inversion (225bp)		12bp deletion	61bp deletion	4	0 (0.0 %)	InvNterm & FS	NA	NONE
4-41	EgLFY-sg1sg2	225bp deletion		1bp insertion	NA	4	0 (0.0 %)	NtermD & FS	NA	NONE
30-19	EgLFY-sg1sg2	226bp deletion		264bp deletion		3	0 (0.0 %)	NtermD & FS	NA	NONE

Methods S1 Target sequence cloning protocol.

To clone the target sequence into the psK-AtU626 backbone, synthesize two oligos of the form:

Oligo 1: 5' – GATTGNNNNNNNNNNNNNNNNNNN – 3'
Oligo 2: 3' – CNNNNNNNNNNNNNNNNNNNCAAA – 5'

Oligo annealing and cloning into backbone vectors:

1. Digest 1µg of psK-AtU626 with BbsI for 30 min at 37C:
   1 µg psK-AtU626
   1 µl FastDigest BbsI (Fermentas)
   1 µl FastAP (Fermentas)
   2 µl 10X FastDigest Buffer
   X µl ddH2O
   ---
   20 µl total

2. Gel purify digested psK-AtU626 using QIAquick Gel Extraction Kit and elute in EB.
3. Phosphorylate and anneal each pair of oligos:
   1 µl oligo 1 (100µM)
   1 µl oligo 2 (100µM)
   1 µl 10X T4 Ligation Buffer* (NEB)
   6.5 µl ddH2O
   0.5 µl T4 PNK (NEB)
   ---
   10 µl total

   Anneal in a thermocycler using the following parameters: 37°C 30 min, 95°C 5 min, and then ramp down to 25°C at 5°C/min.

   * 10X T4 Ligation Buffer containing ATP

4. Set up ligation reaction and incubate at 16C overnight:
   X µl BbsI digested psK-AtU626 from step 2 (50ng)
   1 µl phosphorylated and annealed oligo duplex from step 3 (1:200 dilution)
   5 µl 2X T4 ligation Buffer (NEB)
   X µl ddH2O
   1 µl T4Ligase (NEB)
   ---
   20 µl total

5. Transform only 1/2 of the DNA into bacteria (DH5α) and incubate in 1 mL of LB for 45 min.
6. Plate 100 µL of the bacteria in Amp containing LB.
7. Validate the obtained clone by PCR with primer pair (M13F/oligo 2) or sequencing (seq primer: M13F).

8. The validated psK-AtU626 fragment was digested by KpnI and ClaI and inserted into the corresponding sites of psgR-Cas9-2x35S.

9. For dual targeting, the two pairs of target oligos should be inserted into the mediate vector (psK-AtU626) respectively.
10. The obtained pAtU6-sgRNA-target2 module should be amplified with primers containing the 5'- SalI and 3'-ClaI sites. The sites need to be after the KpnI site in the 5- end and before the SalI in the 3' end.
11. This amplified fragment can be inserted into the SalI and ClaI sites of the the psK-AtU626 containing the first pair of target oligos to make a construct with both of the two customized sgRNAs.
12. The validated psK-AtU626 fragment with two sgRNAs can be digested by KpnI and ClaI and inserted into the corresponding sites of psgR-Cas9-2x35S.
13. The entire psgR-Cas9-2x35S fragment with one or two sgRNAs and the Cas9 transgene can be digested and ligated into the plant expression vector of choice.

Methods S2 Additional methods.

Eucalypt transformation and regeneration

In brief, we wounded and cocultivated sterile WT and FT SP7 leaf tissue with Agrobacterium tumefaciens AGL1 cells previously transformed with the constructs of interest (Weigel & Glazebrook, 2006). After 48 hours of cocultivation, we transferred the explants to callus induction medium (CIM). After one week on CIM, explants were moved to shoot induction medium (SIM) for several months. Individual shoots were collected and placed in shoot elongation medium (SEM) for several weeks. Then, shoots were sampled for DNA isolation and transgene genotyping. PCR-confirmed transgenic events were propagated in shoot multiplication medium (SMM). Last, individual ramets for each PCR-confirmed transgenic event were transferred to rooting media (RM). All the media contained kanamycin (75 mg/L) as the selective agent except for SMM and RM, which had no antibiotic.

Vegetative data analysis

We assessed stem height from soil level to the apex of the main stem (or the highest stem, in cases where there were more than one) for each ramet. Stem diameter was measured at four inches from soil-level with digital calipers. We gauged stem diameter using the diameter of the tallest stem for ramets with more than one stem. Stem height and diameter were recorded a month after moving to the greenhouse before any plants started competing for light. Chlorophyll density, leaf area, and leaf perimeter were recorded two months after moving the plants to the greenhouse. Leaf weight was measured two weeks after that. We used a Soil Plant Analysis Development, SPAD-502, meter (Konica Minolta) to measure relative leaf chlorophyll density. We took three readings from two separate leaves for each ramet. The three readings from each leaf were averaged together. The two average SPAD readings were averaged together prior to statistical analyses. Three different leaves from each ramet were scanned using an HP Scanjet 8200. The leaf area and leaf perimeter of each scanned leaf were calculated using ImageJ (Schneider *et al.*, 2012). The leaf area and leaf perimeter of the three leaves were averaged for the statistical analyses. Dry leaf weight was recorded after desiccating the scanned leaves at 65°C for five days. A weight measurement was taken for each leaf, and the average of the three weights was used for the analyses. We also calculated two derived traits, stem volume index (= tree height*(trunk diameter^2)) and specific leaf weight (= dry leaf weight for each leaf/leaf area for each leaf, also called leaf density). Stem volume index and specific leaf weight are considered non-destructive proxies to biomass yield (Zianis *et al.*, 2005)and leaf photosynthesis (Dornhoff & Shibles, 1970; Criswell & Shibles, 1971).

We used a general linear mixed effects model to determine if there were differences in the means of the vegetative traits between the different control categories than what would be expected from chance alone. The controls included Cas9-control (no sgRNAs), escape-control (no T-DNA inserted but regenerated in the presence of

antibiotic), and wild type controls for the WT trial (Fig. 1a). Both FT-Cas9-controls, FT-only-controls, and one FT-escape-control event were used for the FT trial (Fig. 1b). Because no significant differences for any traits were found among the means of all the types of control groups, to increase statistical power and accuracy we combined all the control categories together for the statistical analyses of both trials (cf. Lee et al., 2017). We then used a general linear mixed effects model to determine if the CRISPR-induced mutations had a greater effect on trait values than expected by chance alone. The models included genotype groups; they were composed of KO events and the control events in the WT trial, and FT-KO events, FT-IFM events, and FT-control events in the FT trial. The genotype groups were considered fixed effects, whereas events within groups were random effects. Analyses were done separately for the WT and FT greenhouse trials. Because FT-in-frame-mutation events in the FT trial had distinct types of mutations, and visual inspection showed that some events had low values of leaf area-related traits compared to FT-KOs and FT-controls, we used a general linear effects model to determine if individual FT-IFM events differed from the group of FT-control events (i.e., two FT-only-control events, six FT-Cas9-control events, and one FT-escape-control event) greater than expected by chance alone. This model included all the FT-control events as well as the individual FT-IFM events as fixed effects. One-on-one comparisons of means were done between the FT-control events and the FT-IFM events. Events, whether FT-control or FT-IFM, that had significantly different means when compared to other events were highlighter with an asterisk (Fig. S15). Residual plots were used to check the equal variance and normality assumptions for all models. We performed all the statistical analyzes using R statistical software v3.6.1 (R Core Team, 2017) and the R packages blme (Dorie, 2015) and stats (R Core Team, 2017). Means were estimated using the R package emmeans (https://CRAN.R-project.org/package=emmeans).

Experimental design of qPCR experiments

The specific genes were the orthologs in *Eucalyptus* of: *FLOWERING LOCUS T* (*FT*), *SQUAMOSA PROMOTER BINDING PROTEIN-LIKE 3* (*SPL3*), *SQUAMOSA PROMOTER BINDING PROTEIN-LIKE 9* (*SPL9*), *CAULIFLOWER* (*CAL*), *FRUITFULL* (*FUL*, there are two in *Eucalyptus*), *APETALA3* (*AP3*), *PISTILLATA* (*PI*), *AGAMOUS* (*AG*), *SHATTERPROOF 2* (*SHP2*), and *SEEDSTICK* (*STK*) (Table S2). The relative gene expression of each gene was determined using the delta-delta-Ct (ddCt) method. All reactions were done in triplicate. Expression was normalized using either *GLYCERALDEHYDE-3-PHOSPHATE DEHYDROGENASE* (*GAPDH*) or the *METALLOTHIONEIN* (*MT*) as the “housekeeping” gene. We designed gene-specific primers (Table S2) by first using the PrimerQuest online tool (Integrated DNA Technologies) and then checking each pair’s specificity in *Eucalyptus grandis* by using Primer-BLAST (Ye *et al.*, 2012). The primer specificity was further tested using standard curve analysis by serial dilutions of cDNA (five 1:2 dilutions) for each gene in triplicate. All primers pairs had amplification efficiencies (E) between 90 and 110% and correlation coefficients (R2) higher than 98%

Peptide alignment

We used the UniProt protein database (The UniProt Consortium, 2019) to collect 19 sequences of homologs of *LFY* corresponding to 13 eudicots, two monocots, one tracheophyte, one conifer, one ginkgo, and one embryophyte. We used Clustal Omega (Madeira *et al.*, 2019) to align the peptide sequences and ESPript 3.0 (Robert & Gouet, 2014) to create the graphic.

Video/Movie S1 X-ray projection of FT-only-control inflorescence. Video showing entire X-ray tomography scans, from bottom to top, of an inflorescence from FT-only-control event FT-30 with three flower buds.

Video/Movie S2 X-ray projection of FT-KO inflorescence. Video showing entire X-ray tomography scans, from bottom to top, of an inflorescence from FT-KO 30-16 with two buds.

References

Criswell JG, Shibles RM. **1971**. Physiological Basis for Genotypic Variation in Net Photosynthesis of Oat Leaves1. *Crop Science* **11**: cropsci1971.0011183X001100040027x.

Dorie V. **2015**. blme: Bayesian Linear Mixed-Effects Models. *URL: https://CRAN. R-project. org/package= blme R package version*: 1–0.

Dornhoff GM, Shibles RM. **1970**. Varietal Differences in Net Photosynthesis of Soybean Leaves1. *Crop Science* **10**: cropsci1970.0011183X001000010016x.

Elorriaga E, Klocko AL, Ma C, Strauss SH. **2018**. Variation in Mutation Spectra Among CRISPR/Cas9 Mutagenized Poplars. *Frontiers in Plant Science* **9**.

Madeira F, Park Y mi, Lee J, Buso N, Gur T, Madhusoodanan N, Basutkar P, Tivey ARN, Potter SC, Finn RD, *et al.* 2019. The EMBL-EBI search and sequence analysis tools APIs in 2019. *Nucleic Acids Research* **47**: W636–W641.

R Core Team. **2017**. *R: A Language and Environment for Statistical Computing*. Vienna, Austria: R Foundation for Statistical Computing.

Robert X, Gouet P. **2014**. Deciphering key features in protein structures with the new ENDscript server. *Nucleic Acids Research* **42**: W320-324.

Sayou C, Nanao MH, Jamin M, Posé D, Thévenon E, Grégoire L, Tichtinsky G, Denay G, Ott F, Peirats Llobet M, *et al.* 2016. A SAM oligomerization domain shapes the genomic binding landscape of the LEAFY transcription factor. *Nature Communications* **7**.

Schneider CA, Rasband WS, Eliceiri KW. **2012**. NIH Image to ImageJ: 25 years of image analysis. *Nature Methods* **9**: 671–675.

Siriwardana NS, Lamb RS. **2012**. A conserved domain in the N-terminus is important for LEAFY dimerization and function in Arabidopsis thaliana. *The Plant Journal* **71**: 736–749.

The UniProt Consortium. **2019**. UniProt: a worldwide hub of protein knowledge. *Nucleic Acids Research* **47**: D506–D515.

Ye J, Coulouris G, Zaretskaya I, Cutcutache I, Rozen S, Madden TL. **2012**. Primer-BLAST: A tool to design target-specific primers for polymerase chain reaction. *BMC Bioinformatics* **13**: 134.

Zianis D, Suomen Metsätieteellinen Seura, Metsäntutkimuslaitos. **2005**. *Biomass and stem volume equations for tree species in Europe*. Helsinki, Finland: Finnish Society of Forest Science, Finnish Forest Research Institute.
